# Case-Control Microbiome Study of Chronic Otitis Media with Effusion in Children Points at Streptococcus salivarius as a Pathobiont-Inhibiting Species

**DOI:** 10.1128/mSystems.00056-21

**Published:** 2021-04-20

**Authors:** Jennifer Jörissen, Marianne F. L. van den Broek, Ilke De Boeck, Wannes Van Beeck, Stijn Wittouck, An Boudewyns, Paul Van de Heyning, Vedat Topsakal, Vincent Van Rompaey, Ine Wouters, Liesbet Van Heirstraeten, Pierre Van Damme, Surbi Malhotra-Kumar, Heidi Theeten, Olivier M. Vanderveken, Sarah Lebeer

**Affiliations:** a Department of Bioscience Engineering, University of Antwerp, Antwerp, Belgium; b Department of Translational Neurosciences, Faculty of Medicine and Health Sciences, University of Antwerp, Wilrijk, Belgium; c Department of Otorhinolaryngology, Head and Neck Surgery and Communication Disorders, Antwerp University Hospital, Edegem, Belgium; d Faculty of Medicine and Health Sciences, University of Antwerp, Wilrijk, Belgium; e Centre for the Evaluation of Vaccination, Vaccine and Infectious Disease Institute, University of Antwerp, Wilrijk, Belgium; f Laboratory of Medical Microbiology, Vaccine and Infectious Disease Institute, University of Antwerp, Wilrijk, Belgium; Teagasc Food Research Centre

**Keywords:** 16S rRNA, *Streptococcus salivarius*, ear canal, microbiome, middle ear, otitis media, otitis media with effusion, pediatric, probiotics, upper respiratory tract

## Abstract

Chronic otitis media with effusion (OME) has been associated with a shift in microbiome composition and microbial interaction in the upper respiratory tract (URT). While most studies have focused on potential pathogens, this study aimed to find bacteria that could be protective against OME through a case-control microbiome study and characterization of isolates from healthy subjects. The URT and ear microbiome profiles of 70 chronic OME patients and 53 controls were compared by 16S rRNA amplicon sequencing. Haemophilus influenzae was the most frequent classic middle ear pathobiont. However, other taxa, especially Alloiococcus otitis, were also frequently detected in the ear canal of OME patients. Streptococci of the *salivarius* group and Acinetobacter lwoffii were more abundant in the nasopharynx of healthy controls than in OME patients. In addition to the microbiome analysis, 142 taxa were isolated from healthy individuals, and 79 isolates of 13 different *Streptococcus* species were tested for their pathobiont-inhibiting potential. Of these, Streptococcus salivarius isolates showed a superior capacity to inhibit the growth of H. influenzae, Moraxella catarrhalis, Streptococcus pneumoniae, Streptococcus pyogenes, Staphylococcus aureus, A. otitis, and Corynebacterium otitidis. S. salivarius strains thus show potential as a probiotic for prevention or treatment of OME based on their overrepresentation in the healthy nasopharynx and their ability to inhibit the growth of respiratory pathobionts. (This study has been registered at ClinicalTrials.gov under registration no. NCT03109496.)

**IMPORTANCE** The majority of probiotics marketed today target gastrointestinal health. This study searched for bacteria native to the human upper respiratory tract, with a beneficial potential for respiratory and middle ear health. Comparison of the microbiomes of children with chronic otitis media with effusion (OME) and of healthy controls identified Streptococcus salivarius as a health-associated and prevalent inhabitant of the human nasopharynx. However, beneficial potential should be assessed at strain level. Here, we also isolated specific S. salivarius strains from the healthy individuals in our study. These isolates showed a beneficial safety profile and efficacy potential to inhibit OME pathogens *in vitro*. These properties will now have to be evaluated and confirmed in human clinical studies.

## INTRODUCTION

Otitis media encompasses a spectrum of disease conditions characterized by accumulation of effusion in the middle ear cavity. Otitis media with effusion (OME) is characterized by the presence of effusion behind an intact tympanic membrane in the absence of other signs or symptoms of acute inflammation (1). Chronic OME, lasting ≥3 months, is typically treated by placement of ventilation tubes into the tympanic membrane (2). Middle ear health is closely associated with upper respiratory tract (URT) health. Bacteria traditionally isolated from OME middle ear effusion are nontypeable (unencapsulated) Haemophilus influenzae, Streptococcus pneumoniae, and Moraxella catarrhalis, which typically inhabit the URT (3). In addition to these classic otopathogens, multiple 16S rRNA gene sequencing studies of OME middle ear effusion also reported high levels of Alloiococcus otitis, Corynebacterium otitidis (formerly Turicella otitidis [4]), Pseudomonas spp., and Staphylococcus spp. (Staphylococcus aureus, Staphylococcus auricularis, and Staphylococcus epidermidis) (5–16). It is, however, still uncertain if these taxa, many of which are residents of the ear canal (17, 18) or the healthy URT (19), contribute to middle ear disease. Long-term perturbation of the microbiota has been associated with several chronic inflammatory diseases (20) and is also hypothesized to underlie chronic OME. Preventing such perturbation or restoring a perturbed microbiota through addition of probiotics, i.e., live microorganisms that, when administered in adequate amounts, confer a health benefit on the host (21), could be a valuable method for OME prevention and could reduce the need for surgical intervention. Such a probiotic approach is widely used for the gastrointestinal tract but is underexplored for respiratory health or the prevention and treatment of otitis media (22). The first intervention studies testing local bacteriotherapy to prevent or cure respiratory tract infections used alpha-hemolytic streptococci (AHS) of the *mitis* and *sanguinis* groups (23, 24), as they are among the first colonizers of the human URT after birth (25). Early studies specifically targeting middle ear health also used AHS isolated from the URT of healthy individuals. A nasal spray containing 2 Streptococcus sanguinis strains, 1 Streptococcus mitis strain, and 1 Streptococcus oralis strain isolated from the opening of the Eustachian tube of healthy children reduced the rate of acute otitis media (AOM) and OME when participants were pretreated with antibiotics (26), but not when antibiotic treatment was omitted (27). One study, which compared the effect of a nasal spray containing either S. sanguinis 89a or Lacticaseibacillus rhamnosus LB21 on chronic OME, found a stronger effect for S. sanguinis, although both strains reduced effusion (28). A recent bacterial intervention study using Ligilactobacillus salivarius PS7 isolated from human milk also reported reductions in the risk of experiencing at least one episode of AOM by 49% and in the length of AOM episodes from 6 to 4 days in otitis-prone children. However, it is unclear whether this effect was achieved through local or systemic mechanisms, as the formulation was not described, and colonization of the URT was not measured (29). To date, some of the best documented commercially available probiotics targeting URT and oral health are Streptococcus salivarius strains K12, M18, and 24SMB and S. oralis 89a (30–41). *S. salivarius* K12 may reduce AOM by up to 71.5% and was also found to reduce adenoid and tonsil hypertrophy and cause a clearing of middle ear effusion (32–36), although one study observed no difference in AOM episodes compared to untreated controls (37). A nasal spray combining *S. salivarius* 24SMB and S. oralis 89a was found, by dedicated quantitative PCR (qPCR), to reduce the number and severity of AOM episodes in otitis-prone children, especially when *S. salivarius* 24SMB colonized the respiratory tract (38–40), and to reduce adenoid hypertrophy and middle ear effusion in children with chronic OME, with significant reduction in need for surgery from 90.9% of the children in the control group to 27.3% in the treatment group (*n* = 22 in each group) (41). While many of the studies described above show promising effects on AOM and OME, the selection of the tested strains was based on early cultivation results, without insights into the resident microbiome.

Here, we aimed to identify bacterial taxa with a potentially protective effect against OME starting from a microbiome-sequencing approach comparing the URT and ear microbiome of children with and without chronic OME. This comparison was used to guide the subsequent cultivation approach and phenotyping, focusing on the ability of URT isolates from healthy individuals to inhibit the growth of classic middle ear pathobionts, as well as on underexplored potential OME-associated pathobionts that were identified in the microbiome comparison.

## RESULTS

### Study population and sample characteristics.

**FIG 1:**
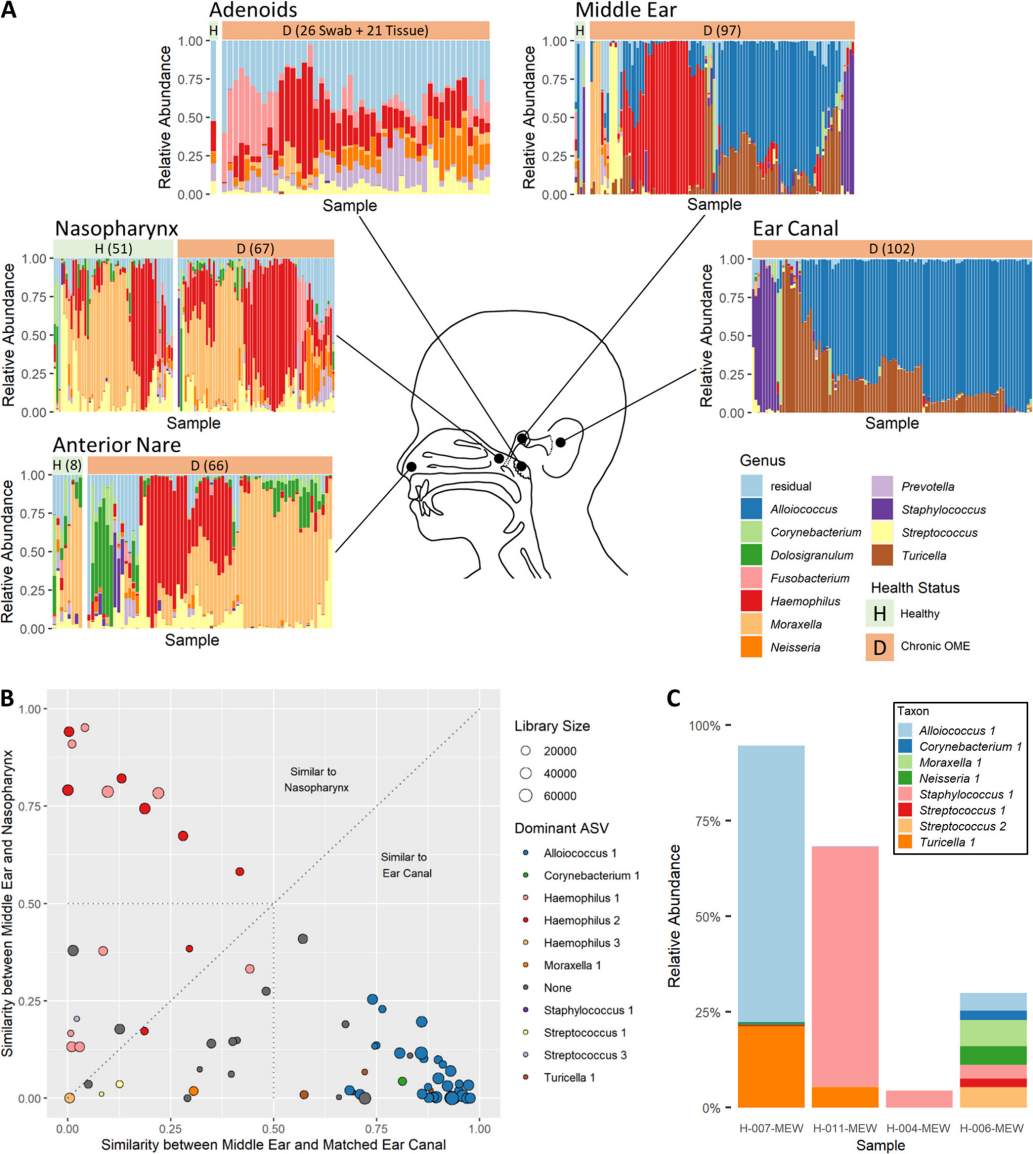
The bacterial microbiome of the human upper respiratory tract and ears. (A) Genus-level microbiome composition of multiple upper respiratory tract and ear niches in health (“H”) and during chronic otitis media with effusion (“D”). Each bar represents one sample, and the full height of a bar represents 100% of reads. The 11 most abundant genera across all samples are shown, with all other genera summarized under “Residual.” Health status is indicated by header bar color and letter, and the number of successfully sequenced samples per location and group is provided where possible. For the healthy adenoid swabs and healthy middle ear rinses, 1 and 4 samples were available for analysis, respectively. Adenoid swabs and tissue samples were grouped into one graph, as they did not differ significantly. Note that by “healthy,” we refer to the absence of inflammation and infection at the studied anatomical site. For the nasopharynx, children attending day care were included, in addition to cochlear implant recipients who were sampled as reference for the other anatomical sites. (B) Similarity (1 − Bray-Curtis dissimilarity) of middle ear effusion samples to matched nasopharynx versus side-matched ear canal samples. A high score indicates high similarity between two locations. Symbols are sized based on the number of reads remaining in the middle ear sample after contaminant filtering. Colors indicate which ASV is dominant (>50% relative abundance) in the middle ear. (C) Stacked bar charts of healthy middle ear samples. Only ASVs detected in more than one sample are shown.

URT samples were collected from 70 OME patients and two control groups with no sign of otitis media or respiratory infections, namely (i) 12 cochlear implant recipients and (ii) 41 children attending day care. Participant characteristics and locations sampled are summarized in Table 1 and shown in Fig. 1A. There was no significant difference in gender ratio (both 41% females) or age (Student’s *t* test, *P* = 0.12) between the OME group and the combined control group. Gender had no significant effect on microbiome composition, while age only influenced the anterior nares microbiome (permutational multivariate analysis of variance [PERMANOVA], *P* = 0.002 in the OME group and *P* = 0.043 in the cochlear implant group). In total, 66 OME patients received tympanostomy tubes in both ears, and 28 patients underwent adenoidectomy. Neither laterality (unilateral versus bilateral OME) nor adenoidectomy significantly influenced the microbiome composition of any of the sampled locations. Of 523 low-biomass URT samples sequenced, only 443 samples had at least twice the number of reads compared to the largest negative control after removing obvious contaminants (see Tables S1 and S2 in the supplemental material). Final library sizes ranged from 2,500 to 784,460 reads, with a mean (± standard deviation [SD]) of 36,332 ± 49,509 reads.

**TABLE 1:** Participant characteristics and sampled locations by group

Characteristic	Controls			Chronic OME patients
	Cochlear implant recipients	Children attending day care	All	
No. of participants	12	41	53	70
Age in yrs (mean ± SD)	9.01 ± 9.74	1.58 ± 8.38	4.56 ± 7.21	4.38 ± 2.42
% Female	42	41	41	41
Sampled locations^*a*^	AN, NP, ME	NP	AN, NP, ME	AN, NP, Ad, Ad, ME, ECa

a AN, anterior nares; NP, nasopharynx; Ad, adenoids; ME, middle ear; EC, ear canal.

10.1128/mSystems.00056-21.2 TABLE S1 Number of patients enrolled and numbers of samples which could have been collected, were collected, and passed quality control (QC) after sequencing. CI, cochlear implant recipients; DC, children attending day care. Download Table S1, DOCX file, 0.02 MB. Copyright © 2021 Jörissen et al. 2021 Jörissen et al. https://creativecommons.org/licenses/by/4.0/ This content is distributed under the terms of the Creative Commons Attribution 4.0 International license.

10.1128/mSystems.00056-21.3 TABLE S2 Taxa detected in negative extraction and PCR controls and action taken. Download Table S2, PDF file, 0.2 MB. Copyright © 2021 Jörissen et al. 2021 Jörissen et al. https://creativecommons.org/licenses/by/4.0/ This content is distributed under the terms of the Creative Commons Attribution 4.0 International license.

### Identification of bacteria dominant in OME middle ear effusion.

To identify potential bacterial pathogens for chronic OME in children resident in Flanders, Belgium, we analyzed 97 OME middle ear effusion aspirates of 59 children. Of these, 80% were dominated (≥50% relative abundance) by a single amplicon sequence variant (ASV) (Table 2). In just 33% of effusions, the dominant ASV belonged to one of the classic otopathogen genera Haemophilus, Moraxella, or Streptococcus, but these genera showed high prevalence, with detection in 75%, 53% and 56% of middle ear samples, respectively. Other dominant ASVs were Alloiococcus 1 (39%), Turicella 1 (4%), Staphylococcus 1 (3%), and Corynebacterium 1 (1%) (Table 2).

**TABLE 2:** Prevalence of ASVs dominant in at least one middle ear effusion

ASV	Species	Middle ear		Ear canal	
		Prevalence (%)	Dominance (%)^*a*^	Prevalence (%)	Dominance (%)^*a*^
*Alloiococcus* 1	Alloiococcus otitis	87.6	39.2	90.2	75.5
*Haemophilus* 1	Haemophilus influenzae	56.7	14.4	46.1	0
*Haemophilus* 2	Haemophilus aegyptius	42.3	10.3	22.5	0
*Streptococcus* 3	Streptococcus pyogenes	4.1	1.0	2.9	0
*Streptococcus* 1	Streptococcus pneumoniae */* Streptococcus pseudopneumoniae ^*b*^	14.4	2.1	4.9	0
*Corynebacterium* 1	Corynebacterium pseudodiphtheriticum */* Corynebacterium propinquum	8.2	1.0	13.7	1.0
*Haemophilus* 3	Haemophilus quentini/H. influenzae	10.3	1.0	1.0	0
*Moraxella* 1	Moraxella catarrhalis/Moraxella nonliquefaciens	47.4	4.1	43.1	0
*Staphylococcus* 1	Not resolved to species level	51.5	3.1	69.6	4.9
*Turicella* 1	Corynebacterium otitidis	71.1	4.1	81.4	7.8

a Dominance was defined as the percentage of samples with a relative abundance of ≥50%.

b ASV represents both listed species.

### Origin of bacteria dominant in OME middle ear effusion.

For 73 effusion samples of 49 patients, matched nasopharynx and ear canal swabs were sequenced successfully. To explore body site continuity, we plotted the similarity (1 − Bray-Curtis dissimilarity) between the effusion and nasopharynx microbiome against the similarity between the effusion and the side-matched ear canal microbiome (Fig. 1B). All effusion samples dominated by *Alloiococcus*, Turicella, *Staphylococcus*, or *Corynebacterium* were more similar to the ear canal (similarity score, ≥0.574) than to the nasopharynx (similarity score, ≤0.254). These taxa were also frequently dominant in the ear canals of all patients (Fig. 1A), indicating that they probably originated from the ear canal.

### The microbiome of the healthy middle ear cavity.

Twelve middle ear rinses collected from microbiologically healthy cochlear implant recipients were sequenced to identify bacteria associated with middle ear health. However, only four of these were retained after quality filtering. In addition, of the 107 ASVs detected in the remaining four samples, only seven were present in multiple samples (Fig. 1C), and of these, only *Streptococcus* 1 and *Corynebacterium* 1 (Table 2) were absent from the negative controls.

### Comparison of the nasopharynx microbiome in health and during chronic OME.

**FIG 2:**
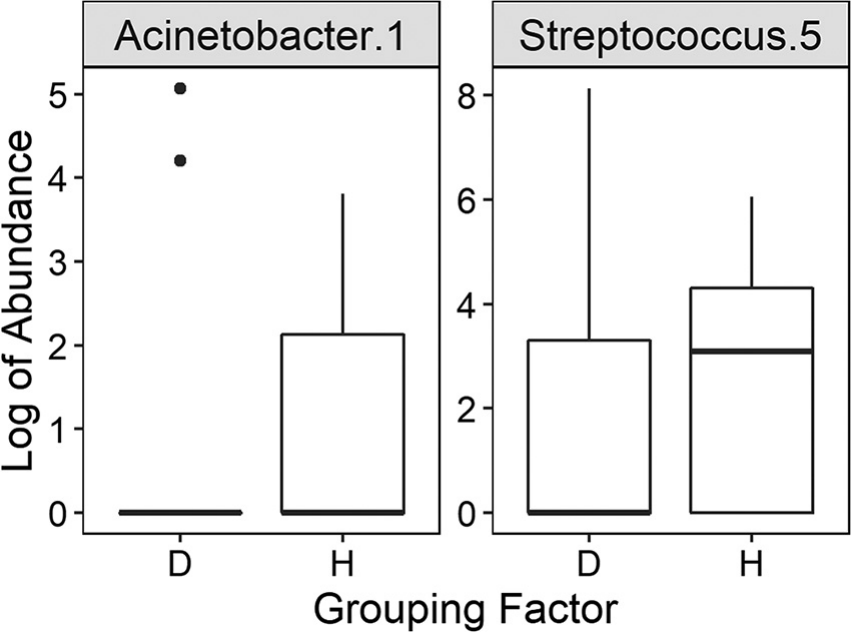
ASVs significantly differentially abundant in the nasopharynx of chronic OME (“D”) versus controls (“H”) by Analysis of Composition of Microbiomes (ANCOM) analysis with stringent correction for multiple testing (see Fig. S1 in the supplemental material for an alternative analysis approach).

Because of the high risk of bias by contaminants and the low number of successfully sequenced samples of the middle ear, we decided to focus on the nasopharynx. This enabled us to compare the URT microbiomes of OME patients and of healthy controls and to identify health-associated bacteria. For this analysis, data from the nasopharynx swabs from 67 chronic OME patients, 10 microbiologically healthy cochlear implant recipients, and 41 healthy children attending day care were included. The nasopharynx microbiome differed significantly between OME patients and healthy controls (PERMANOVA, *P* = 0.014), a difference which was more pronounced than that observed between the cochlear implant and the day care control groups (PERMANOVA, *P* = 0.049). A total of 134 taxa were shared between both control groups and the OME group (see Table S3 in the supplemental material). Differential abundance analysis (Analysis of Composition of Microbiomes [ANCOM] [42]) with stringent correction for multiple testing identified Acinetobacter 1 (Acinetobacter lwoffii or Acinetobacter pseudolwoffii) and *Streptococcus* 5 (*S. salivarius*, S. thermophilus, or Streptococcus vestibularis) as health associated, since the Aitchison’s log ratio of the abundance of these ASVs to other ASVs was significantly higher in the healthy group than in the OME group (Fig. 2; see also Fig. S1 in the supplemental material). When searching for taxa specific for OME, no taxon was significantly more abundant in the nasopharynx of OME patients compared to controls, suggesting that we should focus on health-associated rather than OME-specific taxa for further functional analyses.

10.1128/mSystems.00056-21.4 TABLE S3 Prevalence and mean relative abundance of nasopharynx amplicon sequence variants (ASVs) shared between otitis media with effusion (OME) patients and healthy controls. The healthy controls were considered cumulatively (HNP_All_) and individually (HNP_CI_, cochlear implant recipients; HNP_DC_, children attending day care). To calculate the mean relative abundance, only samples in which the taxon was present was considered. Download Table S3, PDF file, 0.3 MB. Copyright © 2021 Jörissen et al. 2021 Jörissen et al. https://creativecommons.org/licenses/by/4.0/ This content is distributed under the terms of the Creative Commons Attribution 4.0 International license.

10.1128/mSystems.00056-21.7 FIG S1 Compositional differential abundance of nasopharynx amplicon sequence variants (ASVs) between chronic otitis media with effusion (OME) patients and healthy controls. Each tile shows an estimate of the differential abundance of an ASV on the *y* axis relative to an ASV on the *x* axis. A plus/minus sign means that the ASV is significantly more/less abundant in healthy controls compared with OME patients, with a false discovery rate of 10% (capped with the method of Benjamini and Yekutieli). The data set was rarefied to the size of the smallest sample (2,820 reads) before performing a Wilcoxon rank-sum test, and only taxa that were differentially abundant in comparison to at least one other taxon are shown. Download FIG S1, TIF file, 0.5 MB. Copyright © 2021 Jörissen et al. 2021 Jörissen et al. https://creativecommons.org/licenses/by/4.0/ This content is distributed under the terms of the Creative Commons Attribution 4.0 International license.

### Isolation of bacteria from the healthy human URT.

We next aimed to characterize the potentially beneficial properties of bacteria isolated from healthy controls in more detail, especially with their potential to control the growth of middle ear pathobionts. We isolated 142 bacterial isolates belonging to 11 different genera from anterior nare (*n* = 8), nasopharynx (*n* = 9), and adenoid swabs (*n* = 1) from 9 microbiologically healthy cochlear implant recipients (see Fig. S2 in the supplemental material). *Streptococcus* spp. were most frequently isolated (*n* = 66), especially from the nasopharynx, while A. lwoffii and A. pseudolwoffii, which we identified as potential beneficial members based on ANCOM analysis, were not cultivated, likely due to their low relative abundance (0.1%) in cochlear implant controls (Table S3). All *Streptococcus* isolates from healthy children belonged to either the *mitis* group (*n* = 28), the *salivarius* group (*n* = 32), or the *sanguinis* group (*n* = 5).

10.1128/mSystems.00056-21.8 FIG S2 Overview of bacterial isolates obtained from the upper respiratory tract of healthy children. (A) Distribution of isolated species by anatomic location, colored by genus. (B) Number of strains per species isolated from each patient. (C) Distribution of genera by anatomic location. Download FIG S2, TIF file, 0.8 MB. Copyright © 2021 Jörissen et al. 2021 Jörissen et al. https://creativecommons.org/licenses/by/4.0/ This content is distributed under the terms of the Creative Commons Attribution 4.0 International license.

### Antimicrobial activity of streptococci against classic otopathogens.

**FIG 3:**
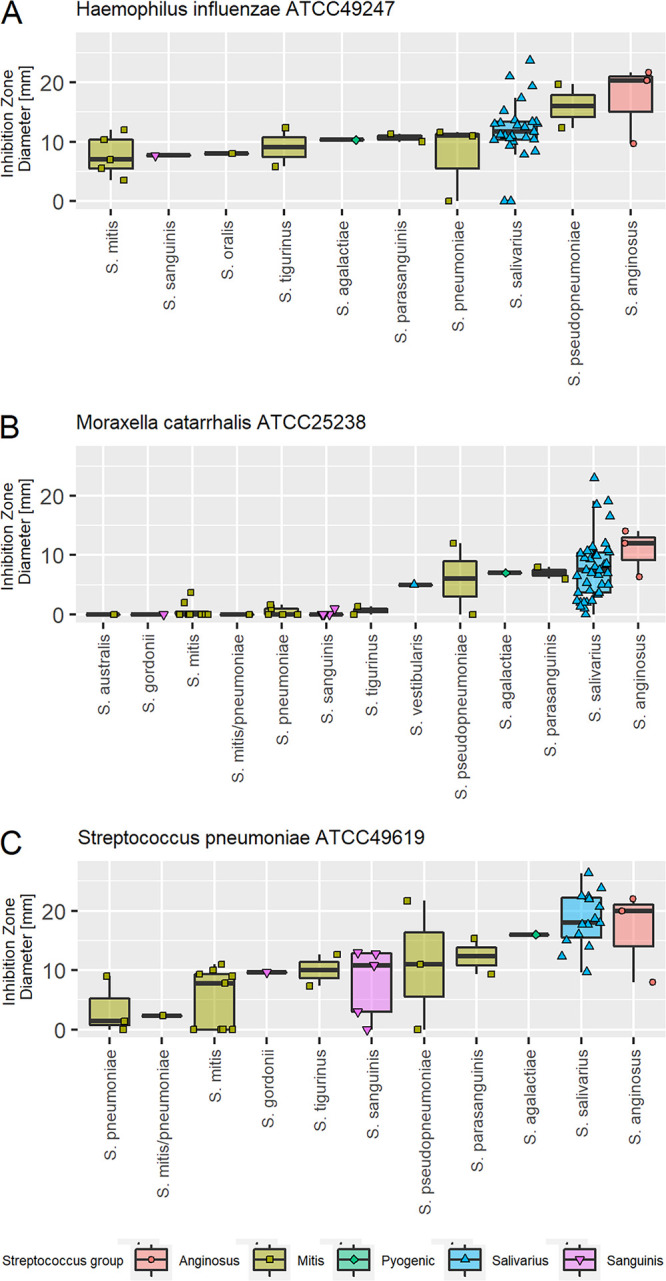
Mean inhibition of classic otopathogens by Streptococcus species isolated from the URT of healthy children and adults, as measured through the spot assay method. Each point represents a different isolate.

**FIG 4:**
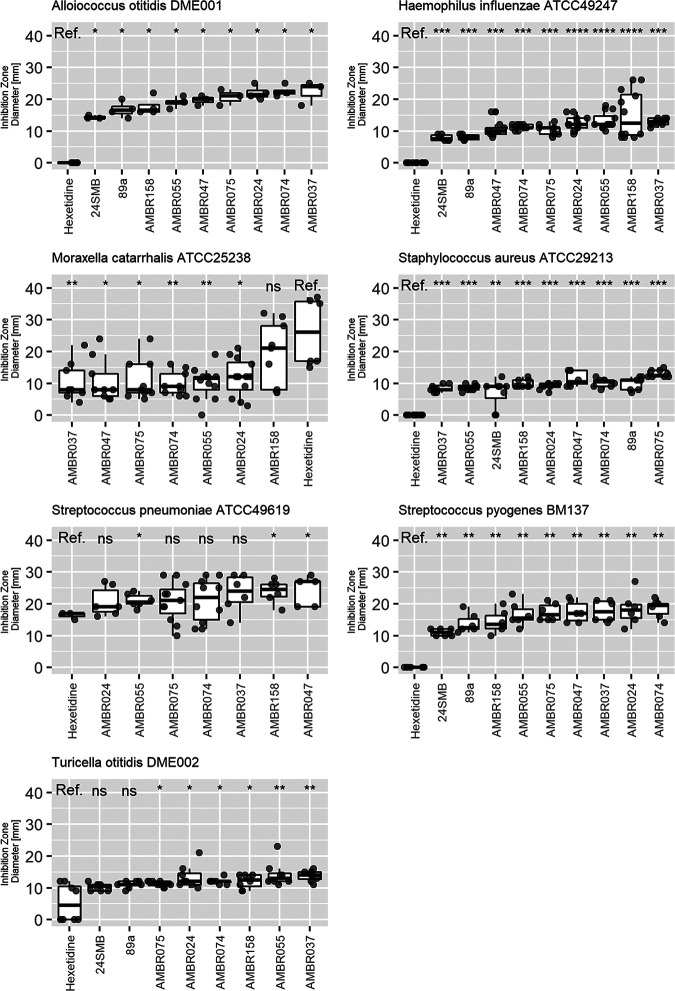
Inhibition of upper respiratory tract and classic and suspected otopathogens by 7 *S. salivarius* isolates, as measured through the spot assay method. Isolates were compared to *S. salivarius* 24SMB and S. oralis 89a, isolated from the Rinogermina probiotic nasal spray, and Hextril mouthwash (0.1% hexetidine) served as a positive control. For each pathobiont, the tested isolates are sorted from smallest (left) to largest (right) mean inhibition zone diameter. Statistical comparison of inhibition zones was performed with 0.1% hexetidine as a reference. ns, *P* > 0.05; *, *P* < 0.05; **, *P* < 0.01; ***, *P* < 0.001; ****, *P* < 0.0001.

In the present study, *salivarius* group streptococci were identified as health associated, but other research groups have also associated other commensal streptococci with respiratory and oral health and confirmed that they are safe in human clinical trials (26, 28, 43–45). We therefore screened 78 commensal streptococci (53 from this study and 25 isolated from healthy adults [19, 46]) for their antimicrobial activity against the three classic otopathogens. Based on results obtained with spot assays, in which the pathogen and the potentially beneficial bacteria were cocultured, all tested species could inhibit the growth of H. influenzae, with Streptococcus anginosus, Streptococcus pseudopneumoniae, and *S. salivarius* isolates showing the largest inhibition zones on average (17 ± 5 mm, 16 ± 4 mm, and 13 ± 4 mm, respectively [mean ± SD]), although two *S. salivarius* isolates had no effect (Fig. 3A). S. anginosus (9 ± 4 mm) and *S. salivarius* (9 ± 6 mm) could also inhibit M. catarrhalis, but this effect was strain dependent (Fig. 3B). *S. salivarius* (19 ± 5 mm) and S. vestibularis (19 ± 3 mm) were most effective against S. pneumoniae, with *S. anginosus* in third place (Fig. 3C). Based on the spot assays and the results of the differential abundance analysis, we selected seven *S. salivarius* isolates (AMBR024, AMBR037, AMBR047, AMBR055, AMBR074, AMBR075, and AMBR158) for more detailed antimicrobial screening. These isolates were additionally tested against the URT pathobionts Streptococcus pyogenes BM137 and Staphylococcus aureus ATCC 29213 and the suspected middle ear pathobionts *A. otitis* AMBR153 and *C. otitidis* AMBR154 isolated from OME middle ear effusion during this study. *S. salivarius* 24SMB and S. oralis 89a isolated from the probiotic nasal spray Rinogermina (DMG Italia) were used as references. All isolates could inhibit all tested pathobionts in spot assays, whereby AMBR158 was the most effective isolate against the classic otopathogen genera H. influenzae, M. catarrhalis, and S. pneumoniae (Fig. 4). Screening of the genomes against the secondary metabolite databases AntiSMASH 5.0 (47) and BAGEL4 (48) revealed that all seven in-house isolates harbored a bacteriocin-like peptide (*blp*) cassette with different predicted bacteriocins, ABC transporters, and immunity proteins (Table 3) (49, 50). AMBR037, AMBR074, and AMBR075 additionally encoded a lactococcin 972 family bacteriocin, including an ABC transporter and an immunity mechanism (49). Lantipeptide loci were detected in the genomes of AMBR055 (related to salivaricin A2 [50] and salivaricin 9 [51]) and AMBR074 (related to streptococcin A M49 [52] and macedocin [53]). Lasso peptides were detected in AMBR024 (undetermined) and AMBR158 (related to streptomonomicin [54]). In addition to bacteriocins, antiSMASH (18) also predicted a gramidicin nonribosomal peptide synthetase (NRPS) locus in AMBR024 (Table 3).

**TABLE 3:** Genetic loci encoding potentially antimicrobial secondary metabolites

Isolate	Most closely related bacteriocin type				
	Class I		Class II		NRPS^*a*^
	Lantipeptides (Ia)	Lasso peptides (If)	IIc (*blp* locus)	IId	
AMBR024		Lasso peptide	Mutacin IV		Gramicidin
AMBR037			Lactococcin	Lactococcin 972	
AMBR047			Lactococcin		
AMBR055	Salivaricin A2/salivaricin 9		Lactococcin and gassericin		
AMBR074	Streptococcin A		Lactococcin and Thermophilin A	Lactococcin 972	
	M49/macedocin				
AMBR075			Lactococcin	Lactococcin 972	
AMBR158		Streptomonomicin	Thermophilin A		

a NRPS, nonribosomal peptide synthetase.

### Initial evaluation of antibiotic resistance and virulence genes.

Potential probiotics should not carry transferable antibiotic resistance markers (55), as these could be transmitted to pathogens, complicating their treatment. We therefore screened the isolate genomes for transferable antibiotic resistance markers (see Table S4 in the supplemental material) and tested their susceptibility to key antibiotic classes *in vitro*. *S. salivarius* AMBR055 and AMBR047 were predicted to harbor the adjacent genes *mef*(A) (100% coverage with 96% identity) and *mel* [also known as *msr*(D); 100% coverage with 100% identity], which encode efflux pumps for macrolide class antibiotics. These genes are part of the mobile genetic element *mega* (macrolide efflux genetic assembly), which has been shown to be transferable to S. pneumoniae via transformation (56–58). Phenotypic testing indicated only resistance for AMBR047 against the macrolide erythromycin (MIC of 4 to 16 mg/liter with a cutoff of 2 mg/liter) and against chloramphenicol (MIC of 8 mg/liter with a cutoff 4 mg/liter). Based on this finding, AMBR055 and AMBR047 were excluded from further analysis.

10.1128/mSystems.00056-21.5 TABLE S4 Antibiotic resistance and virulence factor genes. Download Table S4, DOCX file, 0.02 MB. Copyright © 2021 Jörissen et al. 2021 Jörissen et al. https://creativecommons.org/licenses/by/4.0/ This content is distributed under the terms of the Creative Commons Attribution 4.0 International license.

As an additional safety check, we also verified that the potential probiotics did not carry any known virulence factors. No virulence genes of concern were observed, although two genes showed a hit with the Virulence Factor Database (VFDB) (14) (Table S4) for all isolates, namely, *psaA*, encoding a putative adhesin (with 87.85% to 88.92% coverage of and 76.24% to 76.96% identity to the pneumococcal surface adhesion gene of S. pneumoniae TIGR4), and *hasC*, encoding UDP-glucose pyrophosphorylase (91.26% to 91.85% coverage and 76.94% to 77.67% identity to the gene of S. pyogenes M1 GAS), which is probably involved in capsular polysaccharide biosynthesis. Sufficient adhesion is considered a desired property for most probiotic applications because it can promote persistence in the niche (59). *S. salivarius* produces a levan or dextran capsule instead of the known virulence factor hyaluronic acid capsule found in pathogenic S. pyogenes that mimics human connective tissue (60, 61). These two conserved genes were therefore not considered virulence factors, but rather adaptation factors, reflecting adaptation of *S. salivarius* to the URT rather than pathogenicity.

### Ability of *S. salivarius* to adhere to respiratory epithelium.

**FIG 5:**
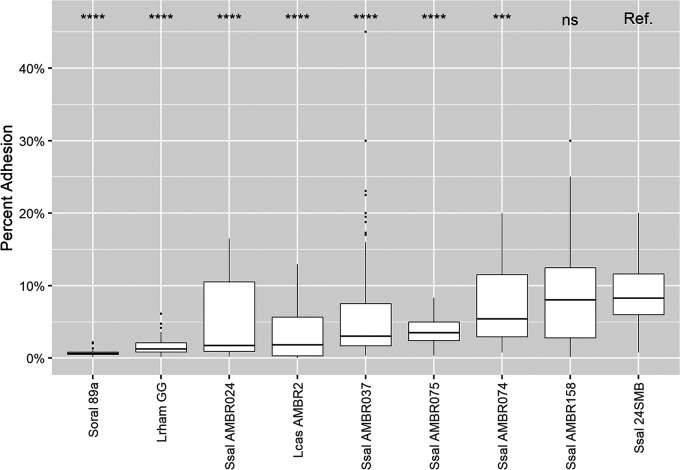
Adhesion of bacterial isolates to respiratory epithelial cells (Calu-3). The adhesion of Streptococcus salivarius (Ssal) isolates obtained in the present study was statistically compared to that of *S. salivarius* 24SMB (38–40) of the Rinogermina probiotic nasal spray using an unpaired Wilcoxon rank sum test. S. oralis (Soral) 89a was also isolated from Rinogermina, but it showed a lower adhesion capability than all *S. salivarius* strains. *Lacticaseibacillus casei* (Lcas) AMBR2 and *Lacticaseibacillus rhamnosus* (Lrham) GG were included as examples of lactobacilli with a higher and a lower ability to adhere to respiratory epithelium, respectively (64). Isolates are sorted from lowest to highest median adhesion percentage. ns, *P* > 0.05; **, *P* < 0.01; ***, *P* < 0.001; ****, *P* < 0.0001. *P* values are adjusted by the Holm method.

Since sufficient adhesion to the host mucosa or epithelial cells is known to increase a probiotic’s opportunity to interact with its host and mediate displacement of already adhered bacteria and competitive exclude pathogens that bind to the same receptor (62, 63), we also phenotypically characterized the interaction of the five remaining isolates with the respiratory epithelial Calu-3 cells. As examples of taxa with a higher and lower ability to adhere to respiratory epithelium, we also included the gastrointestinal probiotic Lacticaseibacillus rhamnosus GG (median adhesion of 1.2%) and the URT-derived investigational new probiotic Lacticaseibacillus casei AMBR2 (1.9%) (64). All five *S. salivarius* isolates could adhere to the cells, with median adhesion values between 1.8% (AMBR024) and 8.1% (AMBR158), the latter of which was statistically (Wilcoxon rank sum test, *P* > 0.05) as adherent as the commercial URT probiotic *S. salivarius* 24SMB (8.3%) (Fig. 5). In contrast, the commercial URT probiotic S. oralis 89a adhered with a median adhesion value of 0.6%, even lower than that of L. rhamnosus GG.

To investigate the observed differences in adhesion, we manually screened the five *S. salivarius* isolate genomes annotated by The PATRIC Comprehensive Genome Analysis pipeline (65) and by Operon-mapper (66) for genes associated with adhesion. Apart from the *psaA* gene mentioned above, all isolates also harbored two copies of the *peb1A* gene, which is annotated as major cell-binding factor in the Gram-negative bacterium Campylobacter jejuni (67). All of our isolates also encoded homologues of SalivA_1472 and SalivA_1475, known genes for adhesion of *S. salivarius* JIM87777 (68, 69), although the SalivA_1475 homologue of AMBR047 appeared to be truncated. AMBR055, AMBR024, and AMBR047 also encoded a homologue of SalivA_1473 (zinc metalloprotease ZmpB precursor) (68, 69), which was absent from the other isolates. No isolate encoded a full set of the *srpA*, *srpB*, and *srpC* genes encoding accessory Sec-dependent serine-rich glycoprotein adhesins (SalivA_1458 to SalivA_1456). Indeed, AMBR055 encoded *srpA* and *srpC*, while AMBR024 and AMBR047 both encoded *srpB* an and *srpC*. Of note, *srpB* is the only gene associated with adhesion by both studies (68, 69) (Fig. S3). Although differences between isolates are thus observed, no clear conclusions about the various adhesion capacities can be made based on the isolate genomes, and the gene-function relations remain to be further substantiated in follow-up work.

10.1128/mSystems.00056-21.9 FIG S3 Alignment of a genomic region with 6 genes associated with adhesion in S. salivarius JIM8777 (each highlighted with a square) using the PATRIC Compare Region Viewer tool. Homologues are indicated by the same color, with the exception of the AMBR055 region (marked with an asterisk (*). The original Compare Region Viewer alignment stopped after the homologue of SalivA_1458, as the homologue of Saliva_1457 is missing. A new alignment was started using the SalivA_1456, of which the homologue is present in AMBR055 (here indicated in red) caused a fresh assignment of colors. SALIVA_1456 to SALIVA_1458 = accessory Sec-dependent serine-rich glycoprotein adhesin genes *srpC*, *srpB* and *srpA*. SALIVA_1472 and SALIVA_1475 = YSIRK signal domain/LPXTG anchor domain surface protein. SALIVA_1473 = zinc metalloprotease ZmpB precursor. Download FIG S3, TIF file, 0.9 MB. Copyright © 2021 Jörissen et al. 2021 Jörissen et al. https://creativecommons.org/licenses/by/4.0/ This content is distributed under the terms of the Creative Commons Attribution 4.0 International license.

## DISCUSSION

This study aimed to characterize the microbiome of the URT and ear during chronic OME as a representative chronic inflammatory childhood disease versus healthy control samples of children attending day care (nasopharynx) or receiving a cochlear implant (access to the middle ear). For this microbiome comparison, special attention was given to the identification and characterization of taxa that were more prevalent and abundant in healthy control samples in order to identify potential novel probiotic species and strains.

First, we identified bacteria associated with middle ear disease. Of the classic otopathogen genera, H. influenzae was most frequently dominant, with few effusion samples harboring high levels of M. catarrhalis or S. pneumoniae. Surprisingly, the microbiome of almost half of all effusion samples consisted of typical ear canal bacteria, with A. otitis most frequently dominant, despite the great care that was taken not to touch the ear canal during sampling. Other authors who sequenced the middle ear effusion microbiome of OME (6–8, 13) or recurrent acute otitis media (rAOM) (70) reported similar findings. OME occurs by definition behind an intact tympanic membrane (1), but patients may have had unidentified perforations in the past or inflammation that caused microlesions that could allow bacteria to translocate into the middle ear (7, 8).

Attempts to sequence the microbiome of the healthy middle ear were considered unsuccessful, indicating that very few or even no bacteria were present in this body site under healthy conditions. This is in accordance with a recent study which argued that bacterial signals detected in this body site under healthy conditions are likely due to contamination (71). However, typical middle ear pathobionts colonize the nasopharynx before ascending via the Eustachian tube into the middle ear. Therefore, we hypothesize that URT probiotics targeting the nasopharynx could be used to reduce the incidence or severity of otitis media. Differential abundance analysis identified two taxa (ASVs) as significantly more abundant in the healthy nasopharynx than in the nasopharynx of chronic OME patients, A. lwoffii and *salivarius* group streptococci.

A. lwoffii (recently split into A. lwoffii and A. pseudolwoffii [72]) has been shown to decrease allergic reactions in human dendritic cells and mouse models by inducing a weak T_H_1-type immune response. The mice also produced less mucus-producing goblet cells in their lungs upon sensitization with ovalbumin (73). Allergies are also a risk factor for OME (74). Furthermore, goblet cells and mucous glands are responsible for mucin production in the middle ear, with high densities observed during chronic OME (75). Protection from allergies could decrease the risk of developing OME, and reduction of mucin production could decrease its severity. Whether A. lwoffii has a similar effect on the human middle ear as on mouse lungs remains to be determined. Here, we decided to focus on *S. salivarius* isolates as potential URT probiotics due to their high prevalence and safety history. The Streptococcus salivarius group encompasses the species *S. salivarius*, *S. vestibularis*, and S. thermophilus. The latter is an industrially used dairy fermenter with generally recognized as safe (GRAS) and qualified presumption of safety (QPS) status and which is also marketed as a gastrointestinal probiotic (76). In contrast, *S. vestibularis* and *S. salivarius* are mainly found in the human oral cavity (77). Since the oral cavity is in direct contact with the URT, it can be rationalized that translocation from the oral cavity to the nasopharynx can occur, as our research group recently also suggested for members of the Lactobacillaceae (64). Similarly to the present study, Walker et al. (78) identified a taxon related to S. thermophilus as significantly more abundant in the anterior nares of healthy controls than in those of chronic OME patients through differential abundance analysis with DESeq2 (79). At least three isolates of *S. salivarius* are already marketed as probiotics, namely (i) *S. salivarius* K12, which mainly targets pharyngotonsillitis caused by Streptococcus pyogenes but has also shown some effect on AOM and oral malodor (reviewed in reference 80); (ii) *S. salivarius* M18, which targets dental and gingival health (30); and (iii) *S. salivarius* 24SMB, which targets rAOM and other recurrent URT infections (31) and has recently also been shown to reduce adenoid hypertrophy and OME when used in conjunction with Streptococcus oralis 89a (41). Thus, the literature also points to the potential for *S. salivarius* as a probiotic, but not many probiotic strains have yet been described, indicating the importance of further characterization and screening.

One of the most important ways in which probiotics can benefit their host is by limiting the growth or the establishment of pathogens. Therefore, we characterized the capacity of seven *S. salivarius* isolates to inhibit the growth of OME pathogens in more detail. They inhibited the growth of all tested pathobionts in the direct interaction assay, but not when using filter-sterilized supernatant of an overnight culture (data not shown). This points toward the importance of close interaction between the potential beneficial bacteria and pathobionts in a living state (81), growth-phase-specific gene expression (82), or the requirement of a high culture density to activate the synthesis of antimicrobial molecules (83), as occurs on solid media. However, it is also possible that *S. salivarius* might need a different experimental setup to produce antimicrobial molecules in a broth culture. Genome analysis showed that each isolate harbored multiple secondary metabolites that were potentially involved in the observed inhibition (Table 3), but their activity and target(s) still need to be confirmed through peptide mass spectrometry analysis or *in vitro* knockout studies.

We then evaluated adhesion capacity to the respiratory epithelial cell line Calu-3. These immortalized cells were chosen over primary cells to increase reproducibility. All tested isolates showed an adherence percentage above 1%, in agreement with the fact that they express adhesion factors, with AMBR158 being the best-performing strain. Screening the genomes for adhesion-related genes did not provide a clear explanation for the range of adhesion ability observed for the five *S. salivarius* isolates, indicating that more research into the interaction of *S. salivarius* with human respiratory epithelium is required.

Probiotics “confer,” by definition, “a health benefit to the host” (21) and should thus not be able to cause disease or contribute to the survival of pathogens, e.g., by spreading antibiotic resistance genes. Our isolates did not harbor any known virulence genes. However, it must be stated that genotyping for virulence factors is challenging and limited because nonpathogenic species possess “virulence” genes in common with pathogenic ones. In our isolates, we rationalized that the two genes flagged by VFDB could be considered adaptation factors, used for good function in the URT. Indeed, sometimes these “virulence factors” could be useful to combat a pathogenic species. Two isolates, AMBR055 and AMBR047, on the other hand, harbored acquired genes for macrolide efflux pumps, and AMBR047 additionally showed decreased sensitivity to chloramphenicol *in vitro*, making these isolates unsuitable as probiotic candidates. As an additional safety check, it must be confirmed in future work that the selected *S. salivarius* strains do not have a disruptive effect on the (beneficial members) of the URT microbiome, given their inherent antimicrobial capacity.

We acknowledge that this study has some potential drawbacks. First, in this study, bacteria with a potentially beneficial effect on middle ear health were identified at the nasopharynx level, as healthy middle ear samples did not yield trustworthy microbiome results. The nasopharynx is the natural habitat of the classic middle ear pathobionts, making it a suitable and accessible location for probiotics targeting middle ear health. In addition, our study cannot answer conclusively if ear canal commensals are involved in OME pathogenesis, as it is not possible to completely exclude touching the ear canal or ear drum when sampling middle ear effusion. However, our *S. salivarius* isolates were able to also inhibit the growth of these taxa (*A. otitis* and C. otitidis). Finally, we used samples from two control groups with different extraction methods to make up for the unexpectedly low number of suitable cochlear implant controls, but care was taken during the data analysis to ensure that the observed microbiome differences between health and OME were not due to differences in sample processing.

### Conclusions.

Our study confirms findings from previous work undertaken in the URT area with a robust sequencing and molecular approach. We identified H. influenzae as the most frequent classic middle ear pathobiont for chronic OME in children resident in Flanders, Belgium, with ear canal taxa, especially *A. otitis*, as potential additional etiologic agents. At the nasopharynx level, we identified A. lwoffii and *salivarius* group streptococci as health associated. Phenotypic tests confirmed that five of our *S. salivarius* isolates could inhibit typical respiratory and middle ear pathogens while lacking known virulence and antibiotic resistance mechanisms. Our findings will now have to be confirmed in more elaborate safety and probiotic efficacy studies in humans.

## MATERIALS AND METHODS

More details are provided in Text S1 in the supplemental material.

10.1128/mSystems.00056-21.1 TEXT S1 Supplemental Materials and Methods. Download Text S1, DOCX file, 0.06 MB. Copyright © 2021 Jörissen et al. 2021 Jörissen et al. https://creativecommons.org/licenses/by/4.0/ This content is distributed under the terms of the Creative Commons Attribution 4.0 International license.

### Sample collection.

Ethical approval was obtained from the ethical committee of the Antwerp University Hospital (ClinicalTrials.gov identifier NCT03109496, registered 12 April 2017), and informed consent was obtained from a parent or legal guardian before sampling. OME patients (*n* = 70) were recruited from a group of children aged 1 to 10 years who were receiving unilateral or bilateral tympanostomy tubes with or without concurrent adenoidectomy to relieve symptoms of persistent (≥3 months) OME. One control group consisted of cochlear implant recipients aged 1 to 45 years (*n* = 12), and a second control group consisted of children aged 6 to 30 months who were healthy enough to attend day care (originally sampled for the NPcarriage study [84]; *n* = 41). Exclusion criteria were comorbidities affecting the URT anatomy, immune system, or mucociliary system; acute or chronic URT infection; and use of antibiotics or steroids up to 1 week before surgery. Swabs of the anterior nares, nasopharynx, and ear canal, middle ear effusion aspirates, and, in the case of simultaneous adenoidectomy, both tissue and swabs of the adenoids were collected from OME patients. Cochlear implant controls provided anterior nare and nasopharynx swabs and a middle ear wash, while children attending day care provided a nasopharynx swab. The mean age of cases and controls was compared using Student’s *t* test (*ggpubr* version 0.2.5 [85]).

### 16S rRNA amplicon sequencing, quality control, and data analysis.

DNA of samples from cochlear implant controls and OME patients was extracted with the QIAamp PowerFecal DNA kit and quantified with a Qubit 3.0 fluorometer (Thermo Fisher Scientific). NPcarriage study samples were received as NucliSens easyMag (bioMérieux) extracted DNA. All samples were further processed and sequenced on an Illumina MiSeq desktop sequencer (M00984; Illumina) as described previously (19). After sequencing of the V4 region of the 16S rRNA gene, trimming, error correction, chimera removal, and classification of paired reads against the EzBioCloud 16S database version 19.01.2018 (86) were all performed in DADA2 version 1.6.0 (87). This workflow resulted in an ASV table with a single-nucleotide difference resolution. Sequenced extraction and PCR controls served as indicators of background contamination. For contaminant filtering (see Table S2 in the supplemental material) and data visualization, packages included in *tidyverse* 1.2.1 and the *tidyamplicons* package (https://github.com/SWittouck/tidyamplicons) were used. ASVs of interest were further classified using the online EzBioCloud 16S-based ID web application (update 2020.05.13) (86).

The differential abundance of ASVs between the nasopharynx of OME patients and of controls was calculated using the Analysis of Composition of Microbiomes (ANCOM) R tool (version 1.1.2 [42] with default tuning parameters and stringent correction for multiple testing). Permutational multivariate analysis of variance (PERMANOVA) (*vegan* version 2.2.5 [88]) was used to determine the effect of metadata on microbiome composition and to compare the same anatomic location between cases and controls.

### Isolation of lactic acid bacteria.

Samples from cochlear implant controls were plated out on three different agar media (see Table S5 in the supplemental material). From each plate, one colony per morphology was selected and identified by Sanger sequencing of the 16S rRNA genes (primers 27F and 1492R) (89, 90).

10.1128/mSystems.00056-21.6 TABLE S5 Cultivation conditions. Download Table S5, DOCX file, 0.02 MB. Copyright © 2021 Jörissen et al. 2021 Jörissen et al. https://creativecommons.org/licenses/by/4.0/ This content is distributed under the terms of the Creative Commons Attribution 4.0 International license.

### Whole-genome sequencing, assembly, and analysis.

Bacterial DNA was extracted for whole-genome sequencing following protocol P3 by Alimolaei and Golchin (91) and sequenced on an Illumina MiSeq platform. The resulting reads were assembled *de novo* with SPAdes-based Shovill (https://github.com/tseemann/shovill), followed by quality control with CheckM (92) and annotation with Prokka (93). Assembled contigs were screened against the ResFinder 3.2 database for the presence of transferable antibiotic resistance genes (94), and against the Virulence Factor Database (VFDB) for virulence factors (95) using ABRicate (https://github.com/tseemann/abricate). Secondary metabolites were identified by antiSMASH 5.0 and BAGEL4. Genes of interest were further characterized using NCBI-BLAST (96). In addition, the genomes were submitted to the Pathosystems Resource Integration Center (PATRIC) Comprehensive Genome Analysis pipeline (65) and to Operon-mapper (66). Annotations from these programs were considered in addition to Prokka annotation when looking for toxin and adhesion genes. Starting from the locus tag or gene annotation of *S. salivarius* JIM877, the presence of genes previously associated with adhesion in *S. salivarius* (68, 69) was confirmed with the PATRIC Compare Region Viewer function set to a region size of 100,000 bp, 50 public and private genomes, and using the PLfams method. As PATRIC does not allow setting of which genomes are used for this alignment, we extracted the visualization and aligned the genomic regions of JIM8777 and our own isolates manually. The Compare Region Viewer was also used to visualize the genomic region upstream and downstream of the *mef*(A) gene in AMBR047 and AMBR055 after screening of these genomes with primers for *mef*(A) or *mef*(E) and *mega* (57) could only confirm presence of the *mega* element for AMBR047.

### Antimicrobial screening.

The ability of isolates to inhibit the growth of URT and middle ear pathobionts was tested (i) by overlaying 48-h 2-μl spots of isolate with pathobiont-containing soft agar (spot assay) and (ii) by inoculating 30 μl spent filter-sterilized culture supernatant into wells punched into pathobiont-containing agar (radial diffusion assay) (97). Hexetidine (0.1%, Hextril; Johnson & Johnson) and Todd Hewitt (TH) broth served as positive and negative controls, respectively. For some repetitions, the pH of the TH broth was reduced to 5. Growth conditions are summarized in Table S5. Inhibition zone sizes were compared to the reference hexetidine using an unpaired Wilcoxon rank sum test (*ggpubr* version 0.2.5 [85]).

### Antibiotic susceptibility assay.

Minimum inhibitory concentrations (MIC) of antibiotics (ampicillin, chloramphenicol, clindamycin, erythromycin, gentamicin, tetracycline, streptomycin, and vancomycin) were determined using a broth microdilution assay with 2-fold serial dilutions between 0.5 μg/ml and 128 μg/ml and evaluation of presence/absence of growth after 24 h of incubation (98). Cutoff values for S. thermophilus were used based on the guidelines of the European Food Safety Authority (EFSA) (55).

### Adherence assay to airway epithelial cells.

The human airway epithelial cell line Calu-3 ATCC HTB-55TM (purchased from ATCC, Molsheim Cedex, France) was cultured in 75 cm^2^ flasks containing 20 ml minimum essential medium (MEM; Life Technologies, Erembodegem, Belgium) supplemented with heat-inactivated fetal bovine serum (Thermo Fisher, Asse, Belgium) and penicillin-streptomycin (100 U/ml; Life Technologies) and maintained in a humidified 5% CO_2_ incubator at 37°C. The culture medium was changed every 3 to 4 days, and the cells were passaged weekly at a 1:2 split ratio using a 0.25% trypsin-EDTA solution (Life Technologies). To test the ability to adhere to human respiratory epithelium, 2 × 10^8^ CFU of bacteria were added for 1 h to fully grown Calu-3 cultures seeded at a density of 3 × 10^5^ cells/cm^2^. The CFU/ml of bacteria added and bacteria retrieved after incubation and washing with phosphate-buffered saline (PBS) were compared, as previously described (99). Adhesion percentages were compared to a reference using an unpaired Wilcoxon rank sum test (*ggpubr* version 0.2.5 [85]).

### Data availability.

The 16S rRNA gene sequencing data generated for this study were deposited in the European Nucleotide Archive under accession number PRJEB33591. The whole-genome sequence data have been added to project PRJEB32716 under assembly numbers GCA_905071825 (isolate AMBR024), GCA_905071875 (AMBR037), GCA_905071915 (AMBR047), GCA_905071845 (AMBR055), GCA_905071895 (AMBR074), GCA_905071855 (AMBR075), and GCA_905071905 (AMBR158).
