# Supplementary material for: Case-Control Microbiome Study of Chronic Otitis Media with Effusion in Children Points at Streptococcus salivarius as a Pathobiont-Inhibiting Species
Source: mSystems. 2021 Apr 20;6(2):e00056-21. doi: 10.1128/mSystems.00056-21 (PMC8546964; doi:10.1128/mSystems.00056-21)
Supplement: TABLE S1 [file msystems.00056-21-st001.docx]

**Table S1:** Number of patients enrolled and samples which could have been collected, were collected and passed quality control (QC) after sequencing. CI - Cochlear Implant recipients; DC - Day Care children.

|  | **Otitis Media with Effusion Patients** | | | | | | | | | | |  | **Controls** | | | | | |  |
| --- | --- | --- | --- | --- | --- | --- | --- | --- | --- | --- | --- | --- | --- | --- | --- | --- | --- | --- | --- |
|  | **Nare** | **Nasopharynx** |  | **Adenoids** | |  | **Middle Ears** | |  | **Ear Canal** | |  | **Nare** |  | **Nasopharynx** | |  | **Middle Ear** | |
|  |  |  |  | **Swabs** | **Tissue** |  | **Samples** | **Patients** |  | **Samples** | **Patients** |  | **CI** |  | **CI** | **DC** |  | **CI** | |
| **Enrolled** | 70 | 70 |  | 32 | 28 |  | 136 | 70 |  | 136 | 70 |  | 12 |  | 12 | 41 |  | 12 | |
| Unilateral |  |  |  |  |  |  | 4 | 4 |  | 3 | 4 |  |  |  |  |  |  |  | |
| Bilateral |  |  |  |  |  |  | 132 | 66 |  | 132 | 66 |  |  |  |  |  |  |  | |
| **Collected** | 69 | 68 |  | 29 | 26 |  | 122 | 65 |  | 135 | 69 |  | 12 |  | 12 | 41 |  | 12 | |
| None |  |  |  |  |  |  |  | 5 |  |  | 1 |  |  |  |  |  |  |  | |
| Unilateral |  |  |  |  |  |  | 8 | 8 |  | 3 | 3 |  |  |  |  |  |  |  | |
| Bilateral |  |  |  |  |  |  | 114 | 57 |  | 132 | 66 |  |  |  |  |  |  |  | |
| **Passed QC** | 66 | 67 |  | 26 | 21 |  | 97 | 59 |  | 102 | 59 |  | 8 |  | 10 | 41 |  | 4 | |
| None |  |  |  |  |  |  |  | 11 |  |  | 11 |  |  |  |  |  |  |  | |
| Unilateral |  |  |  |  |  |  | 21 | 21 |  | 16 | 16 |  |  |  |  |  |  |  | |
| Bilateral |  |  |  |  |  |  | 76 | 38 |  | 86 | 43 |  |  |  |  |  |  |  | |
