# Supplementary material for: Case-Control Microbiome Study of Chronic Otitis Media with Effusion in Children Points at Streptococcus salivarius as a Pathobiont-Inhibiting Species
Source: mSystems. 2021 Apr 20;6(2):e00056-21. doi: 10.1128/mSystems.00056-21 (PMC8546964; doi:10.1128/mSystems.00056-21)
Supplement: TABLE S2 [file msystems.00056-21-st002.pdf]

Table S2: Taxa detected in negative extraction and PCR controls and action taken.

Run	Detected in Control Type	Family	Genus	Species	Sequence	Removed	Removal Step/Reason
2	PCR	Moraxellaceae	Acinetobacter	johnsonii/oryzae	TACAGAGGGTGCGAGCGTTAATCGGATTTACTGGGCGTAAAG<br>CGTGCGTAGGCGGCTTTTTAAGTCGGATGTGAAATCCCTGAGC<br>TTAACTTAGGAATTGCATTCGATACTGGGAAGCTAGAGTATGG<br>GAGAGGATGGTAGAATTCCAGGTGTAGCGGTGAAATGCGTAG<br>AGATCTGGAGGAATACCGATGGCGAAGGCAGCCATCTGGCCT<br>AATACTGACGCTGAGGTACGAAAGCATGGGGAGCAAACAGG	FALSE	
3	PCR	Moraxellaceae	Acinetobacter	NA	TACAGAGGGTGCGAGCGTTAATCGGATTTACTGGGCGTAAAG<br>CGTGCGTAGGCGGCTTTTTAAGTCGGATGTGAAATCCCCGAGC<br>TTAACTTGGGAATTGCATTCGATACTGGGAAGCTAGAGTATGG<br>GAGAGGATGGTAGAATTCCAGGTGTAGCGGTGAAATGCGTAG<br>AGATCTGGAGGAATACCGATGGCGAAGGCAGCCATCTGGCCT<br>AATACTGACGCTGAGGTACGAAAGCATGGGGAGCAAACAGG	TRUE	PCR contaminant
3	PCR	Moraxellaceae	Acinetobacter	NA	TACAGAGGGTGCAAGCGTTAATCGGATTTACTGGGCGTAAAG<br>CGCGCGTAGGTGGCCAATTAAGTCAAATGTGAAATCCCCGAG<br>CTTAACTTGGGAATTGCATTCGATACTGGTTGGCTAGAGTATG<br>GGAGAGGATGGTAGAATTCCAGGTGTAGCGGTGAAATGCGTA<br>GAGATCTGGAGGAATACCGATGGCGAAGGCAGCCATCTGGCC<br>TAATACTGACACTGAGGTGCGAAAGCATGGGGAGCAAACAGG	FALSE	
1|3	Extraction	Moraxellaceae	Acinetobacter	NA	TACAGAGGGTGCGAGCGTTAATCGGATTTACTGGGCGTAAAG<br>CGTGCGTAGGCGGCTTCTTAAGTCGGATGTGAAATCCCTGAGC<br>TTAACTTAGGAATTGCATTCGATACTGGGAAGCTAGAGTATGG<br>GAGAGGATGGTAGAATTCCAGGTGTAGCGGTGAAATGCGTAG<br>AGATCTGGAGGAATACCGATGGCGAAGGCAGCCATCTGGCCT<br>AATACTGACGCTGAGGTACGAAAGCATGGGGAGCAAACAGG	FALSE	
2|3	Extraction | Nested PCR	Moraxellaceae	Acinetobacter	NA	TACAGAGGGTGCGAGCGTTAATCGGATTTACTGGGCGTAAAG<br>CGTGCGTAGGCGGCTGATTAAGTCGGATGTGAAATCCCTGAG<br>CTTAACTTAGGAATTGCATTCGATACTGGTCAGCTAGAGTATG<br>GGAGAGGATGGTAGAATTCCAGGTGTAGCGGTGAAATGCGTA<br>GAGATCTGGAGGAATACCGATGGCGAAGGCAGCCATCTGGCC<br>TAATACTGACGCTGAGGTACGAAAGCATGGGGAGCAAACAGG	FALSE	
1|2	Extraction	Pasteurellaceae	Actinobacillus	NA	TACGGAGGGTGCGAGCGTTAATCGGAATAACTGGGCGTAAAG<br>GGCACGCAGGCGGTGACTTAAGTGAGGTGTGAAAGCCCCGGG<br>CTTAACCTGGGAATTGCATTTCATACTGGGTCGCTAGAGTACTT<br>TAGGGAGGGGTAGAATTCCACGTGTAGCGGTGAAATGCGTAG<br>AGATGTGGAGGAATACCGAAGGCGAAGGCAGCCCCTTGGGA<br>ATGTACTGACGCTCATGTGCGAAAGCGTGGGGAGCAAACAGG	FALSE	
2	Extraction | Nested PCR | PCR	Micromonosporaceae	Actinoplanes	tereljensis	GACGTAGGGCGCGAGCGTTGTCCGGATTTATTGGGCGTAAAG<br>AGCTCGTAGGCGGCTTGTCGCGTCGTTCGTGAAAACCTGGGG<br>CTCAACCCCAGGCTTGCGGGCGATACGGGCAGGCTAGAGTTC<br>GGTAGGGGAGACTGGAATTCCTGGTGTAGCGGTGAAATGCGC<br>AGATATCAGGAGGAACACCGGTGGCGAAGGCGGGTCTCTGG<br>GCCGATACTGACGCTGAGGAGCGAAAGCGTGGGGAGCGAAC<br>AGG	TRUE	Run 2-specific contaminant
3	Extraction	Alishewanella_f	Alishewanella	aestuarii/agri/jeotgali	TACGGAGGGTGCAAGCGTTAATCGGAATTACTGGGCGTAAAG<br>CGCACGCAGGCGGCTTTTTAAGTCGGATGTGAAAGCCCCGGG<br>CTCAACCTGGGAATTGCATCTGATACTGGGAAGCTAGAGTATG<br>TGAGAGGGGGGTAGAATTCCAAGTGTAGCGGTGAAATGCGTA<br>GAGATTTGGAGGAATACCAGTGGCGAAGGCGGCCCCCTGGCA<br>CAATACTGACGCTCAGGTGCGAAAGCGTGGGGAGCAAACAGG	TRUE	Run 3-specific contaminant
3	PCR	Rikenellaceae	Alistipes	NA	TACGGAGGATCCAAGCGTTATCCGGATTTATTGGGTTTAAAGG<br>GTGCGTAGGCGGATTGATAAGTTAGAGGTGAAATGTCCGAGC<br>TCAACTCGGGAACTGCCTCTAATACTGTTGATCTAGAGAGTAG<br>ATGCGGTAGGCGGAATGTATGGTGTAGCGGTGAAATGCTTAG<br>AGATCATACAGAACACCGATTGCGAAGGCAGCTTACCAATCTA<br>TATCTGACGTTGAGGCACGAAAGCGTGGGGAGCAAACAGG	TRUE	Run 3-specific contaminant

Run	Detected in Control Type	Family	Genus	Species	Sequence	Removed	Removal Step/Reason
1|2|3	Extraction | Nested PCR | PCR	Carnobacteriaceae	Alloiococcus	otitis	TACGTAGGTGACAAGCGTTGTCCGGATTTATTGGGCGTAAAGCGAGCGCAGGCGGTCCGGTAAGTCTGATGTGAAAGCCCACGGCTCAACCGTGGAACGGCATTGGAAACTGGCGGACTTGAATGTAGCAGAGGAAAGTGGAATTCCATGTGTAGCGGTGGAATGCGTAGATATATGGAGGAACACCAGTGGCGAAAGCGACTTTCTGGGCTATGATTGACGCTGAGGCTCGAAAGCGTGGGGAGCGAACAGG	FALSE	
1	Extraction | PCR	Prevotellaceae	Alloprevotella	NA	TACGGAAGGTCCGGGCGTTATCCGGAATTATTGGGTTTAAAGGGAGCGCAGGCGGGAGTGTAAGTCAGCTGTTAAATATCAGGGCCCAACTCTGTTATGCAGTTGAAACTATATTTCTTGAGTACGCACAGGGATGGCGGAATTCAGGGTGTAGCGGTGAAATGCTTAGATATCCTGAAGAACTCCGATCGCGAAGGCAGCCATCCGGAGCGTAACTGACGCTGAGGCTCGAAGGTGCGGGTATCGAACAGG	TRUE	PCR contaminant
3	Nested PCR | PCR	Prevotellaceae	Alloprevotella	NA	TACGGAAGGTCCGGGCGTTATCCGGATTTATTGGGTTTAAAGGGAGCGTAGGCCGGCTTTTAAGTCAGCTGTGAAAGTCCGCGGCTCAACCGTGGAATTGCAGTTGAAACTGGAGGCCTTGAGTGCACACAGGGATGCCGGAATTCATGGTGTAGCGGTGAAATGCTTAGATATCATGAAGAACTCCGATCGCGAAGGCAAGTGTCCGGGGTGCAACTGACGCTGAGGCTCGAAAGTGCGGGTATCAAACAGG	TRUE	Run 3-specific contaminant
2	Extraction	Prevotellaceae	Alloprevotella	NA	TACGGAAGGTCCAGGCGTTATCCGGATTTATTGGGTTTAAAGGGAGCGTAGGCGGATTATTAAGTCAGTGGTGAAAGACGGTGGCTCAACCATCGTTAGCCATTGAAACTGGTAGTCTTGAGTGCAGACAGGGATGCTGGAACTCGTGGTGTAGCGGTGAAATGCTTAGATATCACGATGAACTCCGATCGCGAAGGCAGGTGTCCGGGCTGCAACTGACGCTGAGGCTCGAAAGTGTGGGTATCAAACAGG	FALSE	
2	PCR	Prevotellaceae	Alloprevotella	NA	TACGGAAGGTCCGGGCGTTATCCGGAATTATTGGGTTTAAAGGGAGCGCAGGCGGGAGTATAAGTCAGCTGTTAAATATCAGGGCCCAACTCTGTTATGCAGTTGAAACTATATTTCTTGAGTACGCACAGGGATGGCGGAATTCAGGGTGTAGCGGTGAAATGCTTAGATATCCTGAAGAACTCCGATCGCGAAGGCAGCCATCCGGAGCGTAACTGACGCTGAGGCTCGAAGGTGCGGGTATCGAACAGG	FALSE	
2	Extraction	Erythrobacteraceae	Altererythrobacter	NA	TACGGAGGGAGCTAGCGTTGTTCGGATATACTGGGCGTAAAGCGCACGTAGGCGGCGCGGTAAGTCAGGGGTGAAATCCCGGAGCTCAACTCCGGAACTGCCCTTGAAACTGCAGTGCTAGAATTCTGGAGAGGCGAGTGGAATTCCGAGTGTAGAGGTGAAATTCGTAGATATTCGGAAGAACACCAGTGGCGAAGGCGACTCGCTGGACAGACATTGACGCTGAGGTGCGAAAGCGTGGGGAGCAAACAGG	TRUE	Run 2-specific contaminant
2	Extraction	Bacillaceae	Anoxybacillus	bogrovensis/flavithermus/gonensis/mongoliensis/rupiensis/salavatliensis/suryakundensis/thermarum	TACGTAGGTGGCAAGCGTTGTCCGGAATTATTGGGCGTAAAGCGCGCGCAGGCGGTTCCTTAAGTCTGATGTGAAAGCCCACGGCTCAACCGTGGAGGGTCATTGGAAACTGGGGGACTTGAGTGCAGAAGAGGAGAGCGGAATTCCACGTGTAGCGGTGAAATGCGTAGAGATGTGGAGGAACACCAGTGGCGAAGGCGGCTCTCTGGTCTGTAACTGACGCTGAGGCGCGAAAGCGTGGGGAGCAAACAGG	TRUE	Extraction kit 7 contaminant
2	Extraction | PCR	Iamiaceae	Aquihabitans	NA	CACGTAGGGGGCAAGCGTTGTCCGGATTTATTGGGCGTAAAGAGCTCGTAGGCGGCTGAGTAAGTCGGGTGTTAAATCCCCAGGCTCAACCTGGGACACCACCCGATACTGCTCTAGCTAGAGTCCGGTAGGGGAGCGTGGAATTCCTGGTGTAGCGGTGAAATGCGCAGATATCAGGAGGAACACCAGCGGCGAAGGCGGCGCTCTGGGCCGGAACTGACGCTGAGGAGCGAAAGCGTGGGTAGCAAACAGG	TRUE	Run 2-specific contaminant
3	Nested PCR | PCR	Erysipelotrichaceae	ASTB_g	NA	TACGTAGGTGGCGAGCGTTATCCGGAATGATTGGGCGTAAAGGGTGCGTAGGCGGCCTGCTAAGTCTGGAGTGAAAGGTACCGGCTCAACCGGTACAGGCTCTGGAAACTGGCGGGCTAGAGAGCAGGAGAGGGCGGTGGAACTCCATGTGTAGCGGTAAAATGCGTAGATATATGGAAGAACACCAGTGGCGAAGGCGGCCGCCTGGCCTGTTAACTGACGCTGAGGCACGAAAGCGTGGGGAGCAAATAGG	TRUE	Run 3-specific contaminant

Run	Detected in Control Type	Family	Genus	Species	Sequence	Removed	Removal Step/Reason
2	Extraction	Micrococcaceae	Auritidibacter	ignavus	TACGTAGGGTGCGAGCGTTATCCGGAATTATTGGGCGTAAAGAGCTCGTAGGCGGTTTGTCGCGTCTGCTGTGAAAGCTCAGGGCTTAACTCTGAGTTTGCAGTGGGTACGGGCAGACTGGAGTGCAGTAGGGGAGATTGGAATTCCTGGTGTAGCGGTGGAATGCGCAGATATCAGGAGGAACACCGATGGCGAAGGCAGGTCTCTGGGCTGTTACTGACGCTGAGGAGCGAAAGCATGGGGAGCGAACAGG	FALSE	
2	Extraction	Bacillaceae	Bacillus	NA	TACGTAGGTGGCAAGCGTTGTCCGGATTTATTGGGCGTAAAGCGCGCGCAGGCGGTCCTTTAAGTCTGATGTGAAATCTTGCGGCTCAACCGCAAGCGGTCATTGGAAACTGGGGGACTTGAGTGCAGAAGAGGAAAGCGGAATTCCACGTGTAGCGGTGAAATGCGTAGAGATGTGGAGGAACACCAGTGGCGAAGGCGGCTTTCTGGTCTGTAACTGACGCTGAGGCGCGAAAGCGTGGGGAGCAAACAGG	TRUE	Extraction kit 7 contaminant
2	Extraction	Bacillaceae	Bacillus_g6	coagulans	TACGTAGGTGGCAAGCGTTGTCCGGAATTATTGGGCGTAAAGCGCGCGCAGGCGGCTTCTTAAGTCTGATGTGAAATCTTGCGGCTCAACCGCAAGCGGTCATTGGAAACTGGGAGGCTTGAGTGCAGAAGAGGAGAGTGGAATTCCACGTGTAGCGGTGAAATGCGTAGAGATGTGGAGGAACACCAGTGGCGAAGGCGGCTCTCTGGTCTGTAACTGACGCTGAGGCGCGAAAGCGTGGGGAGCAAACAGG	FALSE	
3	PCR	Bacteroidaceae	Bacteroides	NA	TACGGAGGATCCGAGCGTTATCCGGATTTATTGGGTTTAAAGGGAGCGTAGGCGGGTTGTTAAGTCAGTTGTGAAAGTTTGCGGCTCAACCGTAAAATTGCAGTTGATACTGGCATCCTTGAGTACAGTAGAGGTAGGCGGAATTCGTGGTGTAGCGGTGAAATGCTTAGATATCACGAAGAACTCCGATTGCGAAGGCAGCCTGCTGGACTGTAACTGACGCTGATGCTCGAAAGTGTGGGTATCAAACAGG	TRUE	Run 3-specific contaminant
3	Extraction	Bifidobacteriaceae	Bifidobacterium	adolescentis/faecale/stercoris	TACGTAGGGTGCAAGCGTTATCCGGAATTATTGGGCGTAAAGGGCTCGTAGGCGGTTCGTCGCGTCCGGTGTGAAAGTCCATCGCTTAACGGTGGATCCGCGCCGGGTACGGGCGGGCTTGAGTGCGGTAGGGGAGACTGGAATTCCCGGTGTAACGGTGGAATGTGTAGATATCGGGAAGAACACCAATGGCGAAGGCAGGTCTCTGGGCCGTCACTGACGCTGAGGAGCGAAAGCGTGGGGAGCGAACAGG	TRUE	Extraction kit 10 contaminant
2|3	Extraction | Nested PCR | PCR	Bifidobacteriaceae	Bifidobacterium	animalis/thermacidophilum/thermophilum	TACGTAGGGTGCGAGCGTTATCCGGATTTATTGGGCGTAAAGGGCTCGTAGGCGGTTCGTCGCGTCCGGTGTGAAAGTCCATCGCCTAACGGTGGATCTGCGCCGGGTACGGGCGGGCTGGAGTGCGGTAGGGGAGACTGGAATTCCCGGTGTAACGGTGGAATGTGTAGATATCGGGAAGAACACCAATGGCGAAGGCAGGTCTCTGGGCCGTTACTGACGCTGAGGAGCGAAAGCGTGGGGAGCGAACAGG	TRUE	PCR contaminant
2	Extraction	Bifidobacteriaceae	Bifidobacterium	breve/longum	TACGTAGGGTGCAAGCGTTATCCGGAATTATTGGGCGTAAAGGGCTCGTAGGCGGTTCGTCGCGTCCGGTGTGAAAGTCCATCGCTTAACGGTGGATCCGCGCCGGGTACGGGCGGGCTTGAGTGCGGTAGGGGAGACTGGAATTCCCGGTGTAACGGTGGAATGTGTAGATATCGGGAAGAACACCAATGGCGAAGGCAGGTCTCTGGGCCGTTACTGACGCTGAGGAGCGAAAGCGTGGGGAGCGAACAGG	FALSE	
1	PCR	Dermabacteraceae	Brachybacterium	NA	TACGTAGGGCGCAAGCGTTGTCCGGAATTATTGGGCGTAAAGAGCTTGTAGGTGGCTTGTCGCGTCTGCCGTGAAAACCCGAGGCTCAACCTCGGGCGTGCGGTGGGTACGGGCAGGCTAGAGTGTGGTAGGGGAGACTGGAACTCCTGGTGTAGCGGTGATATGCGCAGATATCAGGAAGAACACCGATGGCGAAGGCAGGTCTCTGGGCCATTACTGACACTGAGAAGCGAAAGCATGGGGAGCGAACAGG	TRUE	Run 1-specific contaminant
2	Extraction | PCR	Bradyrhizobiaceae	Bradyrhizobium	NA	TACGAAGGGGGCTAGCGTTGCTCGGAATCACTGGGCGTAAAGGGTGCGTAGGCGGGTCTTTAAGTCAGGGGTGAAATCCTGGAGCTCAACTCCAGAACTGCCTTTGATACTGAAGATCTTGAGTTCGGGAGAGGTGAGTGGAACTGCGAGTGTAGAGGTGAAATTCGTAGATATTCGCAAGAACACCAGTGGCGAAGGCGGCTCACTGGCCCGATACTGACGCTGAGGCACGAAAGCGTGGGGAGCAAACAGG	TRUE	PCR contaminant

Run	Detected in Control Type	Family	Genus	Species	Sequence	Removed	Removal Step/Reason
2	Extraction	Caulobacteraceae	Brevundimonas	alba/denitrificans	TACGAAGGGGGCTAGCGTTGCTCGGAATTACTGGGCGTAAAGGGAGCGTAGGCGGACATTTAAGTCAGAGGTGAAATCCCGGAGCTTAACTTCGGAACTGCCTTTGATACTGGGTGTCTTGAGTGTGAGAGAGGTATGTGGAACTCCGAGTGTAGAGGTGAAATTCGTAGATATTCGGAAGAACACCAGTGGCGAAGGCGACATACTGGCTCATTACTGACGCTGAGGCTCGAAAGCGTGGGGAGCAAACAGG	FALSE	
3	Extraction	Caulobacteraceae	Brevundimonas	albigilva/nasdae/vesicularis	TACGAAGGGGGCTAGCGTTGCTCGGAATTACTGGGCGTAAAGGGAGCGTAGGCGGACATTTAAGTCAGGGGTGAAATCCCGGGGCTCAACCTCGGAATTGCCTTTGATACTGGGTGTCTTGAGTATGAGAGAGGTGTGTGGAACTCCGAGTGTAGAGGTGAAATTCGTAGATATTCGGAAGAACACCAGTGGCGAAGGCGACACACTGGCTCATTACTGACGCTGAGGCTCGAAAGCGTGGGGAGCAAACAGG	TRUE	Extraction kit 9 contaminant
2|3	Extraction | PCR	Burkholderiaceae	Burkholderia	arboris/contaminans/glumae/lata/mallei/metallica/pseudomallei/pyrrocinia/stagnalis/thailandensis/ubonensis	TACGTAGGGTGCGAGCGTTAATCGGAATTACTGGGCGTAAAGCGTGCGCAGGCGGTTTGCTAAGACCGATGTGAAATCCCCGGGCTCAACCTGGGAACTGCATTGGTGACTGGCAGGCTAGAGTATGGCAGAGGGGGGTAGAATTCCACGTGTAGCAGTGAAATGCGTAGAGATGTGGAGGAATACCGATGGCGAAGGCAGCCCCCTGGGCCAATACTGACGCTCATGCACGAAAGCGTGGGGAGCAAACAGG	TRUE	PCR contaminant
1	Extraction	Campylobacteraceae	Campylobacter	NA	TACGGAGGGTGCAAGCGTTACTCGGAATCACTGGGCGTAAAGGACGCGTAGGCGGATTATCAAGTCTCTTGTGAAATCCTATGGCTTAACCATAGAACTGCTTGGGAAACTGATAATCTAGAGTGAGGGAGAGGCAGATGGAATTGGTGGTGTAGGGGTAAAATCCGTAGAGATCACCAGGAATACCCATTGCGAAGGCGATCTGCTGGAACTCAACTGACGCTAATGCGTGAAAGCGTGGGGAGCAAACAGG	FALSE	
2	PCR	Caulobacteraceae	Caulobacter	fusiformis	TACGAAGGGGGCTAGCGTTGCTCGGAATTACTGGGCGTAAAGGGAGCGTAGGCGGATAGTTCAGTCAGAGGTGAAAGCCCAGGGCTCAACCTTGGAACTGCCTTTGATACTGGCTATCTTGAGTTTGGGAGAGGTATGTGGAACTCCGAGTGTAGAGGTGAAATTCGTAGATATTCGGAAGAACACCAGTGGCGAAGGCGACATACTGGCCCAATACTGACGCTGAGGCTCGAAAGCGTGGGGAGCAAACAGG	TRUE	Run 2-specific contaminant
2	PCR	Caulobacteraceae	Caulobacter	NA	TACGAAGGGGGCTAGCGTTGCTCGGAATTACTGGGCGTAAAGGGAGCGTAGGCGGATAGTTTAGTCAGAGGTGAAAGCCCAGGGCTCAACCTTGGAATTGCCTTTGATACTGGCTATCTTGAGTATGGAAGAGGTATGTGGAACTCCGAGTGTAGAGGTGAAATTCGTAGATATTCGGAAGAACACCAGTGGCGAAGGCGACATACTGGTCCATTACTGACGCTGAGGCTCGAAAGCGTGGGGAGCAAACAGG	TRUE	PCR contaminant
2	Extraction | Nested PCR | PCR	Cellulomonadaceae	Cellulomonas	NA	TACGTAGGGCGCAAGCGTTGTCCGGAATTATTGGGCGTAAAGAGCTCGTAGGCGGTTTGTCGCGTCTGCTGTGAAAACCTAAGGCTCAACCTTGGGCTTGCAGTGGGTACGGGCAGACTAGAGTGCGGTAGGGGTGACTGGAATTCCTGGTGTAGCGGTGGAATGCGCAGATATCAGGAGGAACACCGATGGCGAAGGCAGGTCACTGGGCCGCAACTGACGCTGAGGAGCGAAAGCATGGGGAGCGAACAGG	TRUE	PCR contaminant
3	Extraction	Flavobacteriaceae	Chryseobacterium	hominis	TACGGAGGGTGCAAGCGTTATCCGGATTTATTGGGTTTAAAGGGTCCGTAGGCGGATCTGTAAGTCAGTGGTGAAATCTCACAGCTTAACTGTGAAACTGCCATTGATACTGCAGGTCTTGAGTAAATTTGAAGTGGCTGGAATAAGTAGTGTAGCGGTGAAATGCATAGATATTACTTAGAACACCAATTGCGAAGGCAGGTCACTAAGATTTAACTGACGCTGATGGACGAAAGCGTGGGGAGCGAACAGG	FALSE	
3	Extraction	Micrococcaceae	Citricoccus	NA	TACGTAGGGCGCGAGCGTTATCCGGAATTATTGGGCGTAAAGAGCTCGTAGGCGGTTTGTCGCGTCTGCCGTGAAAGTCCGGGGCTTAACCCCGGATCTGCGGTGGGTACGGGCAGACTAGAGTGCAGTAGGGGAGACTGGAATTCCTGGTGTAGCGGTGAAATGCGCAGATATCAGGAGGAACACCGATGGCGAAGGCAGGTCTCTGGGCTGTAACTGACGCTGAGGAGCGAAAGCATGGGGAGCGAACAGG	TRUE	Extraction kit 11 contaminant

Run	Detected in Control Type	Family	Genus	Species	Sequence	Removed	Removal Step/Reason
2	PCR	Enterobacteriaceae	Citrobacter	*NA*	TACGGAGGGTGCAAGCGTTAATCGGAATTACTGGGCGTAAAGCGCACGCAGGCGGTCTGTCAAGTCGGATGTGAAATCCCCGGGCTCAACCTGGGAACTGCATCCGAAACTGGCAGGCTAGAGTCTTGTAGAGGGGGGTAGAATTCCAGGTGTAGCGGTGAAATGCGTAGAGATCTGGAGGAATACCGGTGGCGAAGGCGGCCCCCTGGACAAAGACTGACGCTCAGGTGCGAAAGCGTGGGGAGCAAACAGG	FALSE	
2	Extraction	Flavobacteriaceae	Cloacibacterium	normanense/rupense	TACGGAGGGTGCAAGCGTTATCCGGATTTATTGGGTTTAAAGGGTCCGTAGGCGGACTTATAAGTCAGTGGTGAAAGCCTGTCGCTTAACGATAGAACTGCCATTGATACTGTAAGTCTTGAGTATATTTGAGGTAGCTGGAATAAGTAGTGTAGCGGTGAAATGCATAGATATTACTTAGAACACCAATTGCGAAGGCAGGTTACCAAGATATAACTGACGCTGAGGGACGAAAGCGTGGGGAGCGAACAGG	FALSE	
3	Extraction	Comamonadaceae	Comamonas	*NA*	TACGTAGGGTGCAAGCGTTAATCGGAATTACTGGGCGTAAAGCGTGCGCAGGCGGTTTTGTAAGACAGAGGTGAAATCCCCGGGCTCAACCTGGGAACTGCCTTTGTGACTGCAAGGCTGGAGTGCGGCAGAGGGGGATGGAATTCCGCGTGTAGCAGTGAAATGCGTAGATATGCGGAGGAACACCGATGGCGAAGGCAATCCCCTGGGCCTGCACTGACGCTCATGCACGAAAGCGTGGGGAGCAAACAGG	FALSE	
1	Extraction	Corynebacteriaceae	Corynebacterium	accolens/macginleyi	TACGTAGGGTGCGAGCGTTGTCCGGAATTACTGGGCGTAAAGGGCTCGTAGGTGGTTTGTCGCGTCGTCTGTGAAATTCTGGGGCTTAACTCCGGGCGTGCAGGCGATACGGGCATAACTTGAGTGCTGTAGGGGTAACTGGAATTCCTGGTGTAGCGGTGAAATGCGCAGATATCAGGAGGAACACCGATGGCGAAGGCAGGTTACTGGGCAGTTACTGACGCTGAGGAGCGAAAGCATGGGTAGCGAACAGG	FALSE	
2	Extraction	Corynebacteriaceae	Corynebacterium	diphtheriae/vitaeruminis	TACGTAGGGTGCGAGCGTTGTCCGGAATTACTGGGCGTAAAGAGCTCGTAGGTGGTTTGTCGCGTCGTCTGTGAAATTCCGGGGCTTAACTTCGGGCGTGCAGGCGATACGGGCATAACTTGAGTGCTGTAGGGGAGACTGGAATTCCTGGTGTAGCGGTGGAATGCGCAGATATCAGGAGGAACACCGATGGCGAAGGCAGGTCTCTGGGCAGTAACTGACGCTGAGGAGCGAAAGCATGGGGAGCGAACAGG	FALSE	
2	Nested PCR	Corynebacteriaceae	Corynebacterium	durum	TACGTAGGGTGCAAGCGTTGTCCGGATTTACTGGGCGTAAAGAGCTCGTAGGTGGTGTGTCGCGTCGTCTGTGAAATTCCGGGGCTTAACTCCGGGCGTGCAGGCGATACGGGCACGACTAGAGTGCTGTAGGGGTAACTGGAATTCCTGGTGTAGCGGTGAAATGCGCAGATATCAGGAGGAACACCGATGGCGAAGGCAGGTTACTGGGCAGTTACTGACGCTGAGGAGCGAAAGCATGGGGAGCGAACAGG	FALSE	
1	PCR	Corynebacteriaceae	Corynebacterium	freiburgense/terpenotabidum/variabile	TACGTAGGGTGCGAGCGTTGTCCGGAATTACTGGGCGTAAAGAGCTCGTAGGTGGTTTGTCGCGTCGTCTGTGAAATTCCGGGGCTTAACTCCGGGCGTGCAGGCGATACGGGCATAACTTGAGTGCTGTAGGGGAGACTGGAATTCCTGGTGTAGCGGTGAAATGCGCAGATATCAGGAGGAACACCGATGGCGAAGGCAGGTCTCTGGGCAGTAACTGACGCTGAGGAGCGAAAGCATGGGTAGCGAACAGG	TRUE	PCR contaminant
3	Extraction	Corynebacteriaceae	Corynebacterium	imitans	TACGTAGGGTGCGAGCGTTGTCCGGAATTACTGGGCGTAAAGAGCTCGTAGGTGGTTTGTCGCGTCGTTTGTGGAATACCGCAGCTTAACTGCGGGGTTGCAGGCGATACGGGCATAACTTGAGTGCTGTAGGGGAGACTGGAATTCCTGGTGTAGCGGTGGAATGCGCAGATATCAGGAGGAACACCGATGGCGAAGGCAGGTCTCTGGGCAGTAACTGACGCTGAGGAGCGAAAGCATGGGGAGCGAACAGG	TRUE	Extraction kit 11 contaminant
2	Extraction	Corynebacteriaceae	Corynebacterium	jeikeium	TACGTAGGGTGCGAGCGTTGTCCGGAATTACTGGGCGTAAAGAGCTCGTAGGTGGTTTGTCGCGTCGTCTGTGAAATCCCGGGGCTTAACTTCGGGCGTGCAGGCGATACGGGCATAACTAGAGTGCTGTAGGGGAGACTGGAATTCCTGGTGTAGCGGTGAAATGCGCAGATATCAGGAGGAACACCGATGGCGAAGGCAGGTCTCTGGGCAGTTACTGACGCTGAGGAGCGAAAGCATGGGTAGCGAACAGG	FALSE	

Run	Detected in Control Type	Family	Genus	Species	Sequence	Removed	Removal Step/Reason
3	PCR	Corynebacteriaceae	Corynebacterium	minutissimum/singulare/spheniscorum	TACGTAGGGTGCGAGCGTTGTCCGGAATTACTGGGCGTAAAGAGCTCGTAGGTGGTTTGTCGCGTCGTCTGTGAAATTCCGGGGCTTAACTCCGGGCGTGCAGGCGATACGGGCATAACTTGAGTGCTGTAGGGGAGACTGGAATTCCTGGTGTAGCGGTGAAATGCGCAGATATCAGGAGGAACACCGATGGCGAAGGCAGGTCTCTGGGCAGTTACTGACGCTGAGGAGCGAAAGCATGGGTAGCGAACAGG	TRUE	PCR contaminant
1	Extraction	Corynebacteriaceae	Corynebacterium	NA	TACGTAGGGTGCGAGCGTTGTCCGGAATTACTGGGCGTAAAGAGCTCGTAGGTGGTCTGTCGCGTCATTTGTGAAAGCCCGGGGCTTAACTCCGGGTTGGCAGGTGATACGGGCATGACTGGAGTACTGTAGGGGAGACTGGAATTCCTGGTGTAGCGGTGAAATGCGCAGATATCAGGAGGAACACCGGTGGCGAAGGCGGGTCTCTGGGCAGTAACTGACGCTGAGGAGCGAAAGCATGGGTAGCGAACAGG	TRUE	Extraction kit 3 contaminant
1|2	Extraction	Corynebacteriaceae	Corynebacterium	NA	TACGTAGGGTGCGAGCGTTGTCCGGAATTACTGGGCGTAAAGAGCTCGTAGGTGGTTTGTCGCGTCGTTTGTGTAAGCCCGCAGCTTAACTGCGGGACTGCAGGCGATACGGGCATAACTTGAGTGCTGTAGGGGAGACTGGAATTCCTGGTGTAGCGGTGGAATGCGCAGATATCAGGAGGAACACCGATGGCGAAGGCAGGTCTCTGGGCAGTAACTGACGCTGAGGAGCGAAAGCATGGGTAGCGAACAGG	FALSE	
3	Extraction	CP009312_f	CP009312_g	s	TACGTAGGGTGCGAGCGTTGTCCGGAATTACTGGGCGTAAAGAGCTCGTAGGCGGTTTGTCACGTCGTCTGTGAAATCCTAGGGCTTAACCCTGGACGTGCAGGCGATACGGGCTGACTTGAGTACTACAGGGGAGACTGGAATTTCTGGTGTAGCGGTGGAATGCACAGATATCAGGAAGAACACCGATGGCGAAGGCAGGTCTCTGGGTAGTAACTGACGCTGAGGAGCGAAAGCATGGGTAGCGAACAGG	FALSE	
1	Extraction	Dermacoccaceae	Dermacoccus	NA	TACGTAGGGTGCGAGCGTTGTCCGGAATTATTGGGCGTAAAGAGCTTGTAGGCGGTTTGTCGCGTCTGCTGTGAAAGACCGGGGCTTAACTCCGGTTCTGCAGTGGGTACGGGCAGACTAGAGTGTGGTAGGGGAGACTGGAATTCCTGGTGTAGCGGTGAAATGCGCAGATATCAGGAGGAACACCGATGGCGAAGGCAGGTCTCTGGGCCATTACTGACGCTGAGAAGCGAAAGCATGGGGAGCGAACAGG	TRUE	Run 1-specific contaminant
1|2|3	Extraction | Nested PCR | PCR	Moraxellaceae	Enhydrobacter	NA	TACAGAGGGTGCGAGCGTTAATCGGAATTACTGGGCGTAAAGCGAGTGTAGGTGGCTCATTAAGTCACATGTGAAATCCCCGGGCTTAACCTGGGAACTGCATGTGATACTGGTGGTGCTAGAATATGTGAGAGGGAAGTAGAATTCCAGGTGTAGCGGTGAAATGCGTAGAGATCTGGAGGAATACCGATGGCGAAGGCAGCTTCCTGGCATAATATTGACACTGAGATTCGAAAGCGTGGGTAGCAAACAGG	TRUE	PCR contaminant
2	Extraction | PCR	Enterobacteriaceae	Enterobacter	NA	TACGGAGGGTGCAAGCGTTAATCGGAATTACTGGGCGTAAAGCGCACGCAGGCGGTCTGTCAAGTCGGATGTGAAATCCCCGGGCTCAACCTGGGAACTGCATTCGAAACTGGCAGGCTAGAGTCTTGTAGAGGGGGGTAGAATTCCAGGTGTAGCGGTGAAATGCGTAGAGATCTGGAGGAATACCGGTGGCGAAGGCGGCCCCCTGGACAAAGACTGACGCTCAGGTGCGAAAGCGTGGGGAGCAAACAGG	TRUE	PCR contaminant
2	Extraction | Nested PCR	Enterobacteriaceae	Enterobacter	NA	TACGGAGGGTGCAAGCGTTAATCGGAATTACTGGGCGTAAAGCGCACGCAGGCGGTCTGTCAAGTCGGATGTGAAATCCCCGGGCTCAACCTGGGAACTGCATTCGAAACTGGCAGGCTGGAGTCTTGTAGAGGGGGGTAGAATTCCAGGTGTAGCGGTGAAATGCGTAGAGATCTGGAGGAATACCGGTGGCGAAGGCGGCCCCCTGGACAAAGACTGACGCTCAGGTGCGAAAGCGTGGGGAGCAAACAGG	FALSE	
3	Extraction	Enterococcaceae	Enterococcus	gallinarum/saccharolyticus	TACGTAGGTGGCAAGCGTTGTCCGGATTTATTGGGCGTAAAGCGAGCGCAGGCGGTTTCTTAAGTCTGATGTGAAAGCCCCCGGCTCAACCGGGGAGGGTCATTGGAAACTGGGAGACTTGAGTGCAGAAGAGGAGAGTGGAATTCCATGTGTAGCGGTGAAATGCGTAGATATATGGAGGAACACCAGTGGCGAAGGCGGCTCTCTGGTCTGTAACTGACGCTGAGGCTCGAAAGCGTGGGGAGCGAACAGG	TRUE	Extraction kit 10 contaminant

Run	Detected in Control Type	Family	Genus	Species	Sequence	Removed	Removal Step/Reason
2	Extraction	Enterococcaceae	Enterococcus	NA	TACGTAGGTGGCAAGCGTTGTCCGGATTTATTGGGCGTAAAGCGAGCGCAGGCGGTCTTTTAAGTCTGATGTGAAAGCCCCCGGCTTAACCGGGGAGGGTCATTGGAAACTGGGAGACTTGAGTGCAGAAGAGGAAAGCGGAATTCCATGTGTAGCGGTGAAATGCGTAGATATATGGAGGAACACCAGTGGCGAAGGCGGCTTTCTGGTCTGTAACTGACGCTGAGGCTCGAAAGCGTGGGGAGCAAACAGG	TRUE	Extraction kit 7 contaminant
2	Extraction	Enterobacteriaceae	Escherichia	NA	TACGGAGGGTGCAAGCGTTAATCGGAATTACTGGGCGTAAAGCGCACGCAGGCGGTTTGTTAAGTCAGATGTGAAATCCCCGGGCTCAACCTGGGAACTGCATCTGATACTGGCAAGCTTGAGTCTCGTAGAGGGGGGTAGAATTCCAGGTGTAGCGGTGAAATGCGTAGAGATCTGGAGGAATACCGGTGGCGAAGGCGGCCCCCTGGACGAAGACTGACGCTCAGGTGCGAAAGCGTGGGGAGCAAACAGG	FALSE	
1	Extraction	Peptoniphilaceae	Ezakiella	NA	TACGTATGGGGCGAGCGTTGTCCGGAATTATTGGGCGTAAAGGGTACGCAGGCGGTTTACCAAGTTGGATGTGAAATCTTGTAGCTCAACTACAAACGTGCATCCAAAACTGGTTAACTTGAGTTAAGGAGAGGTAAGTGGAATTCCTGGTGTAGCGGTGGAATGCGTAGATATCAGGAGGAATACCGGTGGCGAAGGCGACTTACTGGACTTAAACTGACGCTGAGGTACGAAAGCGTGGGGAGCAAACAGG	FALSE	
2	Extraction	Ruminococcaceae	Faecalibacterium	NA	AACGTAGGTCACGAGCGTTGTCCGGAATTACTGGGTGTAAAGGGAGCGCAGGCGGGTATGCAAGTTGGGAGTGAAATACATGGGCTCAACCCATGAACTGCTCTCAAAACTGTGTATCTTGAGTAGTGCAGAGGTAGGCGGAATTCCCGGTGTAGCGGTGGAATGCGTAGATATCGGGAGGAACACCAGTGGCGAAGGCGGCCTACTGGGCACCAACTGACGCTGAGGCTCGAAAGTGTGGGTAGCAAACAGG	TRUE	Extraction kit 7 contaminant
3	PCR	Erysipelotrichaceae	Faecalibaculum	NA	TACGTAGGTGGCGAGCGTTATCCGGAATGATTGGGCGTAAAGGGTGCGCAGGCGGTCCTGCAAGTCTGGAGTGAAACGTATGAGCTCAACTCATGCATGGCTCTGGAAACTGGAGGACTGGAGAGCAGGAGAGGGCGGTGGAACTCCATGTGTAGCGGTAAAATGCGTAGATATATGGAAGAACACCAGTGGCGAAGGCGGCCGCCTGGCCTGTTGCTGACGCTGAGGCACGAAAGCGTGGGGAGCAAATAGG	TRUE	Run 3-specific contaminant
2	Extraction	Ruminococcaceae	Fastidiosipila	NA	TACGTAGGTGGCGAGCGTTATCCGGAATTACTGGGTGTAAAGGGCGTGTAGGCGGCACTGTAAGTCAGATGTGAAATCTCCCGGCTCAACCGGGAGCGTGCATCTGATACTGCAGTACTTGAGTGATAGAGGGGAAAGCGGAATTCCTAGTGTAGCGGTAAAATGCGTAGATATTAGGAGGAACACCAGTGGCGAAGGCGGCTTTCTGGCTATTAACTGACGCTGAGGCGCGAAAGCGTGGGGAGCAAACAGG	TRUE	Run 2-specific contaminant
1	Extraction	Peptoniphilaceae	Fenollaria	NA	TACGTAAGGGGCGAGCGTTGTCCGGAATTATTGGGCGTAAAGAGTGCGTAGGCGGCAAATTAAGTCAGATGTGAAAACTAAGGGCTCAACCCATAGATTGCATCTGAAACTGATATGCTTGAGTCAAGGAGAGGAAAGTGGAATTCCTAGTGTAGCGGTGGAATGCGTAGATATTAGGAGGAATACCGGTGGCGAAGGCGACTTTCTGGACTTGTACTGACGCTGAGGCACGAAAGCGTGGGGAGCAAACAGG	FALSE	
2	Extraction	Peptoniphilaceae	Finegoldia	magna	TACGTATGGAGCGAGCGTTGTCCGGAATTATTGGGCGTAAAGGGTACGCAGGCGGTTTAATAAGTCGAATGTTAAAGATCGGGGCTCAACCCCGTAAAGCATTGGAAACTGATAAACTTGAGTAGTGGAGAGGAAAGTGGAATTCCTAGTGTAGTGGTGAAATACGTAGATATTAGGAGGAATACCAGTAGCGAAGGCGACTTTCTGGACACAAACTGACGCTGAGGTACGAAAGCGTGGGGAGCAAACAGG	FALSE	
1	Extraction	Flavobacteriaceae	Flavobacterium	fluvii/myungsuense/yonginense	TACGGAGGATCCAAGCGTTATCCGGAATCATTGGGTTTAAAGGGTCCGTAGGCGGTCTTATAAGTCAGTGGTGAAATCTCCCCGCTCAACGGGGAAACGGCCATTGATACTGTAGGACTTGAATTATTAGGAAGTAACTAGAATATGTAGTGTAGCGGTGAAATGCTTAGAGATTACATGGAATACCAATTGCGAAGGCAGGTTACTACTAATGGATTGACGCTGATGGACGAAAGCGTGGGTAGCGAACAGG	TRUE	Extraction kit 5 contaminant

Run	Detected in Control Type	Family	Genus	Species	Sequence	Removed	Removal Step/Reason
1|3	Extraction	Neisseriaceae	FM873692_g	s	TACGTAGGGTGCAAGCGTTAATCGGAATTATTGGGCGTAAAGCGAGTGCAGACGGTTACTTAAGCCAGATGTGAAATCCCCAAGCTTAACTTGGGACGTGCATTTGGAACTGGGTGACTAGAGTGTGTCAGAGGGAGGTAGAATTCCACATGTAGCGGTGGAATGCGTAGAGATGTGGAGGAATACCGATGGCGAAGGCAGCCTCCTGGGATAACACTGACGTTGAGGCTCGAAAGCGTGGGGAGCAAACAGG	FALSE	
3	PCR	FR888536_f	FR888536_g	NA	TACGGGGGGTGCAAGCGTTGTCCGGAATCATTGGGCGTAAAGAGTTCGTAGGCGGCACGTAAAGTCTGGTGTTAAAGGCAGAGGCTCAACTTCTGTACGGCACTGGATACTTGCGAGCTAGAATGCGGTAGAGGTAAAGGGAATTCCTGGTGTAGCGGTGAAATGCTTAGATATCAGGAGGAACATCGGTGGCGTAAGCGCTTTACTGGGCCGTAATTGACGCTGAGGAACGAAAGCCAGGGTAGCAAATGGG	TRUE	Run 3-specific contaminant
1	Extraction | PCR	Fusobacteriaceae	Fusobacterium	NA	TACGTATGTCACGAGCGTTATCCGGATTTATTGGGCGTAAAGCGCGTCTAGGTGGTTATGTAAGTCTGATGTGAAAATGCAGGGCTCAACTCTGTATTGCGTTGGAAACTGTATAACTAGAGTACTGGAGAGGTAAGCGGAACTACAAGTGTAGAGGTGAAATTCGTAGATATTTGTAGGAATGCCGATGGGGAAGCCAGCTTACTGGACAGATACTGACGCTGAAGCGCGAAAGCGTGGGTAGCAAACAGG	FALSE	
1	Extraction | PCR	Fusobacteriaceae	Fusobacterium	nucleatum	TACGTATGTCACGAGCGTTATCCGGATTTATTGGGCGTAAAGCGCGTCTAGGTGGTTATATAAGTCTGATGTGAAAATGCAGGGCTCAACTCTGTATTGCGTTGGAAACTGTATAACTAGAGTACTGGAGAGGTAAGCGGAACTACAAGTGTAGAGGTGAAATTCGTAGATATTTGTAGGAATGCCGATGGGGAAGCCAGCTTACTGGACAGATACTGACGCTGAAGCGCGAAAGCGTGGGTAGCAAACAGG	FALSE	
1|2	Extraction | Nested PCR	Gemella_f	Gemella	NA	TACGTAGGTGGCAAGCGTTGTCCGGAATTATTGGGCGTAAAGCGCGCGCAGGTGGTTTAATAAGTCTGATGTGAAAGCCCACGGCTCAACCGTGGAGGGTCATTGGAAACTGTTAAACTTGAGTGCAGGAGAGAAAAGTGGAATTCCTAGTGTAGCGGTGAAATGCGTAGAGATTAGGAGGAACACCAGTGGCGAAGGCGGCTTTTTGGCCTGTAACTGACACTGAGGCGCGAAAGCGTGGGGAGCAAACAGG	FALSE	
2	Extraction	Bacillaceae	Geobacillus	stearothermophilus	TACGTAGGGGGCGAGCGTTGTCCGGAATTATTGGGCGTAAAGCGCGCGCAGGCGGTCTCTTAAGTCTGATGTGAAAGCCCACGGCTCAACCGTGGAGGGTCATTGGAAACTGGGGGACTTGAGGGCAGGAGAGGAGAGCGGAATTCCACGTGTAGCGGTGAAATGCGTAGAGATGTGGAGGAACACCAGTGGCGAAGGCGGCTCTCTGGCCTGCACCTGACGCTGAGGCGCGAAAGCGTGGGGAGCAAACAGG	TRUE	Extraction kit 7 contaminant
1|3	Extraction	Nocardiaceae	Gordonia	terrae	TACGTAGGGTGCGAGCGTTGTCCGGAATTACTGGGCGTAAAGAGCTCGTAGGCGGTTTGTCGCGTCGTCTGTGAAATTCTGCAACTCAATTGCAGGCGTGCAGGCGATACGGGCAGACTTGAGTACTACAGGGGAGACTGGAATTCCTGGTGTAGCGGTGAAATGCGCAGATATCAGGAGGAACACCGGTGGCGAAGGCGGGTCTCTGGGTAGTAACTGACGCTGAGGAGCGAAAGCGTGGGTAGCGAACAGG	TRUE	Extraction kit 2 contaminant
1|2	Extraction	Pasteurellaceae	Haemophilus	aegyptius	TACGGAGGGTGCGAGCGTTAATCGGAATAACTGGGCGTAAAGGGCACGCAGGCGGTTATTTAAGTGAGGTGTGAAAGCCCTGGGCTTAACCTAGGAATTGCATTTCAGACTGGGTAACTAGAGTACTTTAGGGAGGGGTAGAATTCCACGTGTAGCGGTGAAATGCGTAGAGATGTGGAGGAATACCGAAGGCGAAGGCAGCCCCTTGGGAATGTACTGACGCTCATGTGCGAAAGCGTGGGGAGCAAACAGG	FALSE	
1|2|3	Extraction | PCR	Pasteurellaceae	Haemophilus	NA	TACGGAGGGTGCGAGCGTTAATCGGAATAACTGGGCGTAAAGGGCACGCAGGCGGTTATTTAAGTGAGGTGTGAAAGCCCCGGGCTTAACCTGGGAATTGCATTTCAGACTGGGTAACTAGAGTACTTTAGGGAGGGGTAGAATTCCACGTGTAGCGGTGAAATGCGTAGAGATGTGGAGGAATACCGAAGGCGAAGGCAGCCCCTTGGGAATGTACTGACGCTCATGTGCGAAAGCGTGGGGAGCAAACAGG	FALSE	

Run	Detected in Control Type	Family	Genus	Species	Sequence	Removed	Removal Step/Reason
1|2	Extraction	Pasteurellaceae	Haemophilus	quentini	TACGGAGGGTGCGAGCGTTAATCGGAATAACTGGGCGTAAAGGGCACGCAGGCGGTTATTTAAGTGAGGTGTGAAAGCCCTGGGCTTAACCTGGGAATTGCATTTCAGACTGGGTAACTAGAGTACTTTAGGGAGGGGTAGAATTCCACGTGTAGCGGTGAAATGCGTAGAGATGTGGAGGAATACCGAAGGCGAAGGCAGCCCCTTGGGAATGTACTGACGCTCATGTGCGAAAGCGTGGGGAGCAAACAGG	FALSE	
3	PCR	S24-7_f	HM124247_g	NA	TACGGAGGATGCGAGCGTTATCCGGATTTATTGGGTTTAAAGGGTGCGTAGGCGGACGGTCAAGTCAGCGGTAAAAATGCGGTGCTCAACGCCGTACAGCCGTTGAAACTGGCAGTCTTGAGTGGGCGAGAAGTATGCGGAATGCGTGGTGTAGCGGTGAAATGCATAGATATCACGCAGAACTCCGATTGCGAAGGCAGCATACCGGCGCCCGACTGACGCTGAGGCACGAAAGCGTGGGTATCGAACAGG	TRUE	Run 3-specific contaminant
2	PCR	Intrasporangiaceae	Intrasporangium	NA	TACGTAGGGTGCGAGCGTTGTCCGGAATTATTGGGCGTAAAGAGCTTGTAGGCGGTTTGTCGCGTCTGCTGTGAAAATCCGGGGCTCAACCCCGGACTTGCAGTGGGTACGGGCAGACTAGAGTGTGGTAGGGGAGACTGGAATTCCTGGTGTAGCGGTGGAATGCGCAGATATCAGGAGGAACACCGATGGCGAAGGCAGGTCTCTGGGCCACTACTGACGCTGAGAAGCGAAAGCATGGGGAGCGAACAGG	TRUE	Run 2-specific contaminant
2	PCR	FM253648_f	JN178749_g	NA	TACGTAGGTGGCGAACGTTATCCGGAATTACTGGGCGTAAAGGGTCCGCAGGCGGGTCACCAAGTCCGGCGTGAAAGCTCCCGGCTCAACTGGGAGAGGGCGTTGGAAACTGGTGGTCTTGAATCTCAGAGAGGTCGGTGGAATTCCCGGTGTAGTGGTGGAATGCGTAGAGATCGGGAGGAACCTCGAAAGCGAAGGCAACCGGCTGGCTGAAGATTGACGCTGAGGGACGAAAGCGTGGGGAGCGAACAGG	TRUE	Run 2-specific contaminant
2	Nested PCR | PCR	Kineosporiaceae	Kineosporia	mesophila	TACGTAGGGTGCAAGCGTTGTCCGGAATTATTGGGCGTAAAGAGCTCGTAGGCGGTCTGTCGCGTCTGCTGTGAAAACCTAGGGCTTAACTCTGGGCGTGCAGTGGGTACGGGCAGACTAGAGTGCGGTAGGGGAGACTGGAATTCCTGGTGTAGCGGTGAAATGCGCAGATATCAGGAGGAACACCGGTGGCGAAGGCGGGTCTCTGGGCCGTAACTGACGCTGAGGAGCGAAAGCATGGGGAGCGAACAGG	TRUE	Run 2-specific contaminant
2	Extraction | PCR	Kineosporiaceae	Kineosporia	rhamnosa	TACGTAGGGTGCAAGCGTTGTCCGGAATTATTGGGCGTAAAGAGCTCGTAGGCGGTCTGTCGCGTCTGCTGTGAAAACTCAGGGCTTAACCCTGAGCTTGCAGTGGGTACGGGCAGACTAGAGTGCGGTAGGGGAGACTGGAATTCCTGGTGTAGCGGTGAAATGCGCAGATATCAGGAGGAACACCGGTGGCGAAGGCGGGTCTCTGGGCCGTAACTGACGCTGAGGAGCGAAAGCATGGGGAGCGAACAGG	TRUE	Run 2-specific contaminant
1|3	Extraction	Micrococcaceae	Kocuria	arsenatis/rhizophila/salsicia	TACGTAGGGCGCAAGCGTTGTCCGGAATTATTGGGCGTAAAGAGCTCGTAGGCGGTTTGTCGCGTCTGCTGTGAAAGCCCGGGGCTTAACCCCGGGTGTGCAGTGGGTACGGGCAGACTTGAGTGCAGTAGGGGAGACTGGAATTCCTGGTGTAGCGGTGAAATGCGCAGATATCAGGAGGAACACCGATGGCGAAGGCAGGTCTCTGGGCTGTTACTGACGCTGAGGAGCGAAAGCATGGGGAGCGAACAGG	TRUE	Extraction kit 2 contaminant
1|2	Extraction	Lactobacillaceae	Lactobacillus	casei/paracasei/zeae	TACGTAGGTGGCAAGCGTTATCCGGATTTATTGGGCGTAAAGCGAGCGCAGGCGGTTTTTTAAGTCTGATGTGAAAGCCCTCGGCTTAACCGAGGAAGCGCATCGGAAACTGGGAAACTTGAGTGCAGAAGAGGACAGTGGAACTCCATGTGTAGCGGTGAAATGCGTAGATATATGGAAGAACACCAGTGGCGAAGGCGGCTGTCTGGTCTGTAACTGACGCTGAGGCTCGAAAGCATGGGTAGCGAACAGG	FALSE	
2	Extraction	Lactobacillaceae	Lactobacillus	fabifermentans/paraplantarum/pentosus/plantarum	TACGTAGGTGGCAAGCGTTGTCCGGATTTATTGGGCGTAAAGCGAGCGCAGGCGGTTTTTTAAGTCTGATGTGAAAGCCTTCGGCTCAACCGAAGAAGTGCATCGGAAACTGGGAAACTTGAGTGCAGAAGAGGACAGTGGAACTCCATGTGTAGCGGTGAAATGCGTAGATATATGGAAGAACACCAGTGGCGAAGGCGGCTGTCTGGTCTGTAACTGACGCTGAGGCTCGAAAGTATGGGTAGCAAACAGG	FALSE	

Run	Detected in Control Type	Family	Genus	Species	Sequence	Removed	Removal Step/Reason
3	PCR	Lactobacillaceae	Lactobacillus	gasseri/hominis/johnsonii/taiwanensis	TACGTAGGTGGCAAGCGTTGTCCGGATTTATTGGGCGTAAAGC<br>GAGTGCAGGCGGTTCAATAAGTCTGATGTGAAAGCCTTCGGCT<br>CAACCGGAGAATTGCATCAGAAACTGTTGAACTTGAGTGCAG<br>AAGAGGAGAGTGGAACTCCATGTGTAGCGGTGGAATGCGTAG<br>ATATATGGAAGAACACCAGTGGCGAAGGCGGCTCTCTGGTCT<br>GCAACTGACGCTGAGGCTCGAAAGCATGGGTAGCGAACAGG	TRUE	PCR contaminant
1|2	Extraction	Lactobacillaceae	Lactobacillus	mudanjiangensis	TACGTAGGTGGCAAGCGTTGTCCGGATTTATTGGGCGTAAAGC<br>GAGCGCAGGCGGTTTTTTAAGTCTGATGTGAAAGCCTTCGGCT<br>TAACCGGAGAAGTGCATCGGAAACTGGGAAACTTGAGTGCAG<br>AAGAGGACAGTGGAACTCCATGTGTAGCGGTGAAATGCGTAG<br>ATATATGGAAGAACACCAGTGGCGAAGGCGGCTGTCTGGTCT<br>GTAACTGACGCTGAGGCTCGAAAGTATGGGTAGCAAACAGG	TRUE	Extraction kit 4 contaminant
3	Extraction	Lactobacillaceae	Lactobacillus	NA	TACGTAGGTGGCAAGCGTTGTCCGGATTTATTGGGCGTAAAGC<br>GAGTGCAGGCGGTTCGATAAGTCTGATGTGAAAGCCTTCGGC<br>TCAACCGGAGAATTGCATCAGAAACTGTCGAGCTTGAGTACAG<br>AAGAGGAGAGTGGAACTCCATGTGTAGCGGTGAAATGCGTAG<br>ATATATGGAAGAACACCGGTGGCGAAGGCGGCTCTCTGGTCT<br>GTTACTGACGCTGAGGCTCGAAAGCATGGGTAGCGAACAGG	FALSE	
1|3	Extraction	Lactobacillaceae	Lactobacillus	NA	TACGTAGGTGGCAAGCGTTATCCGGATTTATTGGGCGTAAAGC<br>GAGCGCAGGCGGTTTTTTAAGTCTGATGTGAAAGCCCTCGGCT<br>TAACCGAGGAAGTGCATCGGAAACTGGAAAACTTGAGTGCAG<br>AAGAGGACAGTGGAACTCCATGTGTAGCGGTGAAATGCGTAG<br>ATATATGGAAGAACACCAGTGGCGAAGGCGGCTGTCTGGTCT<br>GTAACTGACGCTGAGGCTCGAAAGCATGGGTAGCGAACAGG	FALSE	
2|3	Extraction	Streptococcaceae	Lactococcus	lactis/taiwanensis	TACGTAGGTCCCGAGCGTTGTCCGGATTTATTGGGCGTAAAGC<br>GAGCGCAGGTGGTTTATTAAGTCTGGTGTAAAAGGCAGTGGC<br>TCAACCATTGTATGCATTGGAAACTGGTAGACTTGAGTGCAGG<br>AGAGGAGAGTGGAATTCCATGTGTAGCGGTGAAATGCGTAGA<br>TATATGGAGGAACACCGGTGGCGAAAGCGGCTCTCTGGCCTG<br>TAACTGACACTGAGGCTCGAAAGCGTGGGGAGCAAACAGG	TRUE	Extraction kit 6 contaminant
3	Extraction	Leuconostocaceae	Leuconostoc	citreum/holzapfelii/lactis/palmae	TACGTATGTCCCGAGCGTTATCCGGATTTATTGGGCGTAAAGC<br>GAGCGCAGACGGTTGATTAAGTCTGATGTGAAAGCCCGGAGC<br>TCAACTCCGGAATGGCATTGGAAACTGGTTAACTTGAGTGTTG<br>TAGAGGTAAGTGGAACTCCATGTGTAGCGGTGGAATGCGTAG<br>ATATATGGAAGAACACCAGTGGCGAAGGCGGCTTACTGGACA<br>ACAACTGACGTTGAGGCTCGAAAGTGTGGGTAGCAAACAGG	FALSE	
3	Extraction	Leuconostocaceae	Leuconostoc	mesenteroides	TACGTATGTCCCGAGCGTTATCCGGATTTATTGGGCGTAAAGC<br>GAGCGCAGACGGTTTATTAAGTCTGATGTGAAAGCCCGGAGC<br>TCAACTCCGGAATGGCATTGGAAACTGGTTAACTTGAGTGCAG<br>TAGAGGTAAGTGGAACTCCATGTGTAGCGGTGGAATGCGTAG<br>ATATATGGAAGAACACCAGTGGCGAAGGCGGCTTACTGGACT<br>GCAACTGACGTTGAGGCTCGAAAGTGTGGGTAGCAAACAGG	FALSE	
2	Extraction	Staphylococcaceae	Macrococcus	NA	TACGTAGGTGGCAAGCGTTATCCGGAATTATTGGGCGTAAAG<br>CGCGCGTAGGCGGTCTCTTAAGTCTGATGTGAAAGCCCCCGGC<br>TCAACCGGGGAGGGTCATTGGAAACTGGGAGACTTGAGTACA<br>GAAGAGGAGAGTGGAATTCCATGTGTAGCGGTGAAATGCGCA<br>GAGATATGGAGGAACACCAGTGGCGAAGGCGGCTCTCTGGTC<br>TGTAACTGACGCTGAGGTGCGAAAGCGTGGGGATCAAACAGG	FALSE	
2	Nested PCR	Nocardioidaceae	Marmoricola	NA	TACGTAGGGTGCGAGCGTTGTCCGGAATTATTGGGCGTAAAG<br>GGCTCGTAGGCGGTCTGTTGCGTCAGGAGTGAAAACTCGGGG<br>CTTAACCCCGAGCCTGCTTCTGATACGGGCAGACTAGAGGTAT<br>GCAGGGGAGAACGGAATTCCTGGTGTAGCGGTGAAATGCGCA<br>GATATCAGGAGGAACACCGGTGGCGAAGGCGGTTCTCTGGGC<br>ATTACCTGACGCTGAGGAGCGAAAGTGTGGGGAGCGAACAG<br>G	TRUE	Run 2-specific contaminant

Run	Detected in Control Type	Family	Genus	Species	Sequence	Removed	Removal Step/Reason
2|3	Extraction | Nested PCR | PCR	Methylobacteriaceae	Methylobacterium	NA	TACGAAGGGGGCTAGCGTTGCTCGGAATCACTGGGCGTAAAGGGCGCGTAGGCGGCGTTTTAAGTCGGGGGTGAAAGCCTGTGGCTCAACCACAGAATGGCCTTCGATACTGGGACGCTTGAGTATGGTAGAGGTTGGTGGAACTGCGAGTGTAGAGGTGAAATTCGTAGATATTCGCAAGAACACCGGTGGCGAAGGCGGCCAACTGGACCATTACTGACGCTGAGGCGCGAAAGCGTGGGGAGCAAACAGG	TRUE	PCR contaminant
1	Extraction	Micrococcaceae	Micrococcus	NA	TACGTAGGGTGCGAGCGTTATCCGGAATTATTGGGCGTAAAGAGCTCGTAGGCGGTTTGCCGCGTCTGTCGTGAAAGTCCGGGGCTTAACCCCGGATCTGCGGTGGGTACGGGCAGACTAGAGTGCAGTAGGGGAGACTGGAATTCCTGGTGTAGCGGTGGAATGCGCAGATATCAGGAGGAACACCGATGGCGAAGGCAGGTCTCTGGGCTGTAACTGACGCTGAGGAGCGAAAGCATGGGGAGCGAACAGG	TRUE	Extraction kit 5 contaminant
1|3	Extraction | PCR	Micrococcaceae	Micrococcus	NA	TACGTAGGGTGCGAGCGTTATCCGGAATTATTGGGCGTAAAGAGCTCGTAGGCGGTTTGTCGCGTCTGTCGTGAAAGTCCGGGGCTTAACCCCGGATCTGCGGTGGGTACGGGCAGACTAGAGTGCAGTAGGGGAGACTGGAATTCCTGGTGTAGCGGTGGAATGCGCAGATATCAGGAGGAACACCGATGGCGAAGGCAGGTCTCTGGGCTGTAACTGACGCTGAGGAGCGAAAGCATGGGGAGCGAACAGG	TRUE	PCR contaminant
1|2|3	Extraction | PCR	Moraxellaceae	Moraxella	catarrhalis/nonliquefaciens	TACAGAGGGTGCAAGCGTTAATCGGAATTACTGGGCGTAAAGCGCGCGTAGGTGGTTATTTAAGTCAGATGTGAAAGCCCCGGGCTTAACCTGGGAACTGCATCTGATACTGGATAACTAGAGTAGGTGAGAGGGGAGTAGAATTCCAGGTGTAGCGGTGAAATGCGTAGAGATCTGGAGGAATACCGATGGCGAAGGCAGCTCCCTGGCATCATACTGACACTGAGGTGCGAAAGCGTGGGTAGCAAACAGG	FALSE	
1	PCR	Moraxellaceae	Moraxella	NA	TACAGAGGGTGCAAGCGTTAATCGGAATTACTGGGCGTAAAGCGAGCGTAGGTGGTTTATTAAGTCAGATGTGAAATCCCTGGGCTTAACCTAGGAACTGCATCTGATACTGATAAACTAGAGTAGGTGAGAGGAAAGTAGAATTCCAGGTGTAGCGGTGAAATGCGTAGAGATCTGGAGGAATACCGATGGCGAAGGCAGCTTTCTGGCATCATACTGACACTGAGGTTCGAAAGCGTGGGTAGCAAACAGG	FALSE	
1	Extraction	Erythrobacteraceae	NA	NA	TACGGAGGGAGCTAGCGTTGTTCGGAATTACTGGGCGTAAAGCGCACGTAGGCGGCGATTCAAGTCAGAGGTGAAAGCCCGGGGCTCAACCCCGGAACTGCCTTTGAAACTAGATTGCTAGAATCCTGGAGAGGTGAGTGGAATTCCGAGTGTAGAGGTGAAATTCGTAGATATTCGGAAGAACACCAGTGGCGAAGGCGACTCACTGGACAGGTATTGACGCTGAGGTGCGAAAGCGTGGGGAGCAAACAGG	TRUE	Extraction kit 3 contaminant
1|2	Extraction	Flavobacteriaceae	NA	NA	TACGGAGGGTGCAAGCGTTATCCGGATTTATTGGGTTTAAAGGGTCCGTAGGCGGATTAATAAGTCAGTGGTGAAAGCCCGCAGCTTAACTGTGGAACTGCCATTGATACTGTTAGTCTTGAGTGTAGTTGAAGTGGCTGGAATGAGTAGTGTAGCGGTGAAATGCATAGATATTACTCAGAACACCAATTGCGAAGGCAGGTCACTAAGTTACAACTGACGCTGATGGACGAAAGCGTGGGGAGCGAACAGG	TRUE	Extraction kit 5 contaminant
2	Extraction	Microbacteriaceae	NA	NA	TACGTAGGGCGCAAGCGTTGTCCGGAATTATTGGGCGTAAAGAGCTCGTAGGCGGTCTGTCGCGTCTGCTGTGAAATCCCGAGGCTCAACCTCGGGCTTGCAGTGGGTACGGGCAGACTAGAGTGCGGTAGGGGAGAATGGAATTCCTGGTGTAGCGGTGGAATGCGCAGATATCAGGAGGAACACCGATGGCGAAGGCAGTTCTCTGGGCCGTAACTGACGCTGAGGAGCGAAAGCGTGGGGAGCGAACAGG	FALSE	
1	Extraction	Neisseriaceae	Neisseria	cinerea/mucosa/perflava/subflava	TACGTAGGGTGCGAGCGTTAATCGGAATTACTGGGCGTAAAGCGAGCGCAGACGGTTACTTAAGCAGGATGTGAAATCCCCGGGCTCAACCTGGGAACTGCGTTCTGAACTGGGTGACTAGAGTGTGTCAGAGGGAGGTAGAATTCCACGTGTAGCAGTGAAATGCGTAGAGATGTGGAGGAATACCGATGGCGAAGGCAGCCTCCTGGGATAACACTGACGTTCATGCTCGAAAGCGTGGGTAGCAAACAGG	FALSE	

Run	Detected in Control Type	Family	Genus	Species	Sequence	Removed	Removal Step/Reason
1	Extraction	Neisseriaceae	Neisseria	NA	TACGTAGGGTGCGAGCGTTAATCGGAATTACTGGGCGTAAAGCGGGCGCAGACGGTTACTTAAGCAGGATGTGAAATCCCCGGGCTCAACCTGGGAACTGCGTTCTGAACTGGGTGACTAGAGTGTGTCAGAGGGAGGTAGAATTCCACGTGTAGCAGTGAAATGCGTAGAGATGTGGAGGAATACCGATGGCGAAGGCAGCCTCCTGGGATAACACTGACGTTCATGCCCGAAAGCGTGGGTAGCAAACAGG	FALSE	
2	Nested PCR	Nocardioidaceae	Nocardioides	hwasunensis	TACGTAGGGTGCGAGCGTTGTCCGGAATTATTGGGCGTAAAGGGCTCGTAGGCGGTTTGTCGCGTCGGGAGTGAAAACCAGGTGCTTAACACCTGGCTTGCTTTCGATACGGGCAGACTAGAGGTATTCAGGGGAGAACGGAATTCCTGGTGTAGCGGTGAAATGCGCAGATATCAGGAGGAACACCGGTGGCGAAGGCGGTTCTCTGGGAATGACCTGACGCTGAGGAGCGAAAGTGTGGGGAGCGAACAGG	TRUE	Nested PCR contaminant
2	PCR	Nocardioidaceae	Nocardioides	NA	TACGTAGGGTGCGAGCGTTGTCCGGAATTATTGGGCGTAAAGGGCTCGTAGGCGGTTTGTCGCGTCGGGAGTGAAAACCAGGTGCTTAACACCTGGCTTGCTTTCGATACGGGCAGACTAGAGGTATGCAGGGGAGAACGGAATTCCTGGTGTAGCGGTGAAATGCGCAGATATCAGGAGGAACACCGGTGGCGAAGGCGGTTCTCTGGGCATTACCTGACGCTGAGGAGCGAAAGTGTGGGGAGCGAACAGG	FALSE	
2	Extraction	Nocardioidaceae	Nocardioides	zeicaulis	TACGTAGGGTGCGAGCGTTGTCCGGAATTATTGGGCGTAAAGGGCTCGTAGGCGGTTTGTCGCGTCGGGAGTGAAAACCAGGTGCTTAACACCTGGCTTGCTTTCGATACGGGCAGACTAGAGGTATTCAGGGGAGAACGGAATTCCTGGTGTAGCGGTGAAATGCGCAGATATCAGGAGGAACACCGGTGGCGAAGGCGGTTCTCTGGGAATATCCTGACGCTGAGGAGCGAAAGTGTGGGGAGCGAACAGG	FALSE	
2	Extraction	Sphingomonadaceae	Novosphingobium	barchaimii	TACGGAGGGAGCTAGCGTTGTTCGGAATTACTGGGCGTAAAGCGCGCGTAGGCGGTTACTCAAGTCAGAGGTGAAAGCCCGGGGCTCAACCCCGGAACTGCCTTTGAAACTAGGTAACTAGAATCTTGGAGAGGTCAGTGGAATTCCGAGTGTAGAGGTGAAATTCGTAGATATTCGGAAGAACACCAGTGGCGAAGGCGACTGACTGGACAAGTATTGACGCTGAGGTGCGAAAGCGTGGGGAGCAAACAGG	TRUE	Run 2-specific contaminant
2|3	Extraction | Nested PCR	Rhodobacteraceae	Paracoccus	aestuarii/beibuensis/marinus/rhizosphaerae/zhejiangensis	TACGGAGGGGGCTAGCGTTGTTCGGAATTACTGGGCGTAAAGCGCACGTAGGCGGACTGGAAAGTTGGGGGTGAAATCCCGGGGCTCAACCTCGGAACTGCCTTCAAAACTATCAGTCTGGAGTTCGAGAGAGGTGAGTGGAATTCCGAGTGTAGAGGTGAAATTCGTAGATATTCGGAGGAACACCAGTGGCGAAGGCGGCTCACTGGCTCGATACTGACGCTGAGGTGCGAAAGCGTGGGGAGCAAACAGG	TRUE	Extraction kit 5 contaminant
1|2|3	Extraction | Nested PCR | PCR	Rhodobacteraceae	Paracoccus	aminovorans/caeni/chinensis/communis/halophilus/huijuniae	TACGGAGGGGGCTAGCGTTGTTCGGAATTACTGGGCGTAAAGCGCACGTAGGCGGACCAGAAAGTTGGGGGTGAAATCCCGGGGCTCAACCTCGGAACTGCCTTCAAAACTATTGGTCTGGAGTTCGAGAGAGGTGAGTGGAATTCCGAGTGTAGAGGTGAAATTCGTAGATATTCGGAGGAACACCAGTGGCGAAGGCGGCTCACTGGCTCGATACTGACGCTGAGGTGCGAAAGCGTGGGGAGCAAACAGG	TRUE	PCR contaminant
2|3	Extraction | Nested PCR	Rhodobacteraceae	Paracoccus	carotinifaciens/marcusii	TACGGAGGGGGCTAGCGTTGTTCGGAATTACTGGGCGTAAAGCGCACGTAGGCGGACTGGAAAGTCAGAGGTGAAATCCCAGGGCTCAACCTTGGAACTGCCTTTGAAACTATCAGTCTGGAGTTCGAGAGAGGTGAGTGGAATTCCGAGTGTAGAGGTGAAATTCGTAGATATTCGGAGGAACACCAGTGGCGAAGGCGGCTCACTGGCTCGATACTGACGCTGAGGTGCGAAAGCGTGGGGAGCAAACAGG	TRUE	Extraction kit 11 contaminant
2	Extraction | Nested PCR	Rhodobacteraceae	Paracoccus	laeviglucosivorans/yeei	TACGGAGGGGGCTAGCGTTGTTCGGAATTACTGGGCGTAAAGCGCACGTAGGCGGACCGGAAAGTCAGAGGTGAAATCCCAGGGCTCAACCTTGGAACTGCCTTTGAAACTATCGGTCTGGAGTTCGAGAGAGGTGAGTGGAATTCCGAGTGTAGAGGTGAAATTCGTAGATATTCGGAGGAACACCAGTGGCGAAGGCGGCTCACTGGCTCGATACTGACGCTGAGGTGCGAAAGCGTGGGGAGCAAACAGG	TRUE	Extraction kit 7 contaminant

Run	Detected in Control Type	Family	Genus	Species	Sequence	Removed	Removal Step/Reason
1	Extraction	Rhodobacteraceae	Paracoccus	NA	TACGGAGGGGGCTAGCGTTGTTCGGAATTACTGGGCGTAAAGCGCACGTAGGCGGACCAGAAAGTTGGGGGTGAAATCCCGGGGCTCAACCTCGGAACTGCCTTCAAAACTACTGGTCTGGAGTTCGAGAGAGGTGAGTGGAATTCCGAGTGTAGAGGTGAAATTCGTAGATATTCGGAGGAACACCAGTGGCGAAGGCGGCTCACTGGCTCGATACTGACGCTGAGGTGCGAAAGCGTGGGGAGCAAACAGG	TRUE	Run 1-specific contaminant
3	Extraction	Rhodobacteraceae	Paracoccus	NA	TACGGAGGGGGCTAGCGTTGTTCGGAATTACTGGGCGTAAAGCGCACGTAGGCGGACCAGAAAGTTGGGGGTGAAATCCCGGGGCTCAACCTCGGAACTGCCCTCAAAACTATTGGTCTGGAGTTCGAGAGAGGTGAGTGGAATTCCGAGTGTAGAGGTGAAATTCGTAGATATTCGGAGGAACACCAGTGGCGAAGGCGGCTCACTGGCTCGATACTGACGCTGAGGTGCGAAAGCGTGGGGAGCAAACAGG	TRUE	Extraction kit 11 contaminant
1|3	Extraction	Rhodobacteraceae	Paracoccus	NA	TACGGAGGGGGCTAGCGTTGTTCGGAATTACTGGGCGTAAAGCGCACGTAGGCGGACCGGAAAGTTGGGGGTGAAATCCCGGGGCTCAACCTCGGAACTGCCTTCAAAACTACTGGTCTTGAGTTCGAGAGAGGTGAGTGGAATTCCGAGTGTAGAGGTGAAATTCGTAGATATTCGGAGGAACACCAGTGGCGAAGGCGGCTCACTGGCTCGATACTGACGCTGAGGTGCGAAAGCGTGGGGAGCAAACAGG	TRUE	Extraction kit 5 contaminant
3	PCR	Sutterellaceae	Parasutterella	NA	TACGTAGGGTGCGAGCGTTAATCGGAATTACTGGGCGTAAAGGGTGTGCAGGCGGTTTTGCAAGATGGATGTGAAAGCCCCGGGCTTAACCTGGGAAAGCCATACATGACTGCAAGACTAGAGTGCGTCAGAGGGGGGTGGAATTCCAAGTGTAGCAGTGAAATGCGTAGATATTTGGAAGAACACCGATGGCGAAGGCAGCCCCCTGGGACGCAACTGACGCTCATACACGAAAGCGTGGGGAGCAAACAGG	TRUE	Run 3-specific contaminant
n/a	n/a	Hyphomicrobiaceae	Pedomicrobium 1	NA	TACGAAGGGAGCTAGCGTTGTTCGGAATCACTGGGCGTAAAGCGCACGTAGGCGGATGTGTCAGTCAGGGGTGAAATCCCGGAGCTCAACTTCGGAACTGCCCTTGATACAGCACGTCTCGAGTCCGAGAGAGGTGAGTGGAATTCCTAGTGTAGAGGTGAAATTCGTAGATATTAGGAAGAACACCAGTGGCGAAGGCGGCTCACTGGCTCGGTACTGACGCTGAGGTGCGAAAGCGTGGGGAGCAAACAGG	TRUE	Strong negative Spearman-correlation between number of reads per pooled volume and the relative abundance of the ASV
2	PCR	Comamonadaceae	Pelomonas	aquatica	TACGTAGGGTGCAAGCGTTAATCGGAATTACTGGGCGTAAAGCGTGCGCAGGCGGTTATGCAAGACAGAGGTGAAATCCCCGGGCTCAACCTGGGAACTGCCTTTGTGACTGCATGGCTAGAGTACGGTAGAGGGGGATGGAATTCCGCGTGTAGCAGTGAAATGCGTAGATATGCGGAGGAACACCGATGGCGAAGGCAATCCCCTGGACCTGTACTGACGCTCATGCACGAAAGCGTGGGGAGCAAACAGG	FALSE	
3	PCR	Bacteriovoracaceae	Peredibacter	NA	TACGAAGGGTCCAAGCGTTGTTCGGAATCATTGGGCGTAAAGCGAGCGCAGGCGGATCAGCAAGTCAGATGTGAAATCTCGAAGCTCAACTTCGAAACTGCGTCTGAAACTGCTAGTCTAGAATGTCGGAGGGGATAGGGGAATTTCACGTGTAGGGGTAAAATCCGTAGAGATGTGAAGGAACACCGGAGGCGAAGGCGCCTATCTGGACGACTATTGACGCTGAGGCTCGAAAGCGTGGGGAGCAAACAGG	TRUE	PCR contaminant
2	Extraction | Nested PCR	Intrasporangiaceae	Phycicoccus	aerophilus/bigeumensis/ginsenosidimutans	TACGTAGGGTGCGAGCGTTGTCCGGAATTATTGGGCGTAAAGAGCTTGTAGGCGGTTTGTCGCGTCTGCCGTGAAAATTCGAGGCTCAACCTCGAACTTGCGGTGGGTACGGGCAGACTAGAGTGTGGTAGGGGAGACTGGAATTCCTGGTGTAGCGGTGAAATGCGCAGATATCAGGAGGAACACCGATGGCGAAGGCAGGTCTCTGGGCCACTACTGACGCTGAGAAGCGAAAGCATGGGGAGCGAACAGG	TRUE	Run 2-specific contaminant
2	PCR	Comamonadaceae	Polaromonas	NA	TACGTAGGGTGCGAGCGTTAATCGGAATTACTGGGCGTAAAGCGTGCGCAGGCGGTTGTGCAAGACAGTTGTGAAATCCCCGGGCTCAACCTGGGAATTGCATCTGTGACTGCACGGCTAGAGTACGGTAGAGGGGGATGGAATTCCGCGTGTAGCAGTGAAATGCGTAGATATGCGGAGGAACACCGATGGCGAAGGCAATCCCCTGGACCTGTACTGACGCTCATGCACGAAAGCGTGGGGAGCAAACAGG	TRUE	Run 2-specific contaminant

Run	Detected in Control Type	Family	Genus	Species	Sequence	Removed	Removal Step/Reason
2	Extraction	Prevotellaceae	Prevotella	jejuni	TACGGAAGGTCCGGGCGTTATCCGGATTTATTGGGTTTAAAGGGAGCGTAGGCCGGAGATTAAGTGTGTTGTGAAATGTAGACGCTCAACGTCTGACTTGCAGCGCATACTGGTTTCCTTGAGTACGCACAACGTTGGCGGAATTCGTCGTGTAGCGGTGAAATGCTTAGATATGACGAAGAACTCCGATTGCGAAGGCAGCTGACGGGAGCGCAACTGACGCTTAAGCTCGAAGGTGCGGGTATCGAACAGG	FALSE	
1	Extraction | PCR	Prevotellaceae	Prevotella	oris	TACGGAAGGTCCGGGCGTTATCCGGATTTATTGGGTTTAAAGGGAGCGTAGGCCGTGGATTAAGCGTGTTGTGAAATGCAGGTGCTCAACGTCTGCACTGCAGCGCGAACTGGTCCACTTGAGTGTGCGCAACGCAGGCGGAATTCGTCGTGTAGCGGTGAAATGCTTAGATATGACGAAGAACTCCGATTGCGAAGGCAGCTTGCGGGAGCACAACTGACGCTGAAGCTCGAAAGTGCGGGTATCGAACAGG	TRUE	PCR contaminant
2	Extraction	Prevotellaceae	Prevotella	paludivivens	TACGGAAGGTCCAGGCGTTATCCGGATTTATTGGGTTTAAAGGGAGCGTAGGCCGTCTGTTAAGCGTGTTGTGAAATGTAGATGCTCAACATCTGAATTGCAGCGCGAACTGGCAGACTTGAGTGTGCGCAACGTAGGCGGAATTCGTGGTGTAGCGGTGAAATGCTTAGATATCACGAAGAACTCCAATTGCGAAGGCAGCTTACGGGAGCACAACTGACGCTGAAGCTCGAAAGTGCGGGTATCGAACAGG	TRUE	Run 2-specific contaminant
2	Extraction	Porphyromonadaceae	Proteiniphilum	NA	TACGGAGGATCCGAGCGTTATCCGGATTTATTGGGTTTAAAGGGTGCGCAGGCGGCATGTTAAGTCGGCGGTGAAATTTTGCAGCTCAACTGTAAAAGAGCCTTCGAAACTGGCAAGCTTGAGTGTGGATGAAGTAGGCGGAATTTGTGGTGTAGCGGTGAAATGCATAGATATCACAAAGAACACCGATTGCGCAGGCAGCTTACTAAACCATAACTGACGCTCATGCACGAAGGCGTGGGGATCAAACAGG	FALSE	
1	Extraction	Enterobacteriaceae	Providencia	NA	TACGGAGGGTGCAAGCGTTAATCGGAATTACTGGGCGTAAAGCGCACGCAGGCGGTTGATTAAGTTAGATGTGAAATCCCCGGGCTTAACCTGGGAATGGCATCTAAGACTGGTCAGCTAGAGTCTTGTAGAGGGGGGTAGAATTCCATGTGTAGCGGTGAAATGCGTAGAGATGTGGAGGAATACCGGTGGCGAAGGCGGCCCCCTGGACAAAGACTGACGCTCAGGTGCGAAAGCGTGGGGAGCAAACAGG	FALSE	
2	Extraction	Micrococcaceae	Pseudarthrobacter	NA	TACGTAGGGCGCAAGCGTTATCCGGAATTATTGGGCGTAAAGAGCTCGTAGGCGGTTTGTCGCGTCTGCCGTGAAAGTCCGGGGCTCAACTCCGGATCTGCGGTGGGTACGGGCAGACTAGAGTGATGTAGGGGAGACTGGAATTCCTGGTGTAGCGGTGAAATGCGCAGATATCAGGAGGAACACCGATGGCGAAGGCAGGTCTCTGGGCATTAACTGACGCTGAGGAGCGAAAGCATGGGGAGCGAACAGG	TRUE	Extraction kit 6 contaminant
1	Extraction	Bradyrhizobiaceae	Pseudolabrys	NA	TACGAAGGGGGCTAGCGTTGCTCGGAATTACTGGGCGTAAAGCGCACGTAGGCGGCTTTCTAAGTCGGGGGTGAAATCCTGGAGCTCAACTCCAGAACTGCCTTCGATACTGGAAAGCTCGAGTCCGGGAGAGGTGAGTGGAACTGCGAGTGTAGAGGTGAAATTCGTAGATATTCGCAAGAACACCAGTGGCGAAGGCGGCTCACTGGCCCGGTACTGACGCTGAGGTGCGAAAGCGTGGGGAGCAAACAGG	TRUE	Extraction kit 2 contaminant
3	Extraction	Pseudomonadaceae	Pseudomonas	brenneri/gessardii/proteolytica/veronii	TACAGAGGGTGCAAGCGTTAATCGGAATTACTGGGCGTAAAGCGCGCGTAGGTGGTTAGTTAAGTTGGATGTGAAATCCCCGGGCTCAACCTGGGAACTGCATTCAAAACTGACTGACTAGAGTATGGTAGAGGGTGGTGGAATTTCCTGTGTAGCGGTGAAATGCGTAGATATAGGAAGGAACACCAGTGGCGAAGGCGACCACCTGGACTGATACTGACACTGAGGTGCGAAAGCGTGGGGAGCAAACAGG	TRUE	Extraction kit 9 contaminant
1	Extraction	Pseudomonadaceae	Pseudomonas	NA	TACGAAGGGTGCAAGCGTTAATCGGAATTACTGGGCGTAAAGCGCGCGTAGGTGGTTCGTTAAGTTGGATGTGAAAGCCCCGGGCTCAACCTGGGAACTGCATCCAAAACTGGCGAGCTAGAGTACGGTAGAGGGTGGTGGAATTTCCTGTGTAGCGGTGAAATGCGTAGATATAGGAAGGAACACCAGTGGCGAAGGCGACCACCTGGACTGATACTGACACTGAGGTGCGAAAGCGTGGGGAGCAAACAGG	FALSE	

Run	Detected in Control Type	Family	Genus	Species	Sequence	Removed	Removal Step/Reason
3	Extraction	Pseudomonadaceae	Pseudomonas	oryzihabitans/psychrotolerans	TACGAAGGGTGCAAGCGTTAATCGGAATTACTGGGCGTAAAGCGCGCGTAGGTGGCTTGATAAGTTGGATGTGAAATCCCCGGGCTCAACCTGGGAACTGCATCCAAAACTGTCTGGCTAGAGTGCGGTAGAGGGTAGTGGAATTTCCAGTGTAGCGGTGAAATGCGTAGATATTGGAAGGAACACCAGTGGCGAAGGCGACTACCTGGACTGACACTGACACTGAGGTGCGAAAGCGTGGGGAGCAAACAGG	FALSE	
n/a	n/a	Pseudomonadaceae	Pseudomonas 5	antarctica/costantinii/extremorientalis/fluorescens/lurida/meridiana/poae/simiae/trivialis	TACAGAGGGTGCAAGCGTTAATCGGAATTACTGGGCGTAAAGCGCGCGTAGGTGGTTTGTTAAGTTGGATGTGAAATCCCCGGGCTCAACCTGGGAACTGCATTCAAAACTGACTGACTAGAGTATGGTAGAGGGTGGTGGAATTTCCTGTGTAGCGGTGAAATGCGTAGATATAGGAAGGAACACCAGTGGCGAAGGCGACCACCTGGACTAATACTGACACTGAGGTGCGAAAGCGTGGGGAGCAAACAGG	TRUE	Strong negative Spearman-correlation between number of reads per pooled volume and the relative abundance of the ASV
3	Extraction	Ralstonia_f	Ralstonia	insidiosa/syzygii	TACGTAGGGTCCAAGCGTTAATCGGAATTACTGGGCGTAAAGCGTGCGCAGGCGGTTGTGCAAGACCGATGTGAAATCCCCGGGCTTAACCTGGGAATTGCATTGGTGACTGCACGGCTAGAGTGTGTCAGAGGGGGGTAGAATTCCACGTGTAGCAGTGAAATGCGTAGAGATGTGGAGGAATACCGATGGCGAAGGCAGCCCCCTGGGATAACACTGACGCTCATGCACGAAAGCGTGGGGAGCAAACAGG	TRUE	Run 3-specific contaminant
1	PCR	Ralstonia_f	Ralstonia	pickettii	TACGTAGGGTCCAAGCGTTAATCGGAATTACTGGGCGTAAAGCGTGCGCAGGCGGTTGTGCAAGACCGATGTGAAATCCCCGAGCTTAACTTGGGAATTGCATTGGTGACTGCACGGCTAGAGTGTGTCAGAGGGGGGTAGAATTCCACGTGTAGCAGTGAAATGCGTAGAGATGTGGAGGAATACCGATGGCGAAGGCAGCCCCCTGGGATAACACTGACGCTCATGCACGAAAGCGTGGGGAGCAAACAGG	TRUE	PCR contaminant
1	Extraction	Alishewanella_f	Rheinheimera	tangshanensis	TACAGAGGGTGCAAGCGTTAATCGGAATTACTGGGCGTAAAGCGCACGTAGGCGGTTTTTTAAGTCAGATGTGAAAGCCCCGGGCTCAACCTGGGAATTGCATTTGAAACTGGGGAACTAGAGTGTGTGAGAGGGGGGTAGAATTCCAAGTGTAGCGGTGAAATGCGTAGAGATTTGGAGGAATACCAGTGGCGAAGGCGGCCCCCTGGCACAACACTGACGCTCAGGTGCGAAAGCGTGGGGAGCAAACAGG	TRUE	Run 1-specific contaminant
2	PCR	Comamonadaceae	Rhizobacter	bergeniae/dauci/gummiphilus	TACGTAGGGTGCAAGCGTTAATCGGAATTACTGGGCGTAAAGCGTGCGCAGGCGGCTATGCAAGACAGATGTGAAATCCCCGGGCTCAACCTGGGAACTGCATTTGTGACTGCATGGCTAGAGTACGGTAGAGGGGGATGGAATTCCGCGTGTAGCAGTGAAATGCGTAGATATGCGGAGGAACACCGATGGCGAAGGCAATCCCCTGGACCTGTACTGACGCTCATGCACGAAAGCGTGGGGAGCAAACAGG	TRUE	PCR contaminant
1	Extraction	Rhizobiaceae	Rhizobium	NA	TACGAAGGGGGCTAGCGTTGTTCGGAATTACTGGGCGTAAAGCGCACGTAGGCGGATATTTAAGTCAGGGGTGAAATCCCAGAGCTCAACTCTGGAACTGCCTTTGATACTGGGTATCTTGAGTATGGAAGAGGTAAGTGGAATTCCGAGTGTAGAGGTGAAATTCGTAGATATTCGGAGGAACACCAGTGGCGAAGGCGGCTTACTGGTCCATTACTGACGCTGAGGTGCGAAAGCGTGGGGAGCAAACAGG	FALSE	
2	PCR	Rhizobiaceae	Rhizobium	NA	TACGAAGGGGGCTAGCGTTGTTCGGAATTACTGGGCGTAAAGCGCACGTAGGCGGATATTTAAGTCAGGGGTGAAATCCCGCAGCTCAACTGCGGAACTGCCTTTGATACTGGGTATCTTGAGTATGGAAGAGGTAAGTGGAATTCCGAGTGTAGAGGTGAAATTCGTAGATATTCGGAGGAACACCAGTGGCGAAGGCGGCTTACTGGTCCATTACTGACGCTGAGGTGCGAAAGCGTGGGGAGCAAACAGG	FALSE	
1|2	Extraction | Nested PCR	Micrococcaceae	Rothia	dentocariosa	TACGTAGGGCGCGAGCGTTGTCCGGAATTATTGGGCGTAAAGAGCTTGTAGGCGGTTGGTCGCGTCTGCTGTGAAAGGCTGGGGCTTAACCCTGGTTTTGCAGTGGGTACGGGCTAACTAGAGTGCAGTAGGGGAGACTGGAATTCCTGGTGTAGCGGTGGAATGCGCAGATATCAGGAGGAACACCGATGGCGAAGGCAGGTCTCTGGGCTGTAACTGACGCTGAGAAGCGAAAGCATGGGGAGCGAACAGG	FALSE	

Run	Detected in Control Type	Family	Genus	Species	Sequence	Removed	Removal Step/Reason
2	Extraction	Micrococcaceae	Rothia	NA	TACGTAGGGCGCGAGCGTTGTCCGGAATTATTGGGCGTAAAGAGCTTGTAGGCGGTTTGTCGCGTCTGCTGTGAAAGGCCGGAGCTTAACTCCGTGTATTGCAGTGGGTACGGGCAGACTAGAGTGCAGTAGGGGAGACTGGAATTCCTGGTGTAGCGGTGGAATGCGCAGATATCAGGAGGAACACCGATGGCGAAGGCAGGTCTCTGGGCTGTAACTGACGCTGAGAAGCGAAAGCATGGGGAGCGAACAGG	FALSE	
2	Extraction	Ruminococcaceae	Saccharofermentans	NA	TACGTAGGTGGCAAGCGTTATCCGGATTTACTGGGTGTAAAGGGCGTGTAGGCGGTTCTGCAAGTCAGATGTGAAATTCCCGGGCTTAACCCGGGCGCTGCATCTGAAACTGCAGAACTTGAGTACTGGAGAGGATAGTGGAATTCCTAGTGTAGCGGTAAAATGCGTAGATATTAGGAGGAACACCAGTGGCGAAGGCGGCTATCTGGACAGTAACTGACGCTGAGGCGCGAAAGCGTGGGGAGCAAACAGG	TRUE	Extraction kit 7 contaminant
1	Extraction	Sphingomonadaceae	Sphingomonas	flavus	TACGGAGGGGGCTAGCGTTGTTCGGAATTACTGGGCGTAAAGCGCGCGTAGGCGGCTTTGTAAGTTAGAGGTGAAAGCCCGGAGCTCAACTCCGGAATTGCCTTTAAGACTGCATCGCTAGAATCATGGAGAGGTGAGTGGAATTCCGAGTGTAGAGGTGAAATTCGTAGATATTCGGAAGAACACCAGTGGCGAAGGCGACTCACTGGACATGTATTGACGCTGAGGTGCGAAAGCGTGGGGAGCAAACAGG	TRUE	Extraction kit 2 contaminant
1	PCR	Sphingomonadaceae	Sphingomonas	NA	TACGGAGGGGGCTAGCGTTGTTCGGAATTACTGGGCGTAAAGCGCACGTAGGCGGCTTTGTAAGTTAGAGGTGAAAGCCCGGAGCTCAACTCCGGAACTGCCTTTAAGACTGCATCGCTCGAATCATGGAGAGGTCAGTGGAATTCCGAGTGTAGAGGTGAAATTCGTAGATATTCGGAAGAACACCAGTGGCGAAGGCGACTGACTGGACATGTATTGACGCTGAGGTGCGAAAGCGTGGGGAGCAAACAGG	TRUE	PCR contaminant
2	PCR	Sphingomonadaceae	Sphingomonas	NA	TACGGAGGGGGCTAGCGTTGTTCGGAATTACTGGGCGTAAAGCGCACGTAGGCGGCTTTGTAAGTTAGAGGTGAAAGCCCGGGGCTCAACTCCGGAATTGCCTTTAAGACTGCATCGCTAGAATTGTGGAGAGGTAAGTGGAATTCCGAGTGTAGAGGTGAAATTCGTAGATATTCGGAAGAACACCAGTGGCGAAGGCGACTTACTGGACACATATTGACGCTGAGGTGCGAAAGCGTGGGGAGCAAACAGG	TRUE	Run 2-specific contaminant
2	Extraction	Sphingomonadaceae	Sphingomonas	soli	TACGGAGGGAGCTAGCGTTATTCGGAATTACTGGGCGTAAAGCGTACGTAGGCGGCTTTGCAAGTTAGAGGTGAAAGCCTGGAGCTCAACTCCAGAACTGCCTTTAAAACTGCATCGCTTGAATCCAGGAGAGGTGAGTGGAATTCCGAGTGTAGAGGTGAAATTCGTAGATATTCGGAAGAACACCAGTGGCGAAGGCGGCTCACTGGACTGGTATTGACGCTGAGGTACGAAAGCGTGGGGAGCAAACAGG	TRUE	Run 2-specific contaminant
3	PCR	Sphingomonadaceae	Sphingopyxis	alaskensis/bauzanensis/chilensis/fribergensis/panaciterrae	TACGGAGGGAGCTAGCGTTGTTCGGAATTACTGGGCGTAAAGCGCGCGTAGGCGGTTTTTTAAGTCAGAGGTGAAAGCCCAGTGCTCAACACTGGAACTGCCTTTGAAACTGGAAAACTTGAATCTTGGAGAGGTCAGTGGAATTCCGAGTGTAGAGGTGAAATTCGTAGATATTCGGAAGAACACCAGTGGCGAAGGCGACTGACTGGACAAGTATTGACGCTGAGGTGCGAAAGCGTGGGGAGCAAACAGG	TRUE	PCR contaminant
1|2|3	Extraction | PCR	Staphylococcaceae	Staphylococcus	argenteus/aureus/capitis/caprae/epidermidis/haemolyticus/hominis/lugdunensis/pasteuri/petrasii/saccharolyticus/schweitzeri/simiae/warneri	TACGTAGGTGGCAAGCGTTATCCGGAATTATTGGGCGTAAAGCGCGCGTAGGCGGTTTTTTAAGTCTGATGTGAAAGCCCACGGCTCAACCGTGGAGGGTCATTGGAAACTGGAAAACTTGAGTGCAGAAGAGGAAAGTGGAATTCCATGTGTAGCGGTGAAATGCGCAGAGATATGGAGGAACACCAGTGGCGAAGGCGACTTTCTGGTCTGTAACTGACGCTGATGTGCGAAAGCGTGGGGATCAAACAGG	FALSE	
1	PCR	Staphylococcaceae	Staphylococcus	carnosus/condimenti/piscifermentans/simulans	TACGTAGGTGGCAAGCGTTATCCGGAATTATTGGGCGTAAAGCGCGCGTAGGCGGTTTTTTAAGTCTGATGTGAAAGCCCACGGCTCAACCGTGGAGGGTCATTGGAAACTGGAAAACTTGAGTGCAGAAGAGGAAAGTGGAATTCCATGTGTAGCGGTGAAATGCGCAGAGATATGGAGGAACACCAGTGGCGAAGGCGACTTTCTGGTCTGCAACTGACGCTGATGTGCGAAAGCGTGGGGATCAAACAGG	FALSE	

Run	Detected in Control Type	Family	Genus	Species	Sequence	Removed	Removal Step/Reason
2|3	Extraction | Nested PCR	Staphylococcaceae	Staphylococcus	cohnii/gallinarum/saprophyticus/succinus/xylosus	TACGTAGGTGGCAAGCGTTATCCGGAATTATTGGGCGTAAAG<br>CGCGCGTAGGCGGTTTCTTAAGTCTGATGTGAAAGCCCACGGC<br>TCAACCGTGGAGGGTCATTGGAAACTGGGAAACTTGAGTGCA<br>GAAGAGGAAAGTGGAATTCCATGTGTAGCGGTGAAATGCGCA<br>GAGATATGGAGGAACACCAGTGGCGAAGGCGACTTTCTGGTC<br>TGTAACTGACGCTGATGTGCGAAAGCGTGGGGATCAAACAGG	TRUE	Extraction kit 10 contaminant
2	Extraction	Staphylococcaceae	Staphylococcus	NA	TACGTAGGTGGCAAGCGTTATCCGGAATTATTGGGCGTAAAG<br>CGCGCGTAGGCGGTTTTTTAAGTCTGATGTGAAAGCCCACGGC<br>TCAACCGTGGAGGGTCATTGGAAACTGTAAAACTTGAGTGCA<br>GAAGAGGAAAGTGGAATTCCATGTGTAGCGGTGAAATGCGCA<br>GAGATATGGAGGAACACCAGTGGCGAAGGCGACTTTCTGGTC<br>TGTAACTGACGCTGATGTGCGAAAGCGTGGGGATCAAACAGG	FALSE	
2	Extraction	Staphylococcaceae	Staphylococcus	pettenkoferi	TACGTAGGTGGCAAGCGTTATCCGGAATTATTGGGCGTAAAG<br>CGCGCGTAGGCGGTTTCTTAAGTCTGATGTGAAAGCCCACGGC<br>TCAACCGTGGAGGGTCATTGGAAACTGGGAAACTTGAGTGCA<br>GGAGAGGAAAGTGGAATTCCATGTGTAGCGGTGAAATGCGCA<br>GAGATATGGAGGAACACCAGTGGCGAAGGCGACTTTCTGGTC<br>TGCAACTGACGCTGATGTGCGAAAGCGTGGGGATCAAACAGG	FALSE	
2	Extraction	Xanthomonadaceae	Stenotrophomonas	NA	TACGAAGGGTGCAAGCGTTACTCGGAATTACTGGGCGTAAAG<br>CGTGCGTAGGTGGTTGTTTAAGTCTGTTGTGAAAGCCCTGGGC<br>TCAACCTGGGAATTGCAGTGGAAACTGGACGACTAGAGTGTG<br>GTAGAGGGTAGTGGAATTCCCGGTGTAGCAGTGAAATGCGTA<br>GAGATCGGGAGGAACATCCATGGCGAAGGCAGCTACCTGGAC<br>CAACACTGACACTGAGGCACGAAAGCGTGGGGAGCAAACAG<br>G	TRUE	Run 2-specific contaminant
1	Extraction	Streptococcaceae	Streptococcus	constellatus	TACGTAGGTCCCGAGCGTTGTCCGGATTTATTGGGCGTAAAGC<br>GAGCGCAGGCGGTTAGATAAGTCTGAAGTGAAAGGCAGTGGC<br>TCAACCATTGTAGGCTTTGGAAACTGTTTAACTTGAGTGCAGA<br>AGGGGAGAGTGGAATTCCATGTGTAGCGGTGAAATGCGTAGA<br>TATATGGAGGAACACCGGTGGCGAAAGCGGCTCTCTGGTCTG<br>TAACTGACGCTGAGGCTCGAAAGCGTGGGGAGCGAACAGG	TRUE	Extraction kit 4 contaminant
3	Extraction	Streptococcaceae	Streptococcus	equinus/infantarius/lutetiensis	TACGTAGGTCCCGAGCGTTGTCCGGATTTATTGGGCGTAAAGC<br>GAGCGCAGGCGGTTTAATAAGTCTGAAGTTAAAGGCAGTGGC<br>TTAACCATTGTTCGCTTTGGAAACTGTTAGACTTGAGTGCAGA<br>AGGGGAGAGTGGAATTCCATGTGTAGCGGTGAAATGCGTAGA<br>TATATGGAGGAACACCGGTGGCGAAAGCGGCTCTCTGGTCTG<br>TAACTGACGCTGAGGCTCGAAAGCGTGGGGAGCAAACAGG	TRUE	Extraction kit 10 contaminant
1	Extraction	Streptococcaceae	Streptococcus	NA	TACGTAGGTCCCGAGCGTTGTCCGGATTTATTGGGCGTAAAGC<br>GAGCGCAGGCGGTTAGATAAGTCTGAAGTTAAAGGCTGTGGC<br>TTAACCATAGTACGCTTTGGAAACTGTTTAACTTGAGTGCAAG<br>AGGGGAGAGTGGAATTCCATGTGTAGCGGTGAAATGCGTAGA<br>TATATGGAGGAACACCAGTGGCGAAAGCGGCTCTCTGGCTTG<br>TAACTGACGCTGAGGCTCGAAAGCGTGGGGAGCAAACAGG	TRUE	Run 1-specific contaminant
1	Extraction	Streptococcaceae	Streptococcus	NA	TACGTAGGTCCCGAGCGTTGTCCGGATTTATTGGGCGTAAAGC<br>GAGCGCAGGCGGTTTGATAAGTCTGAAGTTAAAGGCTGTGGC<br>TCAACCATAGTTCGCTTTGGAAACTGTCAAACTTGAGTGCAGA<br>AGGGGAGAGTGGAATTCCATGTGTAGCGGTGAAATGCGTAGA<br>TATATGGAGGAACACCGGTGGCGAAAGCGGCTCTCTGGTCTG<br>TAACTGACGCTGAGGCTCGAAAGCGTGGGGAGCGAACAGG	FALSE	
1	Extraction	Streptococcaceae	Streptococcus	NA	TACGTAGGTCCCGAGCGTTATCCGGATTTATTGGGCGTAAAGC<br>GAGCGCAGGCGGTTAGATAAGTCTGAAGTTAAAGGCTGTGGC<br>TTAACCATAGTACGCTTTGGAAACTGTTTAACTTGAGTGCAGA<br>AGGGGAGAGTGGAATTCCATGTGTAGCGGTGAAATGCGTAGA<br>TATATGGAGGAACACCGGTGGCGAAAGCGGCTCTCTGGTCTG<br>TAACTGACGCTGAGGCTCGAAAGCGTGGGGAGCAAACAGG	FALSE	

Run	Detected in Control Type	Family	Genus	Species	Sequence	Removed	Removal Step/Reason
1|2	Extraction	Streptococcaceae	Streptococcus	NA	TACGTAGGTCCCGAGCGTTGTCCGGATTTATTGGGCGTAAAGCGAGCGCAGGCGGTTAGATAAGTCTGAAGTTAAAGGCTGTGGCTTAACCATAGTACGCTTTGGAAACTGTTTAACTTGAGTGCAAGAGGGGAGAGTGGAATTCCATGTGTAGCGGTGAAATGCGTAGATATATGGAGGAACACCGGTGGCGAAAGCGGCTCTCTGGCTTGTAACTGACGCTGAGGCTCGAAAGCGTGGGGAGCAAACAGG	FALSE	
1	Extraction	Comamonadaceae	Tepidimonas	NA	TACGTAGGGTGCGAGCGTTAATCGGAATTACTGGGCGTAAAGCGTGCGCAGGCGGTTGTGTAAGACAGGCGTGAAATCCCCGGGCTCAACCTGGGAATGGCGCTTGTGACTGCACGGCTGGAGTGCGGCAGAGGGGGATGGAATTCCGCGTGTAGCAGTGAAATGCGTAGATATGCGGAGGAACACCGATGGCGAAGGCAATCCCCTGGGCCTGCACTGACGCTCATGCACGAAAGCGTGGGGAGCAAACAGG	TRUE	Extraction kit 3 contaminant
2	Extraction	Comamonadaceae	Tepidimonas	NA	TACGTAGGGTGCGAGCGTTAATCGGAATTACTGGGCGTAAAGCGTGCGCAGGCGGTCTTGTAAGACAGAGGTGAAATCCCTGGGCTCAACCTAGGAATGGCCTTTGTGACTGCAAGGCTGGAGTGCGGCAGAGGGGGATGGAATTCCGCGTGTAGCAGTGAAATGCGTAGATATGCGGAGGAACACCGATGGCGAAGGCAGTCCCCTGGGCCTGCACTGACGCTCATGCACGAAAGCGTGGGGAGCAAACAGG	FALSE	
2	Extraction	Hydrogenophilaceae	Tepidiphilus	succinatimandens	TACGTAGGGTGCGAGCGTTAATCGGAATTACTGGGCGTAAAGGGTGCGCAGGCGGCCCTGTAAGACCGGTGTGAAATCCCCGGGCTCAACCTGGGAATTGCGCTGGTGACTGCAGGGCTAGAGTACGGTAGAGGGGGGTGGAATTCCTGGTGTAGCAGTGAAATGCGTAGAGATCAGGAGGAACACCGATGGCGAAGGCAGCCCCCTGGGCCTGTACTGACGCTCATGCACGAAAGCGTGGGGAGCAAACAGG	FALSE	
1|2	Extraction	Thermoactinomycetaceae	Thermicanus	NA	TACGTAGGGGGCGAGCGTTGTCCGGAATGATTGGGCGTAAAGCGCGCGCAGGCGGTCCTTTAAGTCTGATGTAAAAGCCCGCGGCTTAACCGCGGAAGGTCATTGGAAACTGGGGGACTTGAGGCTAGGAGAGGGAAGTGGAATTCCTGGTGTAGCGGTGAAATGCGTAGAGATCAGGAGGAATACCGATGGCGAAAGCAACTTCCTGGCTTAGAACTGACGCTGAGGCGCGAAAGCGTGGGGAGCAAACAGG	TRUE	Extraction kit 3 contaminant
1	PCR	Trueperaceae	Truepera	NA	TACGGAGGGTGCAAGCGTTATCCGGAATCACTGGGCGTAAAGGGCGCGTAGGCGGTTTGTTAAGTCCGATGTTAAAGACCGAGGCTCAACCTCGACACGGCGTTGGATACTGGCAAGCTGGACGGTTGGAGAGGAAGGTAGAATTACCAGTGTAGCGGTGGAATGCGTAGATACTGGTAGGAATACCCATTGCGAAGGCAGCCTTCTGGACAACACGTGACGCTGAGGCGCGAAAGTGTGGGGAGCAAACCGG	TRUE	PCR contaminant
1|2	Extraction | PCR	Corynebacteriaceae	Turicella	otitidis	TACGTAGGGTGCGAGCGTTGTCCGGATTTACTGGGCGTAAAGAGCTCGTAGGTGGCTTGTCGCGTCGTCTGTGAAAGTCTGGGGCTTAACTCCGGGTGTGCAGGCGATACGGGCTGGCTTGAGTGCTGTAGGGGAGACTGGAATTCCTGGTGTAGCGGTGGAATGCGCAGATATCAGGAGGAACACCGATGGCGAAGGCAGGTCTCTGGGCAGTCACTGACGCTGAGGAGCGAGAGCATGGGTAGCGAACAGG	FALSE	
1|2	Extraction | PCR | Nested PCR	Oxalobacteraceae	Undibacterium	oligocarboniphilum	TACGTAGGGTGCAAGCGTTAATCGGAATTACTGGGCGTAAAGCGTGCGCAGGCGGTTTTGTAAGTCTGATGTGAAATCCCCGGGCTCAACCTGGGAACTGCATTGGAGACTGCAAGGCTAGAGTGTGTCAGAGGGGGGTAGAATTCCACGTGTAGCAGTGAAATGCGTAGAGATGTGGAGGAATACCGATGGCGAAGGCAGCCCCCTGGGATAACACTGACGCTCATGCACGAAAGCGTGGGGAGCAAACAGG	TRUE	PCR contaminant
2	PCR	Comamonadaceae	Variovorax	boronicumulans/ginsengisoli	TACGTAGGGTGCAAGCGTTAATCGGAATTACTGGGCGTAAAGCGTGCGCAGGCGGTTATGTAAGACAGTTGTGAAATCCCCGGGCTCAACCTGGGAACTGCATCTGTGACTGCATAGCTAGAGTACGGTAGAGGGGGATGGAATTCCGCGTGTAGCAGTGAAATGCGTAGATATGCGGAGGAACACCGATGGCGAAGGCAATCCCCTGGACCTGTACTGACGCTCATGCACGAAAGCGTGGGGAGCAAACAGG	TRUE	PCR contaminant

Run	Detected in Control Type	Family	Genus	Species	Sequence	Removed	Removal Step/Reason
3	Extraction	Veillonellaceae	Veillonella	parvula/tobetsuensis	TACGTAGGTGGCAAGCGTTGTCCGGAATTATTGGGCGTAAAGCGCGCGCAGGCGGATCAGTCAGTCTGTCTTAAAAGTTCGGGGCTTAACCCCGTGATGGGATGGAAACTGCTGATCTAGAGTATCGGAGAGGAAAGTGGAATTCCTAGTGTAGCGGTGAAATGCGTAGATATTAGGAAGAACACCAGTGGCGAAGGCGACTTTCTGGACGAAAACTGACGCTGAGGCGCGAAAGCCAGGGGAGCGAACGGG	FALSE	
