# Supplementary material for: Case-Control Microbiome Study of Chronic Otitis Media with Effusion in Children Points at Streptococcus salivarius as a Pathobiont-Inhibiting Species
Source: mSystems. 2021 Apr 20;6(2):e00056-21. doi: 10.1128/mSystems.00056-21 (PMC8546964; doi:10.1128/mSystems.00056-21)
Supplement: TABLE S3 [file msystems.00056-21-st003.pdf]

Table S3: Prevalence and mean relative abundance of nasopharynx ASVs shared between OME patients and healthy controls. The healthy controls were considered cumulatively (HNPAll) and individually (HNPCI = Cochlear implant recipients, HNPDC = day-care children). To calculate the mean relative abundance, only samples in which the taxon was present was considered.

		Prevalence				Mean Relative Abundance				
Taxon Name	Species	DNP (n=67)	HNP_{All} (n=51)	HNP_{CI} n=10	HNP_{DC} (n=41)	DNP (n=67)	HNP_{All} (n=51)	HNP_{CI} (n=10)	HNP_{DC} (n=41)	Sequence
Abiotrophia 1	defectiva	24%	25%	30%	24%	0,1%	0,1%	0,0%	0,1%	TACGTAGGTGGCGAGCGTTGTCCGGATTTATTGGGCGTAAAGGGAGTGTAGGCGGTCTTTTAAGTCTGATGTGAAAGCCCACGGCTCAACCGTGGAGGGTCATTGGAAACTGGGAGACTTGAGTGCAGAAGAGGAGAGCGGAATTCCATGTGTAGCGGTGAAATGCGTAGATATATGGAGGAACACCAGTGGCGAAGGCGGCTCTCTGGTCTGTAACTGACGCTGAGGCTCGAAAGCGTGGGGAGCAAACAGG
Acinetobacter 1	lwoffii/pseudolwoffii	3%	33%	20%	37%	0,3%	0,1%	0,1%	0,1%	TACAGAGGGTGCAAGCGTTAATCGGATTTACTGGGCGTAAAGCGCGCGTAGGTGGCCAATTAAGTCAAATGTGAAATCCCCGAGCTTAACTTGGGAATTGCATTCGATACTGGTTGGCTAGAGTATGGGAGAGGATGGTAGAATTCCAGGTGTAGCGGTGAAATGCGTAGAGATCTGGAGGAATACCGATGGCGAAGGCAGCCATCTGGCCTAATACTGACACTGAGGTGCGAAAGCATGGGGAGCAAACAGG
Acinetobacter 3	johnsonii/oryzae	6%	12%	20%	10%	0,0%	0,1%	0,0%	0,1%	TACAGAGGGTGCGAGCGTTAATCGGATTTACTGGGCGTAAAGCGTGCGTAGGCGGCTTTTTAAGTCGGATGTGAAATCCCTGAGCTTAACTTAGGAATTGCATTCGATACTGGGAAGCTAGAGTATGGGAGAGGATGGTAGAATTCCAGGTGTAGCGGTGAAATGCGTAGAGATCTGGAGGAATACCGATGGCGAAGGCAGCCATCTGGCCTAATACTGACGCTGAGGTACGAAAGCATGGGGAGCAAACAGG
Acinetobacter 4	baumannii	1%	10%	10%	10%	0,0%	0,1%	0,2%	0,1%	TACAGAGGGTGCGAGCGTTAATCGGATTTACTGGGCGTAAAGCGTGCGTAGGCGGCTTATTAAGTCGGATGTGAAATCCCCGAGCTTAACTTGGGAATTGCATTCGATACTGGTGAGCTAGAGTATGGGAGAGGATGGTAGAATTCCAGGTGTAGCGGTGAAATGCGTAGAGATCTGGAGGAATACCGATGGCGAAGGCAGCCATCTGGCCTAATACTGACGCTGAGGTACGAAAGCATGGGGAGCAAACAGG
Actinobacillus 1 (Haemophilus or Conservatibacter)	H. parainfluenzae or C. flavescens	60%	57%	70%	54%	0,8%	0,7%	1,1%	0,5%	TACGGAGGGTGCGAGCGTTAATCGGAATAACTGGGCGTAAAGGGCACGCAGGCGGTGACTTAAGTGAGGTGTGAAAGCCCCGGGCTTAACCTGGGAATTGCATTTCATACTGGGTCGCTAGAGTACTTTAGGGAGGGGTAGAATTCCACGTGTAGCGGTGAAATGCGTAGAGATGTGGAGGAATACCGAAGGCGAAGGCAGCCCCTTGGGAATGTACTGACGCTCATGTGCGAAAGCGTGGGGAGCAAACAGG
Actinobacillus 2 (Haemophilus)	parahaemolyticus/paraphrohaemolyticus/sputorum	27%	10%	20%	7%	1,3%	0,3%	0,3%	0,2%	TACGGGGGGTGCGAGCGTTAATCGGAATAACTGGGCGTAAAGGGCACGCAGGCGGTGACTTAAGTGAGATGTGAAAGCCCCGGGCTTAACCTGGGAATTGCATTTCATACTGGGTCGCTAGAGTACTTTAGGGAGGGGTAGAATTCCACGTGTAGCGGTGAAATGCGTAGAGATGTGGAGGAATACCGAAGGCGAAGGCAGCCCCTTGGGAATGTACTGACGCTCATGTGCGAAAGCGTGGGGAGCAAACAGG
Actinobacillus 3 (Haemophilus)	NA	34%	4%	10%	2%	0,9%	0,6%	0,9%	0,3%	TACGGGGGGTGCGAGCGTTAATCGGAATAACTGGGCGTAAAGGGCACGCAGGCGGTGACTTAAGTGAGATGTGAAAGCCCCGAGCTTAACTTGGGAATTGCATTTCATACTGGGTCGCTAGAGTACTTTAGGGAGGGGTAGAATTCCACGTGTAGCGGTGAAATGCGTAGAGATGTGGAGGAATACCGAAGGCGAAGGCAGCCCCTTGGGAATGTACTGACGCTCATGTGCGAAAGCGTGGGGAGCAAACAGG
Actinomyces 1 (Schaalia)	odontolytica	25%	24%	30%	22%	0,1%	0,4%	0,2%	0,5%	TACGTAGGGCGCGAGCGTTGTCCGGAATTATTGGGCGTAAAGGGCTTGTAGGCGGTTGGTCGCGTCTGCCGTGAAATCCTCTGGCTTAACTGGGGGCGTGCGGTGGGTACGGGCTGACTTGAGTGCGGTAGGGGAGACTGGAACTCCTGGTGTAGCGGTGGAATGCGCAGATATCAGGAAGAACACCGGTGGCGAAGGCGGGTCTCTGGGCCGTTACTGACGCTGAGGAGCGAAAGCGTGGGGAGCGAACAGG
Actinomyces 2	naeslundii/oris	21%	25%	10%	29%	0,2%	0,2%	0,1%	0,2%	TACGTAGGGCGCGAGCGTTGTCCGGAATTATTGGGCGTAAAGAGCTCGTAGGCGGCTGGTCGCGTCTGTCGTGAAATCCTCTGGCTTAACTGGGGGCTTGCGGTGGGTACGGGCCGGCTTGAGTGCGGTAGGGGAGACTGGAACTCCTGGTGTAGCGGTGGAATGCGCAGATATCAGGAAGAACACCGGTGGCGAAGGCGGGTCTCTGGGCCGTTACTGACGCTGAGGAGCGAAAGCGTGGGGAGCGAACAGG
Actinomyces 5	NA	9%	4%	10%	2%	0,1%	0,1%	0,0%	0,2%	TACGTAGGGTGCGAGCGTTGTCCGGAATTATTGGGCGTAAAGGGCTTGTAGGCGGTTTGTCGCGTCTGCCGTGAAATCCTCTGGCTTAACTGGGGGCGTGCGGTGGGTACGGGCAGGCTTGAGTGCGGTAGGGGAGACTGGAACTCCTGGTGTAGCGGTGGAATGCGCAGATATCAGGAAGAACACCGGTGGCGAAGGCGGGTCTCTGGGCCGTTACTGACGCTGAGGAGCGAAAGCGTGGGGAGCGAACAGG
Aggregatibacter 2	aphrophilus/kilianii	4%	4%	10%	2%	0,1%	0,4%	0,8%	0,0%	TACGGAGGGTGCGAGCGTTAATCGGAATAACTGGGCGTAAAGGGCACGCAGGCGGACTTTTAAGTGAGGTGTGAAATCCCCGGGCTTAACCTGGGAATTGCATTTCAGACTGGGGGTCTAGAGTACTTTAGGGAGGGGTAGAATTCCACGTGTAGCGGTGAAATGCGTAGAGATGTGGAGGAATACCGAAGGCGAAGGCAGCCCCTTGGGAATGTACTGACGCTCATGTGCGAAAGCGTGGGGAGCAAACAGG

		Prevalence				Mean Relative Abundance				
		DNP	HNP_{All}	HNP_{CI}	HNP_{DC}	DNP	HNP_{All}	HNP_{CI}	HNP_{DC}	
**Taxon Name**	**Species**	**(n=67)**	**(n=51)**	**n=10**	**(n=41)**	**(n=67)**	**(n=51)**	**(n=10)**	**(n=41)**	**Sequence**
Aggregatibacter 4	segnis	3%	4%	10%	2%	0,1%	0,1%	0,1%	0,2%	TACGGAGGGTGCGAGCGTTAATCGGAATAACTGGGCGTAAAGGGCACGCAGGCGGACTTTTAAGTGAGGTGTGAAATCCCCGGGCTTAACCTGGGAATTGCATTTCAGACTGGGAGTCTAGAGTACTTTAGGGAGGGGTAGAATTCCACGTGTAGCGGTGAAATGCGTAGAGATGTGGAGGAATACCGAAGGCGAAGGCAGCCCCTTGGGAATGTACTGACGCTCATGTGCGAAAGCGTGGGGAGCAAACAGG
Alloiococcus 1	otitis	27%	14%	30%	10%	0,1%	0,0%	0,0%	0,0%	TACGTAGGTGACAAGCGTTGTCCGGATTTATTGGGCGTAAAGCGAGCGCAGGCGGTCCGGTAAGTCTGATGTGAAAGCCCACGGCTCAACCGTGGAACGGCATTGGAAACTGGCGGACTTGAATGTAGCAGAGGAAAGTGGAATTCCATGTGTAGCGGTGGAATGCGTAGATATATGGAGGAACACCAGTGGCGAAAGCGACTTTCTGGGCTATGATTGACGCTGAGGCTCGAAAGCGTGGGGAGCGAACAGG
Alloprevotella 1 (Ihuprevotella)	massiliensis	66%	57%	70%	54%	1,7%	0,5%	1,3%	0,3%	TACGGAAGGTCCAGGCGTTATCCGGATTTATTGGGTTTAAAGGGAGCGTAGGCGGATTATTAAGTCAGTGGTGAAAGACGGTGGCTCAACCATCGTTAGCCATTGAAACTGGTAGTCTTGAGTGCAGACAGGGATGCTGGAACTCGTGGTGTAGCGGTGAAATGCTTAGATATCACGATGAACTCCGATCGCGAAGGCAGGTGTCCGGGCTGCAACTGACGCTGAGGCTCGAAAGTGTGGGTATCAAACAGG
Alloprevotella 10	rava	10%	4%	10%	2%	0,1%	0,1%	0,1%	0,0%	TACGGAAGGTCCAGGCGTTATCCGGATTTATTGGGTTTAAAGGGAGCGTAGGCGGACGATTAAGTCAGCTGTGAAAGTTTGCGGCTCAACCGTAAAATTGCAGTTGAAACTGGTTGTCTTGAGTGCACGCAGGGATGTTGGAATTCATGGTGTAGCGGTGAAATGCTTAGATATCATGAAGAACTCCGATCGCGAAGGCATATGTCCGGAGTGCAACTGACGCTGAGGCTCGAAAGTGCGGGTATCGAACAGG
Alloprevotella 2	NA	30%	8%	30%	2%	0,5%	0,4%	0,4%	0,1%	TACGGAAGGTCCAGGCGTTATCCGGATTTATTGGGTTTAAAGGGAGCGTAGGCGGATTGTTAAGTCAGCGGTTAAAGGGTGTGGCTCAACCATGCATTGCCGTTGAAACTGGCGATCTTGAGTGCAGACAGGGATGCCGGAATTCGTGGTGTAGCGGTGAAATGCTTAGATATCACGAAGAACTCCGATCGCGAAGGCAGGTGTCCGGGCTGCAACTGACGCTGAGGCTCGAAAGTGTGGGTATCAAACAGG
Alloprevotella 3	NA	30%	27%	40%	24%	0,4%	0,5%	1,7%	0,0%	TACGGAAGGTCCAGGCGTTATCCGGATTTATTGGGTTTAAAGGGAGCGTAGGCGGATTGTTAAGTCAGCGGTTAAAGGGTGTGGCTCAACCATACATTGCCGTTGAAACTGGCGATCTTGAGTGCAGACAGGGATGCCGGAATTCGTGGTGTAGCGGTGAAATGCTTAGATATCACGAAGAACTCCGATCGCGAAGGCAGGTGTCCGGGCTGCAACTGACGCTGAGGCTCGAAAGTGTGGGTATCAAACAGG
Alloprevotella 4	NA	10%	12%	10%	12%	0,2%	2,8%	16,2%	0,1%	TACGGAAGGTCCAGGCGTTATCCGGATTTATTGGGTTTAAAGGGAGCGCAGGCGGATTATTAAGTCAGTGGTGAAAGACGGTGGCTCAACCATCGTTAGCCATTGAAACTGGTAGTCTTGAGTGCAGACAGGGATGCTGGAACTCGTGGTGTAGCGGTGAAATGCTTAGATATCACGATGAACTCCGATCGCGAAGGCAGGTGTCCGGGCTGTAACTGACGCTGAGGCTCGAAAGTGTGGGTATCAAACAGG
Alloprevotella 7	NA	4%	4%	10%	2%	0,4%	0,1%	0,0%	0,2%	TACGGAAGGTCCGGGCGTTATCCGGAATTATTGGGTTTAAAGGGAGCGCAGGCGGGAGTGTAAGTCAGCTGTTAAATATCAGGGCCCAACTCTGTTATGCAGTTGAAACTATATTTCTTGAGTACGCACAGGGATGGCGGAATTCAGGGTGTAGCGGTGAAATGCTTAGATATCCTGAAGAACTCCGATCGCGAAGGCAGCCATCCGGAGCGTAACTGACGCTGAGGCTCGAAGGTGCGGGTATCAAACAGG
AM420062_g	s	3%	6%	10%	5%	0,1%	0,0%	0,1%	0,0%	TACGTAGGGGGCAAGCGTTATCCGGATTTACTGGGTGTAAAGGGAGCGCAGACGGCTTGGCAAGTCTGAAGTGAAAGCCCGGGGCTTAACCCCGGGACTGCTTTGGAAACTGTGAAGCTAGAGTGTCGGAGAGGTAAGTGGAATTCCCAGTGTAGCGGTGAAATGCGTAGATATTGGGAGGAACACCGGTGGCGAAGGCGGCTTACTGGACGATAACTGACGTTGAGGCTCGAAGGCGTGGGGAGCAAACAGG
Anaerococcus 1	murdochii/degeneri	3%	4%	10%	2%	0,1%	0,1%	0,2%	0,0%	TACGTAAGGTCCGAGCGTTGTCCGGAATCATTGGGCGTAAAGGGTACGTAGGCGGGTAAGCAAGTTAGAAGTGAAATCCTATAGCTCAACTATAGTAAGCTTTTAAAACTGCTCATCTTGAGGTATGGAAGGGAAAGTGGAATTCCTAGTGTAGCGGTGAAATGCGCAGATATTAGGAGGAATACCGGTGGCGAAGGCGACTTTCTGGCCATAACCTGACGCTGAGGTACGAAAGCGTGGGTAGCAAACAGG
Atopobium 1	parvulum	16%	14%	30%	10%	0,1%	0,0%	0,1%	0,0%	TACGTAGGGGGCAAGCGTTATCCGGATTCATTGGGCGTAAAGCGCTCGTAGGCGGTCTGTTAGGTCGGGAGTTAAATCCGGGGGCTCAACCCCCGTTCGCTCCCGATACCGGCAGACTTGAGTTTGGTAGGGGAAGGTGGAATTCCTAGTGTAGCGGTGGAATGCGCAGATATTAGGAAGAACACCAGTGGCGAAGGCGGCCTTCTGGGCCATAACTGACGCTGAGGAGCGAAAGCTAGGGGAGCAAACAGG

Taxon Name	Species	Prevalence DNP (n=67)	Prevalence HNP_{All} (n=51)	Prevalence HNP_{CI} n=10	Prevalence HNP_{DC} (n=41)	Mean Relative Abundance DNP (n=67)	Mean Relative Abundance HNP_{All} (n=51)	Mean Relative Abundance HNP_{CI} (n=10)	Mean Relative Abundance HNP_{DC} (n=41)	Sequence
Bergeyella 1	NA	36%	29%	10%	34%	0,3%	0,1%	0,2%	0,1%	TACGGAGGGTGCAAGCGTTATCCGGACTTATTGGGTTTAAAGGGTCCGTAGGCGGGTTATTAAGTCAGTGGTGAAATCTCATAGCTCAACTATGAAACTGCCATTGATACTGATAGTCTTGAGTATATTTGAAGTAGCTGGAATAAGTAGTGTAGCGGTGAAATGCATAGATATTACTTAGAACACCAATAGCGAAGGCAGGTTACTAAGATATTACTGACGCTGAGGGACGAGAGCGTAGGGAGCGAACAGG
Bergeyella 2	NA	12%	12%	20%	10%	0,1%	0,2%	0,5%	0,0%	TACGGAGGGTGCAAGCGTTATCCGGATTTATTGGGTTTAAAGGGTCCGTAGGTGGGCTGATAAGTCAGCGGTGAAATCCTGCAGCTTAACTGTAGAACTGCCGTTGATACTGTTAGTCTTGAGTGTATTTGAAGTGGCTGGAATAAGTAGTGTAGCGGTGAAATGCATAGATATTACTTAGAACACCAATTGCGAAGGCAGGTCACTAAGATACAACTGACGCTGAGGGACGAAAGCGTGGGGAGCGAACAGG
Bergeyella 3	NA	16%	10%	20%	7%	0,1%	0,1%	0,1%	0,0%	TACGGAGGGTGCAAGCGTTATCCGGATTTATTGGGTTTAAAGGGTCCGTAGGTGGGTTAGTAAGTCAGTGGTGAAATCCTGCAGCTTAACTGTAGAATTGCCATTGATACTGCTAGTCTTGAGTGTATTTGAAGTAGCTGGAATAAGTAGTGTAGCGGTGAAATGCATAGATATTACTTAGAACACCAATTGCGAAGGCAGGTTACTAAGATACAACTGACGCTGATGGACGAAAGCGTGGGGAGCGAACAGG
Blautia 1	wexlerae	1%	4%	10%	2%	0,0%	0,0%	0,0%	0,0%	TACGTAGGGGGCAAGCGTTATCCGGATTTACTGGGTGTAAAGGGAGCGTAGACGGTGTGGCAAGTCTGATGTGAAAGGCATGGGCTCAACCTGTGGACTGCATTGGAAACTGTCATACTTGAGTGCCGGAGGGGTAAGCGGAATTCCTAGTGTAGCGGTGAAATGCGTAGATATTAGGAGGAACACCAGTGGCGAAGGCGGCTTACTGGACGGTAACTGACGTTGAGGCTCGAAAGCGTGGGGAGCAAACAGG
Bulleidia 1	NA	9%	14%	30%	10%	0,1%	0,0%	0,1%	0,0%	TACGTAGGTGGCGAGCGTTATCCGGAATTATTGGGCGTAAAGGGTGCGTAGGCGGCCTGTTAAGTAAGTGGTTAAATTGTTGGGCTCAACCCAATCCAGCCACTTAAACTGGCAGGCTAGAGTATTGGAGAGGCAAGTGGAATTCCATGTGTAGCGGTAAAATGCGTAGATATATGGAGGAACACCAGTGGCGAAGGCGGCTTGCTAGCCAAAGACTGACGCTCATGCACGAAAGCGTGGGGAGCAAATAGG
Campylobacter 2	concisus	31%	22%	40%	17%	0,1%	0,1%	0,1%	0,0%	TACGGAGGGTGCAAGCGTTACTCGGAATCACTGGGCGTAAAGGACGCGTAGGCGGATTATCAAGTCTCTTGTGAAATCCTATGGCTTAACCATAGAACTGCTTGGGAAACTGATAATCTAGAGTGAGGGAGAGGCAGATGGAATTGGTGGTGTAGGGGTAAAATCCGTAGAGATCACCAGGAATACCCATTGCGAAGGCGATCTGCTGGAACTCAACTGACGCTAATGCGTGAAAGCGTGGGGAGCAAACAGG
Capnocytophaga 1	leadbetteri	30%	14%	10%	15%	0,1%	0,2%	0,0%	0,2%	TACGGAGGATGCGAGCGTTATCCGGAATCATTGGGTTTAAAGGGTCCGTAGGCGGGCTAATAAGTCAGAGGTGAAAGCGCTCAGCTCAACTGAGCAACTGCCTTTGAAACTGTTAGTCTTGAATGGTTGTGAAGTAGTTGGAATGTGTAGTGTAGCGGTGAAATGCTTAGATATTACACAGAACACCGATAGCGAAGGCATATTACTAACAATTAATTGACGCTGATGGACGAAAGCGTGGGGAGCGAACAGG
Capnocytophaga 3	sputigena	24%	18%	20%	17%	0,1%	0,1%	0,1%	0,1%	TACGGAGGATGCGAGCGTTATCCGGAATCATTGGGTTTAAAGGGTCCGTAGGCGGGCTGATAAGTCAGAGGTGAAAGCGCTTAGCTCAACTAAGCAACTGCCTTTGAAACTGTCAGTCTTGAATGATTGTGAAGTAGTTGGAATGTGTAGTGTAGCGGTGAAATGCTTAGATATTACACAGAACACCGATAGCGAAGGCATATTACTAACAATTTATTGACGCTGATGGACGAAAGCGTGGGGAGCGAACAGG
Capnocytophaga 5	NA	12%	8%	20%	5%	0,1%	0,0%	0,1%	0,0%	TACGGAGGATGCGAGCGTTATTCGGAATCATTGGGTTTAAAGGGTCTGTAGGCGGGCTATTAAGTCAGGGGTGAAAGGTTTCAGCTTAACTGAGAAATTGCCTTTGATACTGGTAGTCTTGAATATCTGTGAAGTTCTTGGAATGTGTAGTGTAGCGGTGAAATGCTTAGATATTACACAGAACACCGATTGCGAAGGCAGGGGACTAACAGACAATTGACGCTGAGAGACGAAAGCGTGGGGAGCGAACAGG
Conchiformibius 1 (Eikenella)	corrodens	4%	6%	20%	2%	0,2%	0,5%	0,7%	0,1%	TACGTAGGGTGCGAGCGTTAATCGGAATTACTGGGCGTAAAGCGAGCGCAGACGGTTATTTAAGCAGGATGTGAAATCCCCGGGCTTAACCTGGGAACTGCGTTCTGAACTGGATAGCTAGAGTGTGTCAGAGGGGGGTAGAATTCCACGTGTAGCAGTGAAATGCGTAGAGATGTGGAGGAATACCGATGGCGAAGGCAGCCCCCTGGGATAACACTGACGTTCATGCTCGAAAGCGTGGGTAGCAAACAGG
Corynebacterium 1	propinquum/pseudodiphtheriticum	60%	63%	50%	66%	2,3%	2,5%	6,3%	1,8%	TACGTAGGGTGCAAGCGTTGTCCGGAATTACTGGGCGTAAAGAGCTCGTAGGTGGTTTGTCACGTCGTCTGTGAAATTCCACAGCTTAACTGTGGGCGTGCAGGCGATACGGGCTGACTTGAGTACTGTAGGGGTAACTGGAATTCCTGGTGTAGCGGTGAAATGCGCAGATATCAGGAGGAACACCGATGGCGAAGGCAGGTTACTGGGCAGTTACTGACGCTGAGGAGCGAAAGCATGGGTAGCAAACAGG

Taxon Name	Species	Prevalence DNP (n=67)	Prevalence HNP_{All} (n=51)	Prevalence HNP_{CI} n=10	Prevalence HNP_{DC} (n=41)	Mean Relative Abundance DNP (n=67)	Mean Relative Abundance HNP_{All} (n=51)	Mean Relative Abundance HNP_{CI} (n=10)	Mean Relative Abundance HNP_{DC} (n=41)	Sequence
Corynebacterium 10	matruchotii	12%	14%	10%	15%	0,2%	0,2%	0,2%	0,2%	TACGTAGGGTGCGAGCGTTGTCCGGAATTACTGGGCGTAAAGAGCTCGTAGGTGGTTTGTTGCGTCGTCTGTGAAATTCCGGGGCTTAACTTCGGGGTGGCAGGCGATACGGGCATAACTAGAGTGCTGTAGGGGAGACTGGAATTCCTGGTGTAGCGGTGGAATGCGCAGATATCAGGAGGAACACCGATGGCGAAGGCAGGTCTCTGGGCAGTAACTGACGCTGAGGAGCGAAAGCATGGGGAGCGAACAGG
Corynebacterium 3	tuberculostearicum	4%	18%	10%	20%	0,5%	2,0%	0,3%	2,3%	TACGTAGGGTGCGAGCGTTGTCCGGAATTACTGGGCGTAAAGGGCTCGTAGGTGGTTTGTCGCGTCGTCTGTGAAATTCCGGGGCTTAACTCCGGGCGTGCAGGCGATACGGGCATAACTTGAGTACTGTAGGGGTAACTGGAATTCCTGGTGTAGCGGTGAAATGCGCAGATATCAGGAGGAACACCGATGGCGAAGGCAGGTTACTGGGCAGTTACTGACGCTGAGGAGCGAAAGCATGGGTAGCGAACAGG
Corynebacterium 9	durum	10%	14%	10%	15%	0,2%	0,2%	0,0%	0,2%	TACGTAGGGTGCAAGCGTTGTCCGGATTTACTGGGCGTAAAGAGCTCGTAGGTGGTGTGTCGCGTCGTCTGTGAAATTCCGGGGCTTAACTCCGGGCGTGCAGGCGATACGGGCACGACTAGAGTGCTGTAGGGGTAACTGGAATTCCTGGTGTAGCGGTGAAATGCGCAGATATCAGGAGGAACACCGATGGCGAAGGCAGGTTACTGGGCAGTTACTGACGCTGAGGAGCGAAAGCATGGGGAGCGAACAGG
CP009312_g (Lawsonella)	clevelandensis	3%	6%	20%	2%	0,2%	0,0%	0,0%	0,0%	TACGTAGGGTGCGAGCGTTGTCCGGAATTACTGGGCGTAAAGAGCTCGTAGGCGGTTTGTCACGTCGTCTGTGAAATCCTAGGGCTTAACCCTGGACGTGCAGGCGATACGGGCTGACTTGAGTACTACAGGGGAGACTGGAATTTCTGGTGTAGCGGTGGAATGCACAGATATCAGGAAGAACACCGATGGCGAAGGCAGGTCTCTGGGTAGTAACTGACGCTGAGGAGCGAAAGCATGGGTAGCGAACAGG
Dolosigranulum 1	pigrum	70%	71%	60%	73%	3,8%	5,5%	11,9%	4,2%	TACGTAGGTGACAAGCGTTGTCCGGATTTATTGGGCGTAAAGGGAGCGCAGGCGGTCTGTTTAGTCTAATGTGAAAGCCCACGGCTTAACCGTGGAACGGCATTGGAAACTGACAGACTTGAATGTAGAAGAGGAAAATGGAATTCCAAGTGTAGCGGTGGAATGCGTAGATATTTGGAGGAACACCAGTGGCGAAGGCGATTTTCTGGTCTAACATTGACGCTGAGGCTCGAAAGCGTGGGGAGCGAACAGG
Eubacterium_g10 1	sulci	6%	4%	10%	2%	0,0%	0,0%	0,0%	0,0%	TACGTAGGGGGCGAGCGTTATCCGGAATTATTGGGCGTAAAGAGTGCGTAGGTGGCACCTTAAGCGCAGGGTTTAAGGCAATGGCTCAACCATTGTTCGCCTTGCGAACTGGGGTGCTTGAGTGCAGGAGGGGAAAGTGGAATTCCTAGTGTAGCGGTGAAATGCGTAGATATTAGGAGGAACACCAGTGGCGAAGGCGACTTTCTGGACTGTTACTGACACTGAGGCACGAAAGCGTGGGGAGCAAACAGG
Eubacterium_g11	brachy	6%	8%	30%	2%	0,0%	0,1%	0,2%	0,1%	TACGGAGGGGGCAAGCGTTATCCGGAATTATTGGGCGTAAAGGGTACGTAGGTGGCCTTTTAAGCGTAGGGTATAAGGCAATGGCTCAACCATTGTTCGCCCTATGAACTGGAAGGCTTGAGTGCAGGAGAGGAAAGCGGAATTCCTAGTGTAGCGGTGGAATGCATAGATATTAGGAGGAACATCAGCGGCGAAGGCGGCTTTCTGGACTGCAACTGACACTGAGGTACGAAAGCGTGGGGAGCAAACAGG
Finegoldia	magna	4%	8%	10%	7%	0,1%	0,1%	0,1%	0,0%	TACGTATGGAGCGAGCGTTGTCCGGAATTATTGGGCGTAAAGGGTACGCAGGCGGTTTAATAAGTCGAATGTTAAAGATCGGGGCTCAACCCCGTAAAGCATTGGAAACTGATAAACTTGAGTAGTGGAGAGGAAAGTGGAATTCCTAGTGTAGTGGTGAAATACGTAGATATTAGGAGGAATACCAGTAGCGAAGGCGACTTTCTGGACACAAACTGACGCTGAGGTACGAAAGCGTGGGGAGCAAACAGG
FJ705147_g 1	NA	37%	18%	40%	12%	0,3%	1,4%	2,9%	0,1%	TACGTAGGGGGCAAGCGTTATCCGGATTTACTGGGTGTAAAGGGAGCGTAGACGGCATAGCAAGTCTGAAGTGAAAGCCCGGGGCTCAACCCCGGAACTGCTTTGGAAACTGTTAAGCTAGAGTGTCGGAGAGGTAAGTGGAATTCCCAGTGTAGCGGTGAAATGCGTAGATATTGGGAGGAACACCGGTGGCGAAGGCGGCTTACTGGACGATAACTGACGTTGAGGCTCGAAGGCGTGGGGAGCAAACAGG
FM873692_g 1	s	18%	35%	40%	34%	0,9%	0,3%	0,5%	0,2%	TACGTAGGGTGCAAGCGTTAATCGGAATTATTGGGCGTAAAGCGAGTGCAGACGGTTACTTAAGCCAGATGTGAAATCCCCAAGCTTAACTTGGGACGTGCATTTGGAACTGGGTGACTAGAGTGTGTCAGAGGGAGGTAGAATTCCACATGTAGCGGTGGAATGCGTAGAGATGTGGAGGAATACCGATGGCGAAGGCAGCCTCCTGGGATAACACTGACGTTGAGGCTCGAAAGCGTGGGGAGCAAACAGG
FM873692_g 2	NA	1%	12%	20%	10%	0,1%	0,3%	0,8%	0,0%	TACGTAGGGTGCGAGCGTTAATCGGAATTATTGGGCGTAAAGCGAGTGTAGACGGTTATTTAAGCCAGATGTGAAATACCCGAGCCTAACTTGGGAGGTGCATATGGAACTGGGTAGCTAGAGTGTGTCAGAGGGAGGTAGAACTCCACGTGTAGCAGTGAAATGCGTAGAGATGTGGAAGAATACCGATGGCGAAGGCAGCCTCCTGGGATAACACTGACGTTGAGGCTCGAAAGCGTGGGGAGCAAACAGG

		Prevalence				Mean Relative Abundance				
Taxon Name	Species	DNP (n=67)	HNP_{All} (n=51)	HNP_{CI} n=10	HNP_{DC} (n=41)	DNP (n=67)	HNP_{All} (n=51)	HNP_{CI} (n=10)	HNP_{DC} (n=41)	Sequence
FM873692_g 3	NA	1%	6%	10%	5%	0,1%	0,0%	0,0%	0,0%	TACGTAGGGTGCAAGCGTTAATCGGAATTATTGGGCGTAAAGCGAGTGCAGACGGTTACTTAAGCCAGATGTGAAAT CCCCAAGCTTAACTTGGGACGTGCATTTGGAACTGGGTAACTAGAGTGTGTCAGAGGGAGGTAGAATTCCACATGTA GCGGTGGAATGCGTAGAGATGTGGAGGAATACCGATGGCGAAGGCAGCCTCCTGGGATAACACTGACGTTGAGGCT CGAAAGCGTGGGGAGCAAACAGG
Fusobacterium 1	necrophorum subsp. necrophorum/funduliforme	21%	20%	40%	15%	13,5%	2,6%	4,7%	1,2%	TACGTATGTCGCAAGCGTTATCCGGATTTATTGGGCGTAAAGCGCGTCTAGGCGGCAAGGAAAGTCTGATGTGAAAA TGCGGAGCTCAACTCCGTATGGCGTTGGAAACTGCCTTACTAGAGTACTGGAGAGGTAGGCGGAACTACAAGTGTAG AGGTGAAATTCGTAGATATTTGTAGGAATGCCGATGGGGAAGCCAGCCTACTGGACAGATACTGACGCTAAAGCGCG AAAGCGTGGGTAGCAAACAGG
Fusobacterium 3	periodonticum	37%	29%	40%	27%	0,7%	0,3%	0,6%	0,2%	TACGTATGTCACAAGCGTTATCCGGATTTATTGGGCGTAAAGCGCGTCTAGGTGGTTATGTAAGTCTGATGTGAAAAT GCAGGGCTCAACTCTGTATTGCGTTGGAAACTGCATGACTAGAGTACTGGAGAGGTAAGCGGAACTACAAGTGTAGA GGTGAAATTCGTAGATATTTGTAGGAATGCCGATGGGGAAGCCAGCTTACTGGACAGATACTGACGCTAAAGCGCGA AAGCGTGGGTAGCAAACAGG
Fusobacterium 5	massiliense	46%	14%	30%	10%	0,3%	0,3%	0,6%	0,1%	TACGTATGTCACAAGCGTTATCCGGATTTATTGGGCGTAAAGCGCGTCTAGGCGGCTATGTAAGTCTGATGTGAAAAT GCGGGGCTCAACTCCGTATTGCGTTGGAAACTGTGTAGCTAGAGTATCGGAGAGGTAAGCGGAACTACAAGTGTAGA GGTGAAATTCGTAGATATTTGTAGGAATGCCGATGGGGAAGCCAGCTTACTGGACGAATACTGACGCTAAAGCGCGA AAGCGTGGGTAGCAAACAGG
Fusobacterium 6	NA	33%	20%	20%	20%	0,2%	0,3%	1,2%	0,1%	TACGTATGTCACAAGCGTTATCCGGATTTATTGGGCGTAAAGCGCGTCTAGGCGGTTAATTAAGTCTGATGTGAAAAT GCGGAGCTCAACTCCGTATTGCGTTGGAAACTGGTTAACTAGAGTACTGGAGAGGTAAGCGGAACTACAAGTGTAGA GGTGAAATTCGTAGATATTTGTAGGAATGCCGATGGGGAAGCCAGCTTACTGGACAGATACTGACGCTAAAGCGCGA AAGCGTGGGTAGCAAACAGG
Fusobacterium 8	canifelinum	16%	10%	20%	7%	0,4%	0,1%	0,2%	0,1%	TACGTATGTCACAAGCGTTATCCGGATTTATTGGGCGTAAAGCGCGTCTAGGTGGTTATGTAAGTCTGATGTGAAAAT GCAGGGCTCAACTCTGTATTGCGTTGGAAACTGTGTAACTAGAGTACTGGAGAGGTAAGCGGAACTACAAGTGTAGA GGTGAAATTCGTAGATATTTGTAGGAATGCCGATGGGGAAGCCAGCTTACTGGACAGATACTGACGCTGAAGCGCGA AAGCGTGGGTAGCAAACAGG
Gemella 1	haemolysans/morbillorum/sanguinis/taiwanensis/parahaemolysans	84%	69%	70%	68%	0,7%	1,1%	3,2%	0,5%	TACGTAGGTGGCAAGCGTTGTCCGGAATTATTGGGCGTAAAGCGCGCGCAGGTGGTTTAATAAGTCTGATGTGAAAG CCCACGGCTCAACCGTGGAGGGTCATTGGAAACTGTTAAACTTGAGTGCAGGAGAGAAAAGTGGAATTCCTAGTGTA GCGGTGAAATGCGTAGAGATTAGGAGGAACACCAGTGGCGAAGGCGGCTTTTTGGCCTGTAACTGACACTGAGGCG CGAAAGCGTGGGGAGCAAACAGG
GQ422727_g 1	s	15%	10%	30%	5%	0,2%	0,9%	1,5%	0,0%	AACATAGGGGGCAAGCGTTGTCCGGAATCACTGGGCGTAAAGGGCGCGCAGGCGGTAAATTAAGTCAGGTGTGAAA GTTCGGGGCTCAACCCCGTGATTGCACCTGATACTGATAAACTAGAGTGTTGGAGAGGTAAGTGGAATTCCTAGTGTA GCGGTGGAATGCGTAGATATTAGGAGGAACACCAGTGGCGAAGGCGACTTACTGGCCAATAACTGACGCTGAGGCG CGAAAGCGTGGGGAGCAAACAGG
Granulicatella 1	elegans	70%	53%	60%	51%	0,5%	0,6%	0,9%	0,5%	TACGTAGGTGGCAAGCGTTGTCCGGATTTATTGGGCGTAAAGCGAGCGCAGGCGGTCAATTAAGTCTGATGTGAAAG CCCCCGGCTCAACCGGGGAGGGTCATTGGAAACTGGTTGACTTGAGTGCAGAAGAGGAGAGTGGAATTCCATGTGTA GCGGTGAAATGCGTAGATATATGGAGGAACACCAGTGGCGAAGGCGACTCTCTGGTCTGTAACTGACGCTGAGGCTC GAAAGCGTGGGTAGCAAACAGG
Granulicatella 2	adiacens	39%	43%	50%	41%	0,2%	0,4%	0,2%	0,5%	TACGTAGGTGGCAAGCGTTGTCCGGATTTATTGGGCGTAAAGCGAGCGCAGGCGGTTCCTTAAGTCTGATGTGAAAG CCCCCGGCTCAACCGGGGAGGGTCATTGGAAACTGGGGAACTTGAGTGCAGAAGAGGAGAGTGGAATTCCATGTGT AGCGGTGAAATGCGTAGATATATGGAGGAACACCAGTGGCGAAGGCGACTCTCTGGTCTGTAACTGACGCTGAGGCT CGAAAGCGTGGGTAGCAAACAGG
GU410548_g 1	NA	4%	6%	10%	5%	0,1%	0,1%	0,1%	0,0%	TACGTAGGTCTCAAGCGTTATCCGGATTTACTGGGCGTAAAGTGTCCGTAGTCTGATCTGTAAGTCTGTTTTCAAATCC TACGACTCAATCGTAGAAAGGGAATGGATACTGCAGATCTAGAAGTATCTAGGGGTTAGTGGAATTTCCGGTGGAGC GGTGAAATGCGTTGATATCGGAAGGAACGCCGAAAGCGAAAGCAGCTAACTATAGAATACTTGACGATGAGGGACG ACAGTTTGGGGAGCAAACAGG

		Prevalence				Mean Relative Abundance				
**Taxon Name**	**Species**	**DNP (n=67)**	**HNP_{All} (n=51)**	**HNP_{CI} n=10**	**HNP_{DC} (n=41)**	**DNP (n=67)**	**HNP_{All} (n=51)**	**HNP_{CI} (n=10)**	**HNP_{DC} (n=41)**	**Sequence**
GU410548_g 2	NA	21%	6%	10%	5%	0,1%	0,0%	0,1%	0,0%	TACGTAGGTCTCAAGCGTTATCCGGATTTACTGGGCGTAAAGTGTCCGTAGTCTGAATTGTAAGTCTGTTTTCAAATCCTACGACTCAATCGTAGAAAGGGAGTGGATACTGCAATTCTAGAAGTATCTAGGGGTTAGTGGAATTTCCGGTGGAGCGGTGAAATGCGTTGATATCGGAAGGAACGCCGAAAGCGAAAGCAGCTAACTATAGAATACTTGACGATGAGGGACGACAGTTTGGGGAGCAAACAGG
Haemophilus 1	influenzae/seminalis	90%	82%	70%	85%	17,2%	14,2%	6,2%	15,8%	TACGGAGGGTGCGAGCGTTAATCGGAATAACTGGGCGTAAAGGGCACGCAGGCGGTTATTTAAGTGAGGTGTGAAAGCCCCGGGCTTAACCTGGGAATTGCATTTCAGACTGGGTAACTAGAGTACTTTAGGGAGGGGTAGAATTCCACGTGTAGCGGTGAAATGCGTAGAGATGTGGAGGAATACCGAAGGCGAAGGCAGCCCCTTGGGAATGTACTGACGCTCATGTGCGAAAGCGTGGGGAGCAAACAGG
Haemophilus 2	aegyptius	67%	71%	70%	71%	27,8%	16,2%	18,3%	15,7%	TACGGAGGGTGCGAGCGTTAATCGGAATAACTGGGCGTAAAGGGCACGCAGGCGGTTATTTAAGTGAGGTGTGAAAGCCCTGGGCTTAACCTAGGAATTGCATTTCAGACTGGGTAACTAGAGTACTTTAGGGAGGGGTAGAATTCCACGTGTAGCGGTGAAATGCGTAGAGATGTGGAGGAATACCGAAGGCGAAGGCAGCCCCTTGGGAATGTACTGACGCTCATGTGCGAAAGCGTGGGGAGCAAACAGG
Haemophilus 3	quentini/influenzae	34%	14%	10%	15%	6,6%	12,0%	2,7%	13,6%	TACGGAGGGTGCGAGCGTTAATCGGAATAACTGGGCGTAAAGGGCACGCAGGCGGTTATTTAAGTGAGGTGTGAAAGCCCTGGGCTTAACCTGGGAATTGCATTTCAGACTGGGTAACTAGAGTACTTTAGGGAGGGGTAGAATTCCACGTGTAGCGGTGAAATGCGTAGAGATGTGGAGGAATACCGAAGGCGAAGGCAGCCCCTTGGGAATGTACTGACGCTCATGTGCGAAAGCGTGGGGAGCAAACAGG
JN713389_g 1	NA	16%	6%	10%	5%	0,1%	0,1%	0,0%	0,1%	TACATATGGGGCGAGCGTTATCCGGAATTATTGGGTGTAAAGGGTGCGTAGGCGGAATAGCAAGTCAGTATGTGAAATCTCTCGGCTCAACCGAGAAACTGCGACTGAAACTACTATTCTTGAGTGTTGGAGGGGAAAGTGGAATTCCTAGTGTAGCGGTGAAATGCGTAGATATTAGGAGGAACACCAGTGGCGAAGGCGACTTTCTGGACAACAACTGACGCTGAGGCACGAAAGCGTGGGTAGCAAACAGG
Lachnoanaerobaculum 1	gingivalis	34%	16%	20%	15%	0,1%	0,1%	0,1%	0,1%	TACGTAGGGGGCAAGCGTTATCCGGATTTACTGGGTGTAAAGGGAGCGCAGACGGCAATGCAAGTCTGAAGTGAAAGGCGTGGGCTCAACCCATGAACTGCTTTGGAAACTGTATAGCTTGAGTGTCGGAGGGGTAAGCGGAATTCCTAGTGTAGCGGTGAAATGCGTAGATATTAGGAGGAACACCGGAGGCGAAGGCGGCTTACTGGACGACAACTGACGTTGAGGCTCGAAGGCGTGGGGAGCAAACAGG
Lachnoanaerobaculum 2	orale	13%	8%	20%	5%	0,1%	0,0%	0,1%	0,0%	TACGTAGGGGGCAAGCGTTATCCGGATTTACTGGGTGTAAAGGGAGCGCAGACGGCGAAGCAAGTCTGAAGTGAAATGCATGGGCTCAACCCATGAATTGCTTTGGAAACTGTTTGGCTTGAGTGTCGGAGGGGTAAGCGGAATTCCTAGTGTAGCGGTGAAATGCGTAGATATTAGGAGGAACACCGGAGGCGAAGGCGGCTTACTGGACGACAACTGACGTTGAGGCTCGAAGGCGTGGGGAGCAAACAGG
Lactobacillus 3	iners	1%	4%	10%	2%	0,0%	0,0%	0,0%	0,0%	TACGTAGGTGGCAAGCGTTGTCCGGATTTATTGGGCGTAAAGCGAGTGCAGGCGGCTCGATAAGTCTGATGTGAAAGCCTTCGGCTCAACCGGAGAATTGCATCAGAAACTGTCGAGCTTGAGTACAGAAGAGGAGAGTGGAACTCCATGTGTAGCGGTGAAATGCGTAGATATATGGAAGAACACCGGTGGCGAAGGCGGCTCTCTGGTCTGTTACTGACGCTGAGGCTCGAAAGCATGGGTAGCGAACAGG
Lautropia 1	mirabilis	21%	27%	40%	24%	0,1%	0,1%	0,1%	0,1%	TACGTAGGGTGCAAGCGTTAATCGGAATTACTGGGCGTAAAGAGTGCGCAGGCGGTTTTGCAAGACCGATGTGAAATCCCCGGGCTTAACCTGGGAACTGCATTGGTGACTGCAAGGCTAGAGTGTGTCAGAGGGAGGTGGAATTCCGCATGTAGCAGTGAAATGCGTAGATATGCGGAGGAACACCGATGGCGAAGGCAGCCTCCTGGGATAACACTGACGCTCATGCACGAAAGCGTGGGGAGCAAACAGG
Leptotrichia 1	NA	22%	10%	20%	7%	0,8%	0,0%	0,0%	0,0%	TACGTATGTCGCGAGCGTTATCCGGAATTATTGGGCATAAAGGGCATCTAGGCGGTTATGCAAGTCAGGGGTGAAAACTTACGGCTCAACCGTGAGCTTGCCTTTGAAACTGTGTGACTAGAGTACTGGAGAGGTGGGCGGAACTACACGAGTAGAGGTGAAATTCGTAGATATGTGTAGGAATGCCGATGATGAAGATAGCTCACTGGACAGAAACTGACGCTGAAGTGCGAAAGCTAGGGGAGCGAACAGG
Leptotrichia 12	NA	7%	6%	20%	2%	0,1%	0,1%	0,1%	0,1%	TACGTATGTCGCGAGCGTTATCCGGAATTATTGGGCATAAAGGGCATCTAGGCGGATATACAAGTCAGGGGTGAAAACTTAGGGCTCAACTCAAAGCTTGCCTTTGAAACTGTATATCTAGAGTGCTGGAGAGGTGGACGGAACTACACGAGTAGAGGTGAAATTCGTAGATATGTGTAGGAATGCCGATGATGAAGATAGTCCACTGGACAGCAACTGACGCTGAAGTGCGAAAGCTAGGGGAGCAAACAGG

Taxon Name	Species	Prevalence DNP (n=67)	Prevalence HNP_{All} (n=51)	Prevalence HNP_{CI} n=10	Prevalence HNP_{DC} (n=41)	Mean Relative Abundance DNP (n=67)	Mean Relative Abundance HNP_{All} (n=51)	Mean Relative Abundance HNP_{CI} (n=10)	Mean Relative Abundance HNP_{DC} (n=41)	Sequence
Leptotrichia 2	wadei	10%	10%	10%	10%	0,2%	0,2%	0,6%	0,1%	TACGTATGTCGCAAGCGTTATCCGGAATTATTGGGCATAAAGGGCATCTAGGCGGCCAGATAAGTCTGGGGTGAAAACTTGCGGCTCAACCGCAAGCCTGCCCTGGAAACTATGTGGCTAGAGTACTGGAGAGGTGGACGGAACTGCACGAGTAGAGGTGAAATTCGTAGATATGTGCAGGAATGCCGATGATGAAGATAGTTCACTGGACGGTAACTGACGCTGAAGTGCGAAAGCTAGGGGAGCAAACAGG
Leptotrichia 3	NA	19%	8%	20%	5%	0,3%	0,3%	0,4%	0,3%	TACGTATGTCGCGAGCGTTATCCGGAATTATTGGGCATAAAGGGCATCTAGGCGGCCTTTCAAGTCAGGGGTGAAAACCTGCGGCTCAACCGCAGGCCTGCCTTTGAAACTGATAGGCTGGAGTACCGGAGAGGTGGACGGAACTGCACGAGTAGAGGTGAAATTCGTAGATATGTGCAGGAATGCCGATGATGAAGATAGTTCACTGGACGGTAACTGACGCTGAAGTGCGAAAGCCGGGGGAGCGAACAGG
Leptotrichia 4	NA	9%	6%	10%	5%	0,1%	0,1%	0,1%	0,1%	TACGTATGTCGCGAGCGTTATCCGGAATTATTGGGCATAAAGGGCATCTAGGCGGCCTGACAAGTCAGGGGTGAAAACCTGCGGCTCAACCGCAGGCCTGCCTTTGAAACTGTGAGGCTGGAGTACCGGAGAGGTGGACGGAACTGCACGAGTAGAGGTGAAATTCGTAGATATGTGCAGGAATGCCGATGATGAAGATAGTTCACTGGACGGTAACTGACGCTGAAGTGCGAAAGCCGGGGGAGCGAACAGG
Megasphaera 1	micronuciformis	24%	10%	20%	7%	0,1%	0,1%	0,2%	0,1%	TACGTAGGTGGCAAGCGTTGTCCGGAATTATTGGGCGTAAAGGGCGCGCAGGCGGCTTCTTAAGTCTGTCTTAAAAGTGCGGGGCTTAACCCCGTGATGGGATGGAAACTGGGAAGCTCAGAGTATCGGAGAGGAAAGCGGAATTCCTAGTGTAGCGGTGAAATGCGTAGATATTAGGAGGAACACCAGTGGCGAAAGCGGCTTTCTGGACGAAAACTGACGCTGAGGCGCGAAAGCCAGGGGAGCGAACGGG
Moraxella 1	catarrhalis/nonliquefaciens	84%	92%	80%	95%	25,1%	29,1%	18,7%	31,3%	TACAGAGGGTGCAAGCGTTAATCGGAATTACTGGGCGTAAAGCGCGCGTAGGTGGTTATTTAAGTCAGATGTGAAAGCCCCGGGCTTAACCTGGGAACTGCATCTGATACTGGATAACTAGAGTAGGTGAGAGGGGAGTAGAATTCCAGGTGTAGCGGTGAAATGCGTAGAGATCTGGAGGAATACCGATGGCGAAGGCAGCTCCCTGGCATCATACTGACACTGAGGTGCGAAAGCGTGGGTAGCAAACAGG
Moraxella 2	lincolnii	22%	41%	10%	49%	2,9%	16,7%	7,0%	17,2%	TACAGAGGGTGCAAGCGTTAATCGGAATTACTGGGCGTAAAGCGAGCGTAGGTGGTTTATTAAGTCAGATGTGAAATCCCTGGGCTTAACCTAGGAACTGCATCTGATACTGATAAACTAGAGTAGGTGAGAGGAAAGTAGAATTCCAGGTGTAGCGGTGAAATGCGTAGAGATCTGGAGGAATACCGATGGCGAAGGCAGCTTTCTGGCATCATACTGACACTGAGGTTCGAAAGCGTGGGTAGCAAACAGG
Moryella 1	NA	13%	8%	20%	5%	0,1%	0,1%	0,0%	0,1%	TACGTAGGGGGCAAGCGTTATCCGGATTTACTGGGTGTAAAGGGAGCGCAGACGGTTGCGCAAGTCTGAAGTGAAATCCCGAGGCTTAACCACGGGACTGCTTTGGAAACTGTGCGACTTGAGTATCGGAGGGGCAGGCGGAATTCCTAGTGTAGCGGTGAAATGCGTAGATATTAGGAAGAACACCGGTGGCGAAGGCGGCCTGCTGGACGAAAACTGACGTTGAGGCTCGAAGGCGTGGGGAGCAAACAGG
Neisseria 1	cinerea/perflava/subflava	61%	59%	60%	59%	3,7%	1,6%	4,1%	0,9%	TACGTAGGGTGCGAGCGTTAATCGGAATTACTGGGCGTAAAGCGAGCGCAGACGGTTACTTAAGCAGGATGTGAAATCCCCGGGCTCAACCTGGGAACTGCGTTCTGAACTGGGTGACTAGAGTGTGTCAGAGGGAGGTAGAATTCCACGTGTAGCAGTGAAATGCGTAGAGATGTGGAGGAATACCGATGGCGAAGGCAGCCTCCTGGGATAACACTGACGTTCATGCTCGAAAGCGTGGGTAGCAAACAGG
Neisseria 11	polysaccharea	3%	4%	10%	2%	1,2%	0,6%	0,3%	1,0%	TACGTAGGGTGCGAGCGTTAATCGGAATTACTGGGCGTAAAGCGGGCGCAGACGGTTACTTAAGCAGGATGTGAAATCCCCGGGCTCAACCCGGGAACTGCGTTCTGAACTGGGTGACTCGAGTGTGTCAGAGGGAGGTAGAATTCCACGTGTAGCAGTGAAATGCGTAGAGATGTGGAGGAATACCGATGGCGAAGGCAGCCTCCTGGGATAACACTGACGTTCATGCCCGAAAGCGTGGGTAGCAAACAGG
Neisseria 3 (Neisseria or Morococcus)	N. mucosa/macacae/flava or M. cerebrosus	39%	31%	60%	24%	1,9%	0,7%	0,7%	0,8%	TACGTAGGGTGCGAGCGTTAATCGGAATTACTGGGCGTAAAGCGGGCGCAGACGGTTACTTAAGCAGGATGTGAAATCCCCGGGCTCAACCTGGGAACTGCGTTCTGAACTGGGTGACTAGAGTGTGTCAGAGGGAGGTAGAATTCCACGTGTAGCAGTGAAATGCGTAGAGATGTGGAGGAATACCGATGGCGAAGGCAGCCTCCTGGGATAACACTGACGTTCATGCCCGAAAGCGTGGGTAGCAAACAGG
Neisseria 4	lactamica	24%	6%	10%	5%	2,2%	0,5%	1,1%	0,1%	TACGTAGGGTGCGAGCGTTAATCGGAATTACTGGGCGTAAAGCGGGCGCAGACGGTTACTTAAGCAGGATGTGAAATCCCCGGGCTCAACCCGGGAATTGCGTTCTGAACTGGGTGGCTAGAGTGTGTCAGAGGGAGGTAGAATTCCACGTGTAGCAGTGAAATGCGTAGAGATGTGGAGGAATACCGATGGCGAAGGCAGCCTCCTGGGATAACACTGACGTTCATGCCCGAAAGCGTGGGTAGCAAACAGG

Taxon Name	Species	Prevalence DNP (n=67)	Prevalence HNP_{All} (n=51)	Prevalence HNP_{CI} n=10	Prevalence HNP_{DC} (n=41)	Mean Relative Abundance DNP (n=67)	Mean Relative Abundance HNP_{All} (n=51)	Mean Relative Abundance HNP_{CI} (n=10)	Mean Relative Abundance HNP_{DC} (n=41)	Sequence
Neisseria 8 (Neisseria or Kingella)	N. elongata subsp. elongata/glycolytica/nitroreducens or K. denitrificans	10%	12%	10%	12%	0,4%	0,1%	0,1%	0,1%	TACGTAGGGTGCGAGCGTTAATCGGAATTACTGGGCGTAAAGCGAGCGCAGACGGTTACTTAAGCAGGATGTGAAAT<br>CCCCGGGCTCAACCTGGGAACTGCGTTCTGAACTGGGTAGCTAGAGTATGTCAGAGGGGGGTAGAATTCCACGTGTA<br>GCAGTGAAATGCGTAGAGATGTGGAGGAATACCGATGGCGAAGGCAGCCCCCTGGGATAATACTGACGTTCATGCTC<br>GAAAGCGTGGGTAGCAAACAGG
Oribacterium 1	sinus	22%	20%	30%	17%	0,1%	0,1%	0,1%	0,1%	TACGTAGGGGGCAAGCGTTATCCGGATTTACTGGGTGTAAAGGGAGCGTAGACGGAATGGCAAGTCTGAAGTGAAAT<br>ACCCGGGCTCAACCTGGGAACTGCTTTGGAAACTGTTGTTCTAGAGTGTTGGAGAGGTAAGTGGAATTCCTGGTGTAG<br>CGGTGAAATGCGTAGATATCAGGAAGAACACCGGAGGCGAAGGCGGCTTACTGGACAATAACTGACGTTGAGGCTC<br>GAAAGCGTGGGGATCAAACAGG
Oribacterium 2	parvum	4%	4%	10%	2%	0,1%	0,0%	0,0%	0,0%	TACGTAGGGGGCAAGCGTTATCCGGATTTACTGGGTGTAAAGGGAGCGTAGACGGAATGGCAAGTCTGAAGTGAAA<br>ACCCCGGGCTCAACCTGGGGACTGCTTTGGAAACTGTTGTTCTAGAGTGTTGGAGAGGTAAGTGGAATTCCTGGTGTA<br>GCGGTGAAATGCGTAGATATCAGGAAGAACACCGGAGGCGAAGGCGGCTTACTGGACAATAACTGACGTTGAGGCT<br>CGAAAGCGTGGGGATCAAACAGG
Parvimonas 2	micra	7%	4%	10%	2%	0,1%	0,7%	1,4%	0,0%	TACGTATGGGGCGAGCGTTGTCCGGAATTATTGGGCGTAAAGGGTACGTAGGCGGTTTTTTAAGTCAGGTGTGAAAG<br>CGTGAGGCTTAACCTCATTAAGCACTTGAAACTGGAAGACTTGAGTGAAGGAGAGGAAAGTGGAATTCCTAGTGTAG<br>CGGTGAAATGCGTAGATATTAGGAGGAATACCGGTGGCGAAGGCGACTTTCTGGACTTTTACTGACGCTCAGGTACG<br>AAAGCGTGGGGAGCAAACAGG
Parvimonas 3	NA	3%	8%	10%	7%	0,0%	0,0%	0,0%	0,0%	TACGTATGGGGCGAGCGTTGTCCGGAATTATTGGGCGTAAAGGGTACGTAGGCGGCCTTTTAAGTCAGGTGTGAAAG<br>CGTGAGGCTTAACCTCATTAAGCACTTGAAACTGGAAGGCTTGAGTGAAGGAGAGGAAAGTGGAATTCCTAGTGTAG<br>CGGTGAAATGCGTAGATATTAGGAGGAATACCGGTGGCGAAGGCGACTTTCTGGACTTTTACTGACGCTCAGGTACG<br>AAAGCGTGGGGAGCAAACAGG
Peptoniphilus 1	grossensis/vaginalis/harei	1%	8%	30%	2%	0,3%	0,3%	0,4%	0,0%	TACGTAGGGGGCTAGCGTTGTCCGGAATCACTGGGCGTAAAGGGTTCGCAGGCGGAAATGCAAGTCAGGTGTAAAA<br>GGCAGTAGCTTAACTACTGTAAGCATTTGAAACTGCATATCTTGAGAAGAGTAGAGGTAAGTGGAATTTTTAGTGTAG<br>CGGTGAAATGCGTAGATATTAAAAAGAATACCGGTGGCGAAGGCGACTTACTGGGCTCATTCTGACGCTGAGGAACG<br>AAAGCGTGGGTAGCAAACAGG
Peptostreptococcus 1	stomatis	18%	12%	20%	10%	0,1%	0,0%	0,1%	0,0%	TACGTAGGGGGCTAGCGTTATCCGGATTTACTGGGCGTAAAGGGTGCGTAGGTGGTCCTTCAAGTCGGTGGTTAAAG<br>GCTACGGCTCAACCGTAGTAAGCCGCCGAAACTGGAGGACTTGAGTGCAGGAGAGGAAAGTGGAATTCCCAGTGTA<br>GCGGTGAAATGCGTAGATATTGGGAGGAACACCAGTAGCGAAGGCGGCTTTCTGGACTGCAACTGACACTGAGGCA<br>CGAAAGCGTGGGTAGCAAACAGG
Porphyromonas 1	NA	60%	33%	60%	27%	1,7%	0,6%	1,4%	0,1%	TACGGAGGATGCGAGCGTTATCCGGATTTATTGGGTTTAAAGGGTGCGTAGGCGGCCTGTTAAGTCAGCGGTGAAAT<br>CTAGGAGCTTAACTCCTAAATTGCCATTGAAACTGGCGGGCTTGAGTGTAGATGAGGTAGGCGGAATGCGTGGTGTA<br>GCGGTGGAATGCATAGATATCACGCAGAACTCCAATTGCGAAGGCAGCTTACTAAGGTACAACTGACGCTGAAGCAC<br>GAAAGCGTGGGTATCAAACAGG
Porphyromonas 2	NA	49%	20%	30%	17%	0,5%	0,3%	0,9%	0,1%	TACGGAGGATGCGAGCGTTATCCGGATTTATTGGGTTTAAAGGGTGCGTAGGCGGTTTGTTAAGTCAGCGGTGAAATC<br>TAGAGGCTCAACCTCGAAACTGCCGTTGAAACTGGCGAACTTGAGTGTAGATGAGGTAGGCGGAATTCGTGGTGTAG<br>CGGTGAAATGCATAGATATCACGAGGAACTCCGATTGCGAAGGCAGCTTACTAAGGTACAACTGACGCTGAAGCACG<br>AAAGCGTGGGTATCAAACAGG
Porphyromonas 3	NA	27%	14%	40%	7%	0,3%	0,6%	0,4%	0,9%	TACGGAGGATGCGAGCGTTATCCGGATTTATTGGGTTTAAAGGGTGCGTAGGCGGTTTGTTAAGTCAGCGGTGAAATC<br>TAGAGGCTCAACCTCGAAACTGCCGTTGAAACTGGCGAACTTGAGTGTAGATGAGGTAGGCGGAATTCGTGGTGTAG<br>CGGTGAAATGCATAGATATCACGAGGAACTCCAATTGCGAAGGCAGCTTACTAAGGTACAACTGACGCTGAAGCACG<br>AAAGCGTGGGTATCAAACAGG
Porphyromonas 4	pasteri	6%	4%	10%	2%	0,4%	0,2%	0,4%	0,0%	TACGGAGGATGCGAGCGTTATCCGGATTTATTGGGTTTAAAGGGTGCGTAGGCGGCCTGTTAAGTCAGCGGTGAAAT<br>CTAGGAGCTTAACTCCTAAATTGCCATTGATACTGGCGGGCTTGAGTGTAGATGAGGTAGGCGGAATGCGTGGTGTA<br>GCGGTGGAATGCATAGATATCACGCAGAACTCCGATTGCGAAGGCAGCTTACTAAGGTACAACTGACGCTGAAGCAC<br>GAAAGCGTGGGTATCAAACAGG

Taxon Name	Species	Prevalence				Mean Relative Abundance				Sequence
		DNP (n=67)	HNP_{All} (n=51)	HNP_{CI} n=10	HNP_{DC} (n=41)	DNP (n=67)	HNP_{All} (n=51)	HNP_{CI} (n=10)	HNP_{DC} (n=41)	
Prevotella 1	melaninogenica	54%	41%	70%	34%	1,2%	0,8%	1,9%	0,2%	TACGGAAGGTCCAGGCGTTATCCGGATTTATTGGGTTTAAAGGGAGCGTAGGCTGGAGATTAAGTGTGTTGTGAAAT<br>GTAGACGCTCAACGTCTGAATTGCAGCGCATACTGGTTTCCTTGAGTACGCACAACGTTGGCGGAATTCGTCGTGTAG<br>CGGTGAAATGCTTAGATATGACGAAGAACTCCGATTGCGAAGGCAGCTGACGGGAGCGCAACTGACGCTTAAGCTCG<br>AAGGTGCGGGTATCAAACAGG
Prevotella 10	salivae	25%	12%	30%	7%	0,2%	0,2%	0,3%	0,1%	TACGGAAGGTCCAGGCGTTATCCGGATTTATTGGGTTTAAAGGGAGCGTAGGCCGTGGATTAAGCGTGTTGTGAAAT<br>GTAGACGCTCAACGTCTGAATTGCAGCGCGAACTGGTTCACTTGAGTATGCACAACGTAGGCGGAATTCGTCGTGTA<br>GCGGTGAAATGCTTAGATATGACGAAGAACTCCGATTGCGAAGGCAGCTTACGGGAGCACAACTGACGCTGAAGCTC<br>GAAGGTGCGGGTATCAAACAGG
Prevotella 11	NA	18%	20%	10%	22%	0,4%	0,3%	0,2%	0,4%	TACGGAAGGTCCAGGCGTTATCCGGATTTATTGGGTTTAAAGGGAGCGTAGGCCGGAGATTAAGTGTGTTGTGAAAT<br>GTAGACGCTCAACGTCTGACTTGCAGCGCATACTGGTTTCCTTGAGTACGCACAACGTTGGCGGAATTCGTCGTGTAG<br>CGGTGAAATGCTTAGATATGACGAAGAACTCCGATTGCGAAGGCAGCTGACGGGAGCGCAACTGACGCTTAAGCTCG<br>AAGGTGCGGGTATCGAACAGG
Prevotella 12	NA	10%	4%	10%	2%	0,3%	0,1%	0,1%	0,1%	TACGGAAGGTCCAGGCGTTATCCGGATTTATTGGGTTTAAAGGGAGTGTAGGCGGTTTGTTAAGCGTGTTGTGAAATT<br>TAGATGCTCAACATTTAACTTGCAGCGCGAACTGTCAGACTTGAGTACACGCAACGTATGCGGAATTCATGGTGTAGC<br>GGTGAAATGCTTAGATATCATGAAGAACTCCGATTGCGAAGGCAGCATACGGGAGTGTAACTGACGCTTAAGCTCGA<br>AGGTGCGGGTATCGAACAGG
Prevotella 13	melaninogenica	13%	16%	10%	17%	0,4%	0,4%	0,6%	0,3%	TACGGAAGGTCCAGGCGTTATCCGGATTTATTGGGTTTAAAGGGAGCGTAGGCTGGAGATTAAGTGTGTTGTGAAAT<br>GTAGACGCTCAACGTCTGAATTGCAGCGCATACTGGTTTCCTTGAGTACGCACAACGTTGGCGGAATTCGTCGTGTAG<br>CGGTGAAATGCTTAGATATGACGAAGAACTCCGATTGCGAAGGCAGCTGACGGGAGCGCAACTGACGCTTAAGCTCG<br>AAGGTGCGGGTATCGAACAGG
Prevotella 15	NA	13%	4%	10%	2%	1,0%	4,7%	9,2%	0,1%	TACGGAAGGTCCGGGCGTTATCCGGATTTATTGGGTTTAAAGGGAGCGTAGGCCGCCCCTTAAGCGTGTTGTGAAAT<br>GCGGGTGCTCAACATCCGACTTGCAGCGCGAACTGGGGGGCTTGAGTGCGCCGAAAGTAGGCGGAATTCGTGGTGT<br>AGCGGTGAAATGCTTAGATATCACGAAGAACTCCGATTGCGAAGGCAGCTTACTGTAGCGCAACTGACGCTGATGCT<br>CGAAAGCGTGGGTATCGAACAGG
Prevotella 19	NA	21%	12%	10%	12%	0,2%	0,2%	0,5%	0,1%	TACGGAAGGTCCAGGCGTTATCCGGATTTATTGGGTTTAAAGGGAGCGTAGGCCGTTTGATAAGCGTGCTGTGAAATT<br>TAGAGGCTCAACCTCTACCGTGCAGCGCGAACTGTCAGACTTGAGTGCGTAGTAGGTAGGCGGAATTCGTGGTGTAG<br>CGGTGAAATGCTTAGATATCACGAAGAACTCCGATTGCGAAGGCAGCTTACCGTATCGCAACTGACGCTGATGCTCG<br>AAGGTGCGGGTATCGAACAGG
Prevotella 2	histicola	25%	8%	20%	5%	0,7%	0,4%	0,5%	0,2%	TACGGAAGGTCCGGGCGTTATCCGGATTTATTGGGTTTAAAGGGAGCGTAGGCTGGAGATTAAGTGTGTTGTGAAAT<br>GTAGACGCTCAACGTCTGACTTGCAGCGCATACTGGTTTCCTTGAGTACGCACAACGTTGGCGGAATTCGTCGTGTAG<br>CGGTGAAATGCTTAGATATGACGAAGAACTCCGATTGCGAAGGCAGCTGACGGGAGCGCAACTGACGCTGAAGCTC<br>GAAGGTGCGGGTATCGAACAGG
Prevotella 21	NA	19%	10%	20%	7%	0,1%	0,0%	0,1%	0,0%	TACGGAAGGTCCTGGTGTTATCCGGATTTATTGGGTTTAAAGGGAGCGTAGGCCGTAGATTAAGTGTGTTGTGAAATG<br>TAGGCGCCCAACGTCTGCCTTGCAGCGCAAACTGGTTTACTTGAGTACGCGCAACGCAGGCGGAATTCGTCGTGTAG<br>CGGTGAAATGCTTAGATATGACGAAGAACTCCTATTGCGAAGGCAGCTTGCGGGAGCGTTACTGACGCTGAAGCTCG<br>AAAGTGCGGGTATCGAACAGG
Prevotella 27	shahii	15%	6%	20%	2%	0,1%	0,0%	0,1%	0,0%	TACGGAAGGTCCAGGCGTTATCCGGATTTATTGGGTTTAAAGGGAGCGTAGGCCGCCTCTTAAGCGTGTTGTGAAATG<br>CGGGTGCTCAACATCCGACTTGCAGCGCGAACTGGGGGGCTTGAGTGCGCCGAAAGTAGGCGGAATTCGTGGTGTA<br>GCGGTGAAATGCTTAGATATCACGAAGAACTCCGATTGCGAAGGCAGCTTACTGTAGCGCAACTGACGCTGATGCTC<br>GAAAGCGTGGGTATCGAACAGG
Prevotella 3	nigrescens	24%	4%	10%	2%	1,9%	4,6%	8,9%	0,3%	TACGGAAGGTCCGGGCGTTATCCGGATTTATTGGGTTTAAAGGGAGTGTAGGCGGTCTGTTAAGCGTGTTGTGAAATT<br>TAGGTGCTCAACATTTAACTTGCAGCGCGAACTGTCAGACTTGAGTACACGCAGCGCAGGCGGAATTCATGGTGTAG<br>CGGTGAAATGCTTAGATATCATGAGGAACTCCGATCGCGAAGGCAGCCTGCGGGAGTGTTACTGACGCTTAAGCTCG<br>AAGGTGCGGGTATCGAACAGG

		Prevalence				Mean Relative Abundance				
**Taxon Name**	**Species**	**DNP (n=67)**	**HNP_{All} (n=51)**	**HNP_{CI} n=10**	**HNP_{DC} (n=41)**	**DNP (n=67)**	**HNP_{All} (n=51)**	**HNP_{CI} (n=10)**	**HNP_{DC} (n=41)**	**Sequence**
Prevotella 4	nanceiensis	36%	25%	30%	24%	0,5%	0,4%	1,3%	0,1%	TACGGAAGGTCCAGGCGTTATCCGGATTTATTGGGTTTAAAGGGTGCGTAGGCCGTTTGATAAGCGTGCTGTGAAATA<br>TAGTGGCTCAACCTCTATCGTGCAGCGCGAACTGTTGAACTTGAGTGCGTAGTAGGTAGGCGGAATTCGTGGTGTAGC<br>GGTGAAATGCTTAGATATCACGAAGAACTCCGATTGCGAAGGCAGCTTACCGTAACGTTACTGACGCTTAAGCACGA<br>AGGTGCGGGTATCGAACAGG
Prevotella 42	veroralis	4%	4%	10%	2%	0,2%	0,9%	1,8%	0,0%	TACGGAAGGTTCGGGCGTTATCCGGATTTATTGGGTTTAAAGGGAGCGTAGGCCGGAGATTAAGTGTGTTGTGAAAT<br>GTAGACGCTCAACGTCTGACTTGCAGCGCATACTGGTTTCCTTGAGTACGCACAACGTTGGCGGAATTCGTCGTGTAG<br>CGGTGAAATGCTTAGATATGACGAAGAACTCCGATTGCGAAGGCAGCTGACGGGAGCGCCACTGACGCTTAAGCTCG<br>AAGGTGCGGGTATCGAACAGG
Prevotella 45	NA	3%	8%	20%	5%	0,1%	0,2%	0,2%	0,2%	TACGGAAGGTCCGGGCGTTATCCGGATTTATTGGGTTTAAAGGGTGCGTAGGCCGTTTGATAAGCGTGCTGTGAAATA<br>TAGTGGCTCAACCTCTATCGTGCAGCGCGAACTGTCGAACTTGAGTGCGTAGTAGGTAGGCGGAATTCGTGGTGTAG<br>CGGTGAAATGCTTAGATATCACGAAGAACTCCGATTGCGAAGGCAGCTTACCGTAACGTTACTGACGCTTAAGCACGA<br>AGGTGCGGGTATCGAACAGG
Prevotella 49	NA	1%	4%	10%	2%	0,0%	0,1%	0,0%	0,1%	TACGGAAGGTTCGGGCGTTATCCGGATTTATTGGGTTTAAAGGGAGCGTAGGCCGTAGATTAAGCGTGTTGTGAAATG<br>TAGATGCTCAACATCTGACTTGCAGCGCGAACTGGTTTACTTGAGTGTGCGCAACGTAGGCGGAATTCGTCGTGTAGC<br>GGTGAAATGCTTAGATATGACGAAGAACTCCGATTGCGAAGGCAGCTTACGGGAGCACAACTGACGCTGAAGCTCGA<br>AGGTGCGGGTATCAAACAGG
Prevotella 5	NA	40%	18%	20%	17%	0,5%	0,1%	0,1%	0,1%	TACGGAAGGTCCAGGCGTTATCCGGATTTATTGGGTTTAAAGGGTGCGTAGGCCGTTTGATAAGCGTGCTGTGAAATA<br>TAGTGGCTCAACCTCTATCGTGCAGCGCGAACTGTCGAACTTGAGTGCGTAGTAGGTAGGCGGAATTCGTGGTGTAG<br>CGGTGAAATGCTTAGATATCACGAAGAACTCCGATTGCGAAGGCAGCTTACCGTAACGTTACTGACGCTTAAGCACGA<br>AGGTGCGGGTATCGAACAGG
Prevotella 54	NA	3%	4%	10%	2%	0,1%	0,1%	0,1%	0,2%	TACGGAAGGTCCGGGCGTTATCCGGATTTATTGGGTTTAAAGGGAGCGTAGGCCGTGGATTAAGCGTGTTGTGAAAT<br>GCAGGTGCTCAACGTCTGCACTGCAGCGCGAACTGGTTCACTTGAGTGTGCGCAACGCAGGCGGAATTCGTCGTGTA<br>GCGGTGAAATGCTTAGATATGACGAAGAACTCCGATTGCGAAGGCAGCTTGCGGGAGCACAACTGACGCTGAAGCTC<br>GAAAGTGCGGGTATCGAACAGG
Prevotella 74	NA	1%	4%	10%	2%	0,0%	0,0%	0,1%	0,0%	TACGGAAGGTCCAGGCGTTATCCGGATTTATTGGGTTTAAAGGGAGTGTAGGCGGTTTGTTAAGCGTGTTGTGAAATT<br>TAGGTGCTCAACATTTAACTTGCAGCGCGAACTGGCGAACTTGAGTGCACACAACGTATGCGGAATTCATGGTGTAGC<br>GGTGAAATGCTTAGATATCATGAAGAACTCCGATTGCGAAGGCAGCATACGGGAGTGTAACTGACGCTTAAGCTCGA<br>AGGTGCGGGTATCGAACAGG
Prevotella 8	pallens	28%	12%	30%	7%	0,3%	0,2%	0,4%	0,0%	TACGGAAGGTCCAGGCGTTATCCGGATTTATTGGGTTTAAAGGGAGTGTAGGCGGTTTGTTAAGCGTGTTGTGAAATT<br>TAGGTGCTCAACATTTAACTTGCAGCGCGAACTGTCAGACTTGAGTACACGCAACGTATGCGGAATTCATGGTGTAGC<br>GGTGAAATGCTTAGATATCATGAAGAACTCCGATTGCGAAGGCAGCATACGGGAGTGTAACTGACGCTTAAGCTCGA<br>AGGTGCGGGTATCGAACAGG
Prevotella 9	NA	16%	12%	30%	7%	0,4%	0,2%	0,3%	0,1%	TACGGAAGGTTCGGGCGTTATCCGGATTTATTGGGTTTAAAGGGAGCGTAGGCCGGAGATTAAGTGTGTTGTGAAAT<br>GTAGACGCTCAACGTCTGACTTGCAGCGCATACTGGTTTCCTTGAGTACGCACAACGTTGGCGGAATTCGTCGTGTAG<br>CGGTGAAATGCTTAGATATGACGAAGAACTCCGATTGCGAAGGCAGCTGACGGGAGCGCAACTGACGCTGAAGCTC<br>GAAGGTGCGGGTATCGAACAGG
Pseudoxanthomonas 4	kaohsiungensis/koreensis	1%	4%	10%	2%	0,0%	0,0%	0,0%	0,0%	TACGAAGGGTGCAAGCGTTACTCGGAATTACTGGGCGTAAAGCGTGCGTAGGTGGTTGTTTAAGTCTGTCGTGAAAG<br>CCCTGGGCTCAACCTGGGAATTGCGATGGATACTGGGCAACTAGAGTGTGGTAGAGGGTGGCGGAATTCCCGGTGTA<br>GCAGTGAAATGCGTAGAGATCGGGAGGAACATCCGTGGCGAAGGCGGCCACCTGGGCCAACACTGACACTGAGGC<br>ACGAAAGCGTGGGGAGCAAACAGG
Romboutsia 1	timonensis	1%	14%	10%	15%	0,0%	0,1%	0,0%	0,1%	TACGTAGGGGGCTAGCGTTATCCGGAATTACTGGGCGTAAAGGGTGCGTAGGTGGTTTCTTAAGTCAGAGGTGAAAG<br>GCTACGGCTCAACCGTAGTAAGCCTTTGAAACTGGGAAACTTGAGTGCAGGAGAGGAGAGTGGAATTCCTAGTGTAG<br>CGGTGAAATGCGTAGATATTAGGAGGAACACCAGTTGCGAAGGCGGCTCTCTGGACTGTAACTGACACTGAGGCACG<br>AAAGCGTGGGGAGCAAACAGG

Taxon Name	Species	Prevalence DNP (n=67)	Prevalence HNP_{All} (n=51)	Prevalence HNP_{CI} n=10	Prevalence HNP_{DC} (n=41)	Mean Relative Abundance DNP (n=67)	Mean Relative Abundance HNP_{All} (n=51)	Mean Relative Abundance HNP_{CI} (n=10)	Mean Relative Abundance HNP_{DC} (n=41)	Sequence
Rothia 1	mucilaginosa	16%	16%	30%	12%	0,1%	0,4%	0,4%	0,4%	TACGTAGGGCGCGAGCGTTGTCCGGAATTATTGGGCGTAAAGAGCTTGTAGGCGGTTTGTCGCGTCTGCTGTGAAAGGCCGGGGCTTAACCCCGTGTATTGCAGTGGGTACGGGCAGACTAGAGTGCAGTAGGGGAGACTGGAATTCCTGGTGTAGCGGTGGAATGCGCAGATATCAGGAGGAACACCGATGGCGAAGGCAGGTCTCTGGGCTGTAACTGACGCTGAGAAGCGAAAGCATGGGGAGCGAACAGG
Rothia 2	NA	31%	14%	30%	10%	0,3%	0,3%	0,5%	0,1%	TACGTAGGGCGCGAGCGTTGTCCGGAATTATTGGGCGTAAAGAGCTTGTAGGCGGTTTGTCGCGTCTGCTGTGAAAGGCCGGAGCTTAACTCCGTGTATTGCAGTGGGTACGGGCAGACTAGAGTGCAGTAGGGGAGACTGGAATTCCTGGTGTAGCGGTGGAATGCGCAGATATCAGGAGGAACACCGATGGCGAAGGCAGGTCTCTGGGCTGTAACTGACGCTGAGAAGCGAAAGCATGGGGAGCGAACAGG
Rothia 4	mucilaginosa	10%	16%	10%	17%	0,1%	0,3%	0,1%	0,3%	TACGTAGGGCGCGAGCGTTGTCCGGAATTATTGGGCGTAAAGAGCTTGTAGGCGGTTTGTCGCGTCTGCTGTGAAAGGCCGGGGCTTAACTCCGTGTATTGCAGTGGGTACGGGCAGACTAGAGTGCAGTAGGGGAGACTGGAATTCCTGGTGTAGCGGTGGAATGCGCAGATATCAGGAGGAACACCGATGGCGAAGGCAGGTCTCTGGGCTGTAACTGACGCTGAGAAGCGAAAGCATGGGGAGCGAACAGG
Rothia 7	mucilaginosa	1%	6%	20%	2%	0,2%	0,3%	0,3%	0,4%	TACGTAGGGCGCGAGCGTTGTCCGGAATTATTGGGCGTAAAGAGCTTGTAGGCGGTTTGTCGCGTCTGCTGTGAAAGGCCGGGGCTTAACCCCGTGTATTGCAGTGGGTACGGGCAGACTAGAGTGCAGTAGGGGAGACTGGAACTCCTGGTGTAGCGGTGGAATGCGCAGATATCAGGAAGAACACCGATGGCGAAGGCAGGTCTCTGGGCTGTAACTGACGCTGAGAAGCGAAAGCATGGGGAGCGAACAGG
Selenomonas 1	NA	21%	14%	40%	7%	0,1%	0,0%	0,1%	0,0%	TACGTAGGTGGCGAGCGTTGTCCGGAATCATTGGGCGTAAAGGGAGCGCAGGCGGGCCGGTAAGTCTTACTTAAAAGTGCGGGGCTCAACCCCGTGATGGGAGAGAAACTATCGGTCTTGAGTACAGGAGAGGAAAGCGGAATTCCCAGTGTAGCGGTGAAATGCGTAGATATTGGGAAGAACACCAGTGGCGAAGGCGGCTTTCTGGACTGCAACTGACGCTGAGGCTCGAAAGCCAGGGGAGCGAACGGG
Selenomonas 2	sputigena	15%	8%	20%	5%	0,1%	0,0%	0,0%	0,0%	TACGTAGGTGGCGAGCGTTGTCCGGAATTATTGGGCGTAAAGGGAGCGCAGGCGGACATATAAGTCCATCTTAAAAGTGCGGGGCTCAACCCCGTGAGGGGATGGAAACTGTATGCCTTGAGTGCAGGAGAGGAAAGCGGAATTCCCAGTGTAGCGGTGAAATGCGTAGATATTGGGAGGAACACCAGTGGCGAAGGCGGCTTTCTGGACTGTAACTGACGCTGAGGCTCGAAAGCCAGGGGAGCGAACGGG
Selenomonas 3	NA	7%	4%	10%	2%	0,1%	0,0%	0,0%	0,0%	TACGTAGGTGGCGAGCGTTGTCCGGAATCATTGGGCGTAAAGGGAGCGCAGGCGGGCATGTAAGTCTTTCTTAAAAGTTCGGGGCTCAACCCCGTGATGGGAAAGAAACTACATGTCTTGAGTACAGGAGAGGAAAGCGGAATTCCCAGTGTAGCGGTGAAATGCGTAGATATTGGGAGGAACACCAGTGGCGAAGGCGGCTTTCTGGACTGCAACTGACGCTGAGGCTCGAAAGCCAGGGGAGCGAACGGG
Staphylococcus 1	aureus subsp. aureus/anaerobius | argenteus/caprae/epidermidis/haemolyticus/lugdunensis/pasteuri/saccharolyticus/schweitzeri/simiae/warneri | petrasii subsp. croceilyticus/petrasii/pragensis/jettensis | capitis subsp. capitis | hominis subsp. novobiosepticus[illegible]	42%	61%	50%	63%	2,7%	0,8%	0,4%	0,9%	TACGTAGGTGGCAAGCGTTATCCGGAATTATTGGGCGTAAAGCGCGCGTAGGCGGTTTTTTAAGTCTGATGTGAAAGCCCACGGCTCAACCGTGGAGGGTCATTGGAAACTGGAAAACTTGAGTGCAGAAGAGGAAAGTGGAATTCCATGTGTAGCGGTGAAATGCGCAGAGATATGGAGGAACACCAGTGGCGAAGGCGACTTTCTGGTCTGTAACTGACGCTGATGTGCGAAAGCGTGGGGATCAAACAGG
Streptobacillus 1 (Pseudostreptobacillus)	hongkongensis	36%	12%	30%	7%	0,4%	0,1%	0,1%	0,0%	TACGTATGTCGCAAGCGTTATCCGGAATTATTGGGCTTAAAGGGCATCTAGGCGGTTAAACAAGTCAGGGGTGAAAACTTGCAGCTCAACTGCAAGCTTGCCTTTGAAACTGATTAACTAGAGTACTGGAAAGGTGGGTGGAACTACACGAGTAGAGGTGAAATTCGTAGATATGTGTAGGAATGCCGATGATGAAGATAACTCACTGGACAGAAACTGACGCTGAAGTGCGAAAGCTAGGGGAGCAAACAGG
Streptobacillus 2	NA	25%	8%	20%	5%	0,3%	1,0%	2,0%	0,1%	TACGTATGTCGCAAGCGTTATCCGGAATTATTGGGCTTAAAGGGCATCTAGGCGGTATTTCAAGTCAGGGGTGAAAACTTATGGCTCAACCATAAGCTTGCCTTTGAAACTGAAAGACTAGAGTACTGGAAAGGTGGGTGGAACTACACGAGTAGAGGTGAAATTCGTAGATATGTGTAGGAATGCCGATGATGAAGATAACTCACTGGACAGAAACTGACGCTGAAGTGCGAAAGCTAGGGGAGCAAACAGG

Taxon Name	Species	Prevalence				Mean Relative Abundance				Sequence
		DNP (n=67)	HNP_{All} (n=51)	HNP_{CI} n=10	HNP_{DC} (n=41)	DNP (n=67)	HNP_{All} (n=51)	HNP_{CI} (n=10)	HNP_{DC} (n=41)	
Streptobacillus 3	NA	30%	16%	30%	12%	0,2%	0,2%	0,3%	0,1%	TACGTATGTCGCAAGCGTTATCCGGAATTATTGGGCTTAAAGGGCATCTAGGCGGTTTTTCAAGTCAGGGGTGAAAAC<br>TTATGGCTCAACTATAAGCTTGCCTTTGAAACTGAAAGACTAGAGTACTGGAAAGGTGGGTGGAACTACACGAGTAGA<br>GGTGAAATTCGTAGATATGTGTAGGAATGCCGATGATGAAGATAACTCACTGGACAGAAACTGACGCTGAAGTGCGA<br>AAGCTAGGGGAGCAAACAGG
Streptococcus 1	pneumoniae/pseudopneumoniae	66%	55%	60%	54%	8,0%	9,5%	6,7%	10,3%	TACGTAGGTCCCGAGCGTTGTCCGGATTTATTGGGCGTAAAGCGAGCGCAGGCGGTTAGATAAGTCTGAAGTTAAAG<br>GCTGTGGCTTAACCATAGTAGGCTTTGGAAACTGTTTAACTTGAGTGCAAGAGGGGAGAGTGGAATTCCATGTGTAGC<br>GGTGAAATGCGTAGATATATGGAGGAACACCGGTGGCGAAAGCGGCTCTCTGGCTTGTAACTGACGCTGAGGCTCGA<br>AAGCGTGGGGAGCAAACAGG
Streptococcus 11	intermedius	9%	6%	10%	5%	0,2%	0,9%	2,7%	0,0%	TACGTAGGTCCCGAGCGTTGTCCGGATTTATTGGGCGTAAAGCGAGCGCAGGCGGTTAGATAAGTCTGAAGTTAAAG<br>GCAGTGGCTCAACCATTGTAGGCTTTGGAAACTGTTTAACTTGAGTGCAGAAGGGGAGAGTGGAATTCCATGTGTAGC<br>GGTGAAATGCGTAGATATATGGAGGAACACCGGTGGCGAAAGCGGCTCTCTGGTCTGTAACTGACGCTGAGGCTCGA<br>AAGCGTGGGGAGCGAACAGG
Streptococcus 19	anginosus subsp. anginosus/whileyi	1%	4%	10%	2%	0,1%	0,3%	0,2%	0,4%	TACGTAGGTCCCGAGCGTTGTCCGGATTTATTGGGCGTAAAGCGAGCGCAGGCGGTTAGAAAAGTCTGAAGTGAAAG<br>GCAGTGGCTCAACCATTGTAGGCTTTGGAAACTGTTTAACTTGAGTGCAGAAGGGGAGAGTGGAATTCCATGTGTAGC<br>GGTGAAATGCGTAGATATATGGAGGAACACCGGTGGCGAAAGCGGCTCTCTGGTCTGTAACTGACGCTGAGGCTCGA<br>AAGCGTGGGGAGCGAACAGG
Streptococcus 2	mitis/infantis | oralis subsp. oralis/tigurinus/dentisani	70%	69%	80%	66%	1,9%	2,9%	2,7%	2,9%	TACGTAGGTCCCGAGCGTTGTCCGGATTTATTGGGCGTAAAGCGAGCGCAGGCGGTTAGATAAGTCTGAAGTTAAAG<br>GCTGTGGCTTAACCATAGTACGCTTTGGAAACTGTTTAACTTGAGTGCAAGAGGGGAGAGTGGAATTCCATGTGTAGC<br>GGTGAAATGCGTAGATATATGGAGGAACACCGGTGGCGAAAGCGGCTCTCTGGCTTGTAACTGACGCTGAGGCTCGA<br>AAGCGTGGGGAGCAAACAGG
Streptococcus 4	NA	4%	16%	20%	15%	12,9%	8,9%	4,0%	10,6%	TACGTAGGTCCCGAGCGTTGTCCGGATTTATTGGGCGTAAAGCGAGCGCAGGCGGTTAGATAAGTCTGAAGTTAAAG<br>GCTGTGGCTTAACCATAGTAGGCTTTGGAAACTGTTTAACTTGAGTGCAAGAGGGGAGAGTGGAATTCCATGTGTAGC<br>GGTGAAATGCGTAGATATATGGAGGAACACCAGTGGCGAAAGCGGCTCTCTGGCTTGTAACTGACGCTGAGGCTCGA<br>AAGCGTGGGGAGCAAACAGG
Streptococcus 5	salivarius/thermophilus/vestibularis	45%	73%	60%	76%	0,2%	0,4%	0,2%	0,4%	TACGTAGGTCCCGAGCGTTGTCCGGATTTATTGGGCGTAAAGCGAGCGCAGGCGGTTTGATAAGTCTGAAGTTAAAG<br>GCTGTGGCTCAACCATAGTTCGCTTTGGAAACTGTCAAACTTGAGTGCAGAAGGGGAGAGTGGAATTCCATGTGTAG<br>CGGTGAAATGCGTAGATATATGGAGGAACACCGGTGGCGAAAGCGGCTCTCTGGTCTGTAACTGACGCTGAGGCTCG<br>AAAGCGTGGGGAGCGAACAGG
Streptococcus 7	parasanguinis/australis/rubneri	24%	24%	30%	22%	0,2%	0,5%	0,3%	0,5%	TACGTAGGTCCCGAGCGTTATCCGGATTTATTGGGCGTAAAGCGAGCGCAGGCGGTTAGATAAGTCTGAAGTTAAAG<br>GCTGTGGCTTAACCATAGTACGCTTTGGAAACTGTTTAACTTGAGTGCAGAAGGGGAGAGTGGAATTCCATGTGTAGC<br>GGTGAAATGCGTAGATATATGGAGGAACACCGGTGGCGAAAGCGGCTCTCTGGTCTGTAACTGACGCTGAGGCTCGA<br>AAGCGTGGGGAGCAAACAGG
Streptococcus 8	sanguinis	9%	16%	20%	15%	0,2%	0,1%	0,3%	0,1%	TACGTAGGTCCCGAGCGTTGTCCGGATTTATTGGGCGTAAAGCGAGCGCAGGCGGTTAGATAAGTCTGAAGTTAAAG<br>GCTGTGGCTTAACCATAGTATGCTTTGGAAACTGTTTAACTTGAGTGCAGAAGGGGAGAGTGGAATTCCATGTGTAGC<br>GGTGAAATGCGTAGATATATGGAGGAACACCGGTGGCGAAAGCGGCTCTCTGGTCTGTAACTGACGCTGAGGCTCGA<br>AAGCGTGGGGAGCAAACAGG
Veillonella 1	dispar/infantium	60%	37%	60%	32%	1,0%	0,6%	1,3%	0,3%	TACGTAGGTGGCAAGCGTTGTCCGGAATTATTGGGCGTAAAGCGCGCGCAGGCGGATTGGTCAGTCTGTCTTAAAAG<br>TTCGGGGCTTAACCCCGTGATGGGATGGAAACTGCCAATCTAGAGTATCGGAGAGGAAAGTGGAATTCCTAGTGTAG<br>CGGTGAAATGCGTAGATATTAGGAAGAACACCAGTGGCGAAGGCGACTTTCTGGACGAAAACTGACGCTGAGGCGC<br>GAAAGCCAGGGGAGCGAACGGG
Veillonella 2	NA	72%	55%	60%	54%	0,9%	0,7%	1,1%	0,6%	TACGTAGGTGGCAAGCGTTGTCCGGAATTATTGGGCGTAAAGCGCGCGCAGGCGGCCTATCCAGTCTGTCTTAAAAG<br>TTCGGGGCTCAACCCCGTGATGGGATGGAAACTAGTAGGCTAGAGTATCGGAGAGGAAAGCGGAATTCCTAGTGTAG<br>CGGTGAAATGCGTAGATATTAGGAAGAACACCAGTGGCGAAGGCGGCTTTCTGGACGAAAACTGACGCTGAGGCGC<br>GAAAGCCAGGGGAGCGAACGGG

Taxon Name	Species	Prevalence				Mean Relative Abundance				Sequence
		DNP (n=67)	HNP_{All} (n=51)	HNP_{CI} n=10	HNP_{DC} (n=41)	DNP (n=67)	HNP_{All} (n=51)	HNP_{CI} (n=10)	HNP_{DC} (n=41)	
Veillonella 3	parvula/tobetsuensis	42%	43%	40%	44%	0,8%	0,7%	0,6%	0,7%	TACGTAGGTGGCAAGCGTTGTCCGGAATTATTGGGCGTAAAGCGCGCGCAGGCGGATCAGTCAGTCTGTCTTAAAAGTTCGGGGCTTAACCCCGTGATGGGATGGAAACTGCTGATCTAGAGTATCGGAGAGGAAAGTGGAATTCCTAGTGTAGCGGTGAAATGCGTAGATATTAGGAAGAACACCAGTGGCGAAGGCGACTTTCTGGACGAAAACTGACGCTGAGGCGCGAAAGCCAGGGGAGCGAACGGG
Veillonella 4	NA	10%	14%	10%	15%	0,6%	0,7%	0,1%	0,8%	TACGTAGGTGGCAAGCGTTGTCCGGAATTATTGGGCGTAAAGCGCGCGCAGGCGGATAGGTCAGTCTGTCTTAAAAGTTCGGGGCTTAACCCCGTGATGGGATGGAAACTGCCAATCTAGAGTATCGGAGAGGAAAGTGGAATTCCTAGTGTAGCGGTGAAATGCGTAGATATTAGGAAGAACACCAGTGGCGAAGGCGACTTTCTGGACGAAAACTGACGCTGAGGCGCGAAAGCCAGGGGAGCGAACGGG
Veillonella 5	atypica/rogosae	12%	12%	20%	10%	0,5%	0,5%	0,6%	0,5%	TACGTAGGTGGCAAGCGTTGTCCGGAATTATTGGGCGTAAAGCGCGCGCAGGCGGATCAGTTAGTCTGTCTTAAAAGTTCGGGGCTTAACCCCGTGATGGGATGGAAACTGCTGATCTAGAGTATCGGAGAGGAAAGTGGAATTCCTAGTGTAGCGGTGAAATGCGTAGATATTAGGAAGAACACCAGTGGCGAAGGCGACTTTCTGGACGAAAACTGACGCTGAGGCGCGAAAGCCAGGGGAGCGAACGGG
