# Supplementary figures and images for: Case-Control Microbiome Study of Chronic Otitis Media with Effusion in Children Points at Streptococcus salivarius as a Pathobiont-Inhibiting Species
Source: mSystems. 2021 Apr 20;6(2):e00056-21. doi: 10.1128/mSystems.00056-21 (PMC8546964; doi:10.1128/mSystems.00056-21)

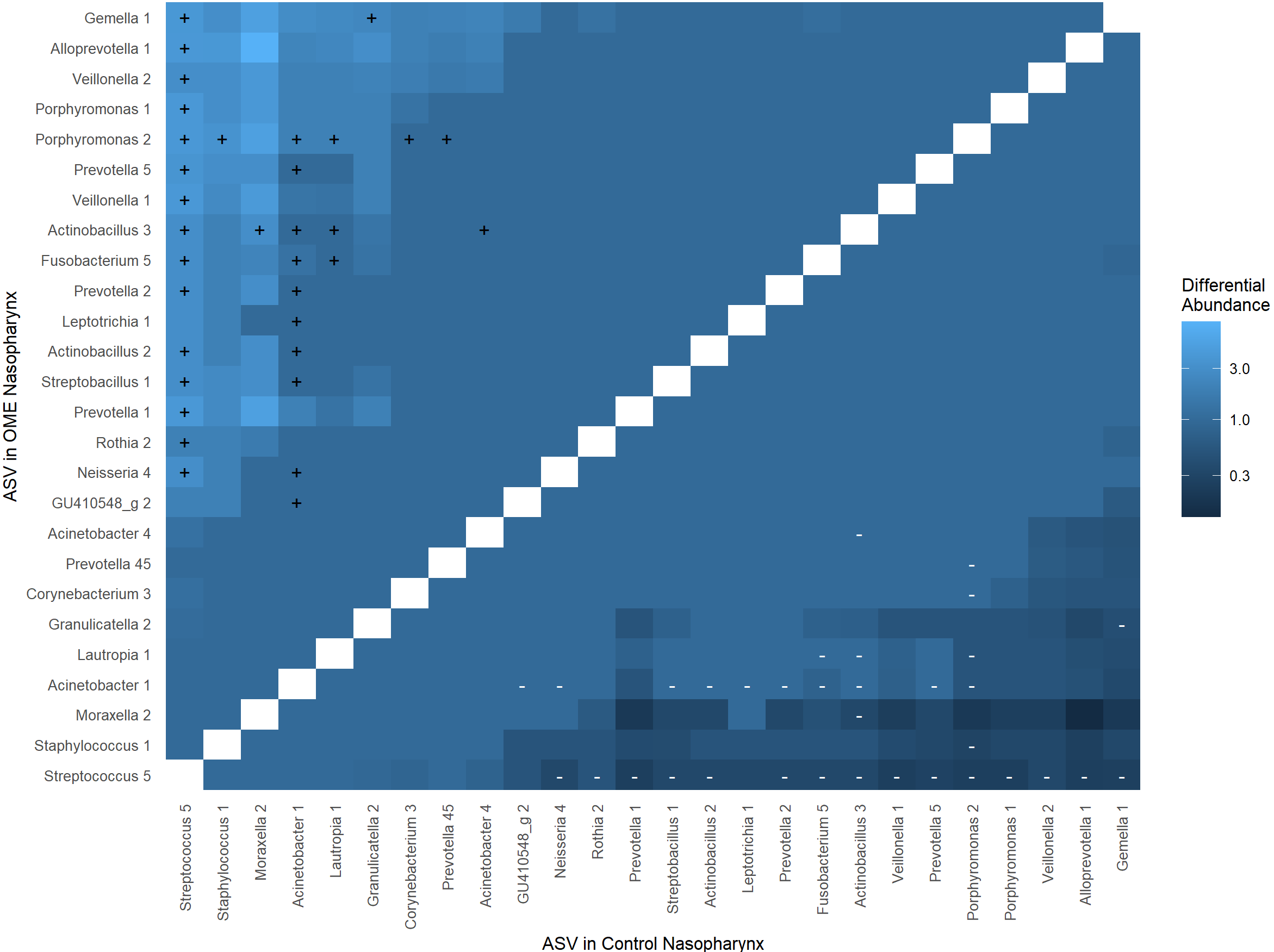

Supplement: FIG S1 [file msystems.00056-21-sf001.tif]

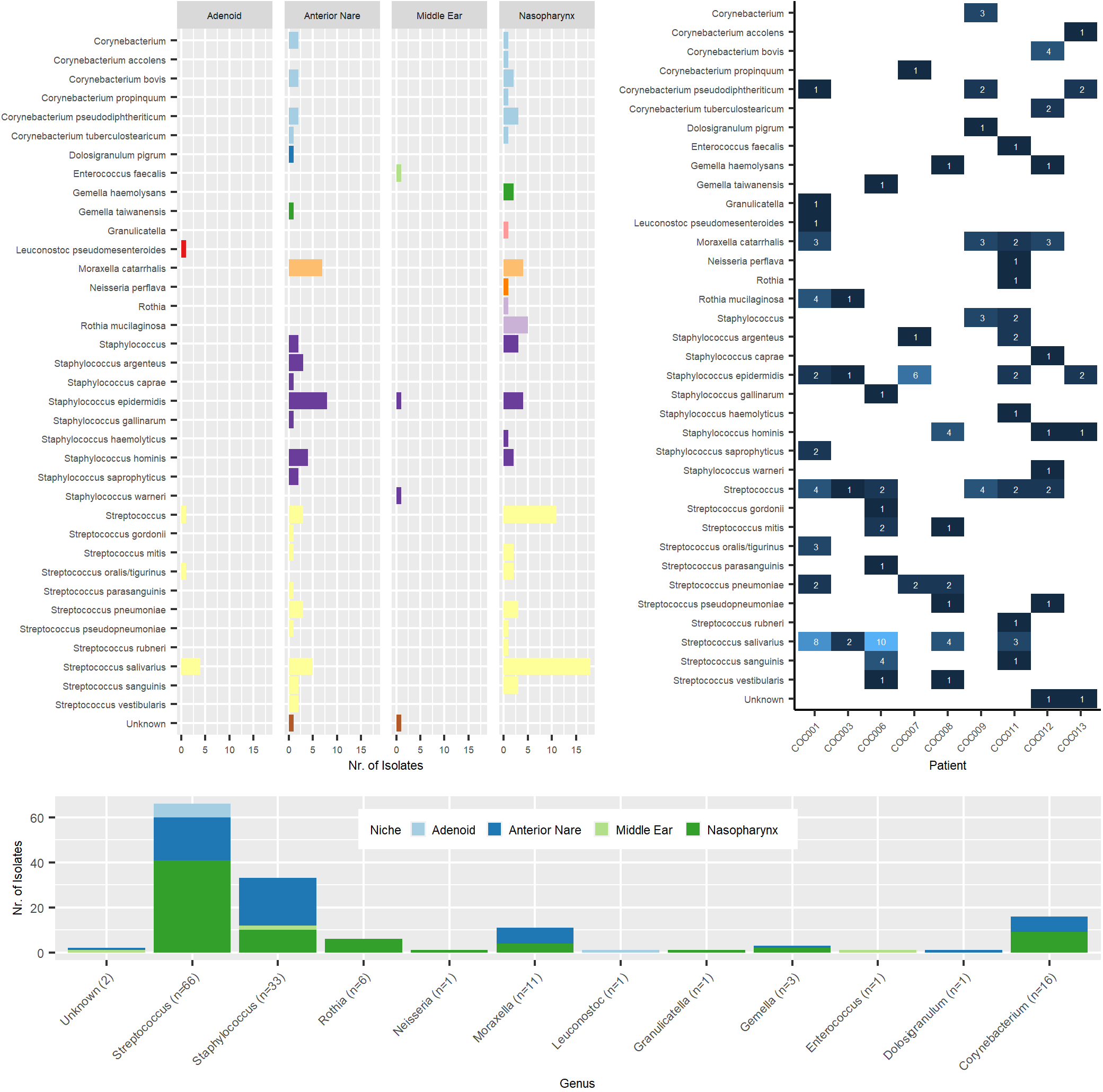

Supplement: FIG S2 [file msystems.00056-21-sf002.tif]

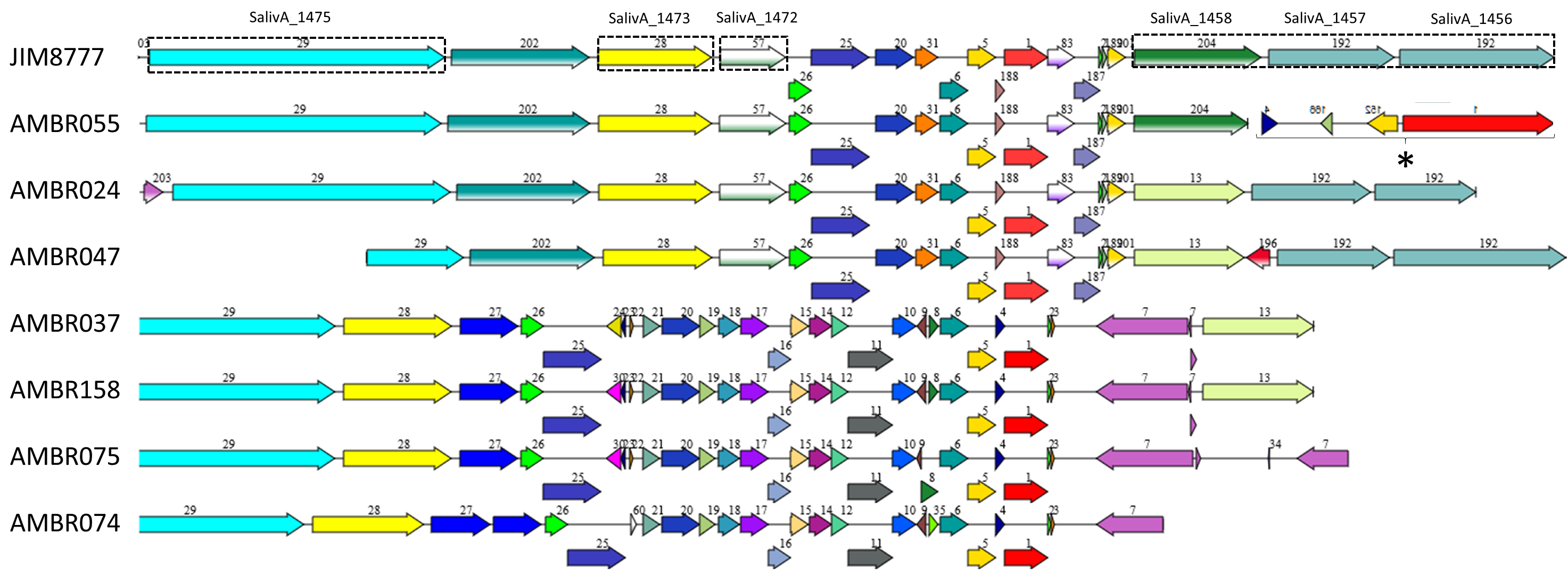

Supplement: FIG S3 [file msystems.00056-21-sf003.tif]
