# Supplementary material for: Case-Control Microbiome Study of Chronic Otitis Media with Effusion in Children Points at Streptococcus salivarius as a Pathobiont-Inhibiting Species
Source: mSystems. 2021 Apr 20;6(2):e00056-21. doi: 10.1128/mSystems.00056-21 (PMC8546964; doi:10.1128/mSystems.00056-21)
Supplement: TABLE S4 [file msystems.00056-21-st004.docx]

**Table S4:** Antibiotic resistance and virulence factors genes

| **Isolate** | **Gene** | **Product** | **%Identity^a^** | **%Coverage** |
| --- | --- | --- | --- | --- |
| **Antibiotic Resistance Genes (Resfinder)** | | | | |
| AMBR047 | *mel* | ABC-F type ribosomal protection protein Msr(D) | 100.00 | 100.00 |
|  | *mefA* |  | 99.92 | 100.00 |
| AMBR055 | *mel* | Macrolide resistance, MFS efflux pump Mef(A) | 99.93 | 100.00 |
|  | *mefA* |  | 100.00 | 100.00 |
| **Virulence Factor Genes (VFDB)** | | | | |
| AMBR024 | *hasC* | UDP-glucose pyrophosphorylase [Hyaluronic acid capsule] | 77.53 | 91.69 |
|  | *psaA* | Manganese ABC transporter manganese-binding adhesion liprotein | 76.24 | 88.92 |
| AMBR037 | *hasC* | UDP-glucose pyrophosphorylase [Hyaluronic acid capsule] | 77.32 | 91.58 |
|  | *psaA* | Manganese ABC transporter manganese-binding adhesion liprotein | 76.48 | 88.92 |
| AMBR047 | *hasC* | UDP-glucose pyrophosphorylase [Hyaluronic acid capsule] | 77.06 | 91.26 |
|  | *psaA* | Manganese ABC transporter manganese-binding adhesion liprotein | 76.96 | 88.92 |
| AMBR055 | *hasC* | UDP-glucose pyrophosphorylase [Hyaluronic acid capsule] | 77.41 | 91.69 |
|  | *psaA* | Manganese ABC transporter manganese-binding adhesion liprotein | 76.60 | 88.92 |
| AMBR074 | *hasC* | UDP-glucose pyrophosphorylase [Hyaluronic acid capsule] | 77.67 | 91.58 |
|  | *psaA* | Manganese ABC transporter manganese-binding adhesion liprotein | 76.68 | 87.85 |
| AMBR075 | *hasC* | UDP-glucose pyrophosphorylase [Hyaluronic acid capsule] | 77.32 | 91.58 |
|  | *psaA* | Manganese ABC transporter manganese-binding adhesion liprotein | 76.48 | 88.92 |
| AMBR158 | *hasC* | UDP-glucose pyrophosphorylase [Hyaluronic acid capsule] | 76.94 | 91.26 |
|  | *psaA* | Manganese ABC transporter manganese-binding adhesion liprotein | 76.60 | 88.92 |
| ^a^ Reference genes: hasC - *Streptococcus pyogenes* [WP_010922799.1], psaA - *Streptococcus pneumoniae* [WP_000733059], mel - *S. pneumoniae* GA3488 [AF274302], mefA - *S. pneumoniae* 02J1175 [U83667] | | | | |
