# Supplementary material for: Case-Control Microbiome Study of Chronic Otitis Media with Effusion in Children Points at Streptococcus salivarius as a Pathobiont-Inhibiting Species
Source: mSystems. 2021 Apr 20;6(2):e00056-21. doi: 10.1128/mSystems.00056-21 (PMC8546964; doi:10.1128/mSystems.00056-21)
Supplement: TEXT S1 [file msystems.00056-21-s0001.docx]

**Supplementary Material and Methods**

**Sample Collection and Processing**

***Ethics Statement***

Ethical approval was obtained from the ethical committee of the Antwerp University Hospital for inclusion of OME patients and cochlear implant recipients (B300201731724), for the NPcarriage study (B300201526558) and for *16S rRNA* gene sequencing of a subset of samples thereof (B300201940224).

***Sample Collection***

This case-control study compared the URT and ear microbiota of children with persistent OME to that of microbiologically healthy children without a history of otitis media. Samples of patients with OME and cochlear implant controls were taken under general anaesthesia at the time of the intervention (placement of ventilation tubes with/without adenoidectomy or cochlear implantation). From both groups the anterior nares and nasopharynx (transnasally) were sampled using dry Nylon FLOQSwabs (503CS01, COPAN). In OME patients, the ear canal was sampled with the same swab type after removal of cerumen. In case of concurrent transoral adenoidectomy, the adenoid tissue was collected and swabbed *ex situ* using larger dry Nylon FLOQSwabs (511CS01, COPAN). OME middle ear effusion was aspirated into 40 mL Argyle^TM^ Specimen Traps (Covidien) after myringotomy of the tympanic membrane, using disposable metal suction tips (Mediplast) and suction tip handles with suction control (Mediplast) from patient 14 on. The surgeons were asked to avoid touching the ear canal. To reach the middle ear cavity of the healthy controls for cochlear implant placement, the surgeon performed a mastoidectomy. As soon as the middle ear cavity was reached, it was washed with 0.9% NaCl which was subsequently aspirated into a 40 mL Argyle^TM^ Specimen Trap. As cochlear implant surgery involves placement of a foreign body (the implant), patients received perioperative intravenous antibiotics (Amoxicillin/Clavulanic acid or Clindamycin), but local antibiotic treatment was withheld until after sampling. Swab and tissue samples were stored in 850 µL PowerBead solution (the first buffer of the QIAamp PowerFecal DNA Kit, QIAGEN, Cat No. 12830-50). All samples were transported on ice and processed on the day of surgery. In the laboratory, the suction tips and the tubing of the Argyle^TM^ Specimen Trap used for OME patients were flushed repeatedly with the same volume of 850 µL PowerBead solution to dislodge trapped middle ear effusion. Middle ear washes of controls were pelleted in 50 mL centrifuge tubes (Greiner) and the pellet was resuspended in 850 µL PowerBead solution. Tubes containing swabs or adenoid tissue were vortexed vigorously. Of each sample, 750 µL were used for DNA extraction, while 100 µL were either plated out on microbiological media or combined with 100 µL of 50% glycerol and frozen at -80°C to preserve viability for potential future bacterial isolation (OME samples).

***DNA Extraction***

DNA was extracted with the QIAamp PowerFecal DNA Kit according to the protocol. The DNA concentration was quantified with the Qubit 3.0 Fluorometer (Thermo Fisher Scientific). The remaining DNA was stored at -20˚C until further processing.

***16S rRNA V4 Gene Amplification and Gel Electrophoresis***

PCR amplification of the V4 region of the bacterial *16S rRNA* gene was performed in a 96-well format and each plate included two negative controls. All samples were amplified using the barcoded primers 515F (5′‐TATGGTAATTGTGTGCCAGCMGCCGCGGTAA‐3′) and 806R (5′‐AGTCAGTCAGCCGGACTACHVGGGTWTCTAAT‐3′) whereby each sample within a run was indexed with a unique combination of a forward and a reverse primer-barcode [1].

The PCR mix consisted of 200 µM deoxyribose nucleoside triphosphates (dNTPs), 3% dimethyl sulfoxide (DMSO), 1x Phusion HF Buffer, 0.4 units of Phusion^TM^ High-Fidelity DNA Polymerase (Thermo Fisher Scientific), 0.5 µM each of forward and reverse primer and a maximum of 50 ng or 5 µL of DNA extract. This mixture was supplemented with PCR-grade water to a final volume of 20 µL.

The 96-well-plate was sealed, centrifuged for a few seconds and amplified with the following PCR conditions: 30 cycles of 20 sec denaturation at 95°C, followed by 20 sec annealing at 55°C and 1 min elongation at 72°C. Cycling was preceded by an initial denaturation step of 2 min and concluded with a final elongation step of 10 min.

To confirm successful amplification, 1 µL of each amplicon was combined with 5 µL 1.2x loading dye, run for 30 min at 100 V in a 1.2% agarose gel with GelRed^®^ Nucleic Acid Gel Stain (Biotium) and subsequently visualized under UV light. The GeneRuler 1 kb Plus DNA Ladder (Thermo Fisher Scientific) was included to identify bands with the expected size of ~282 bp.

***PCR Clean-up, Pooling and Gel Extraction of Library***

Once all PCRs for a MiSeq run had been completed, the amplicons were purified using Agentcourt AMPure XP (Beckman Coulter) according to the protocol. This was followed by DNA quantification with the Qubit Fluorometer 3.0 and same-day equimolar pooling. One hundred eighty microlitres of the pooled library were combined with 36 µL 6x Loading Dye and loaded into 6 neighbouring wells of a 0.8% agarose gel with GelRed^®^ Nucleic Acid Gel Stain. The GeneRuler 1 kb Plus DNA Ladder (ThermoFisher Scientific) served as a size indicator. After running the gel for 50 minutes at 60 V, the DNA was briefly visualized under UV light and the gel section containing the library at ~282 bp was excised. The DNA was then extracted from the gel with the NucleoSpin^®^ Gel and PCR Clean-up kit (Macherey-Nagel) according to the protocol. After confirmation of the DNA concentration using the Qubit Fluorometer 3.0 the library was frozen.

***MiSeq Sequencing***

On the day of the MiSeq run, the library was quantified again, the concentration was converted from ng/µL to nM using the formula below, and a 20 µL library at a concentration of 2 nM was created, 5 µL of which were loaded on the MiSeq sequencing machine.

$$x\frac{ng}{\mu L} x {10}^{6}*\frac{1}{660}*\frac{1}{282}=nM$$

**Supplementary Samples**

Due to the low number of healthy participants in the clinical study, 41 nasopharynx swab samples of healthy Flemish children collected by the VAXINFECTIO research group (UAntwerp) in the NPcarriage Study [2,3] were included. The DNA of these samples had already been extracted using the NucliSENS^®^ easyMAG^®^ automatic extraction platform (BIOMÉRIEUX) followed by storage at -80°C. The DNA was further processed according to the pipeline for the OME Microbiome Study.

***Negative controls and sterility assurance***

For each 50-unit extraction kit and 96-well PCR plate, three extraction controls and two PCR controls were included, respectively, as negative controls, because of the low biomass nature of URT samples. Samples were prepared for DNA extraction in a UV- and ethanol-sterilized laminar flow hood, while the DNA was extracted in a dedicated room after sterilizing the bench with ethanol and bleach. The PCR plates were prepared in two dedicated rooms with UV- and bleach sterilized cabinets: one specific for master mix preparation where extracted DNA samples cannot enter, and a second room for DNA addition.

**Data Analysis**

***Data Cleaning and Contaminant Filtering***

Illumina sequencing data were first processed using the DADA2 package version 1.6.0, excluding reads with more than two expected errors or that contained undetermined bases [4]. The first 12 nucleotides of the forward and reverse strand of the paired reads were then trimmed. After DADA2 error correction and removal of chimeras, paired reads were classified using the EZBioCloud 16S database version of the 19th of January 2018 [5] up to species level when possible. This workflow resulted in a table of Amplicon Sequence Variants (ASVs) with a single nucleotide difference resolution. The tidyamplicons R package developed by Stijn Wittouck (<https://github.com/SWittouck/tidyamplicons>) was used for contaminant-filtering and data analysis. For this purpose, the DADA2 outputs of all three runs were first merged into one large tidyamplicons object, followed by the removal of reads longer than 270 bases, non-bacterial reads, reads only detected in the negative controls and reads only detected in a single run which were also present in a negative control (run-specific contaminants). Next, the relative abundance of each taxon remaining in the negative controls was plotted against the sample library size and all taxa whose relative abundance clearly decreased with increasing library size were discarded as contaminants. This was followed by inspection of all taxa with a Spearman-correlation p-value of p < 0.0001 between their relative abundance and the sample post-PCR DNA concentration which lead to filtering out of two more taxa [6]. After all filtering steps, 4650 and 1231 reads remained in the largest and second largest negative controls. Both were extraction controls, while all other negative control samples had less than 875 reads and often no reads left. The largest negative control was considered to be an outlier and twice the number of reads (2462 reads) of the second largest sample and 100 reads per pooled volume were used as quality control cut-off criteria to accept samples for further analysis.

**Isolation**

One hundred microlitres of each clinical sample obtained from healthy children without a history of OME were used for the cultivation of bacteria, with a focus on lactic acid bacteria. Thirty microliters each were plated out on MRS (De Man, Rogosa and Sharpe) agar, M17 agar and BHI (Brain Heart Infusion) agar supplemented with 0.5% (v/v) Tween 80. For anterior nare and nasopharynx samples, a 1:10 dilution (in PBS) was also spread out on the same media. MRS is selective for Lactobacilli and closely related genera, M17 targets *Lactococci* and BHI + 0.5% Tween 80 aimed at isolating *Dolosigranulum pigrum*. Agar plates were incubated at 37°C with 5% CO_2_.

Within the following 7 days, each plate was inspected for growth and one colony of each morphology per plate was transferred to broth for cultivation. Broth cultures were inspected for growth and if present, bacteria were pelleted at 1400 g and 4°C, the pellet was dissolved in 900 µL fresh broth medium, combined with 900 µL of 50% Glycerol and stored as Cryostocks at -80°C in 300 µL Cryo.s^TM^ Biobanking Tubes (Greiner Bio-One). Broth cultures lacking growth after one week were discarded. A long incubation time was chosen to allow for the detection of slowly growing bacteria including *D. pigrum*.

**Identification**

For identification, the isolates were plated out from cryostocks with the streak plate method and incubated for 2.5 days. Then, a small fraction of a colony from each plate was suspended in 10 µL PCR grade water in a PCR tube. Subsequently, the tubes were microwaved for 2 x 1.5 minutes at 800 W to lyse the cells and release the DNA. Fifteen microlitres of master mix consisting of 2.5 µL 10 x VWR Buffer (C_Final_ = 1x), 0.2 µL Taq polymerase, 0.5 µL 10 mM dNPTs (deoxyribonucleotide triphosphates, C_Final_ = 0.2 mM), 2.5 µL each of 10 µM concentrated primers 27F (5’-AGAGTTTGATCMTGGCTCAG-3’) and 1492R (5’-GGTTACCTTGTTACGACTT-3’) (C_Final_ = 1 µM) and 6.8 µl PCR-grate water were added to each tube. After spinning down the contents, the bacterial 16S rRNA gene was amplified in 30 cycles of 30 seconds denaturation at 95°C, 30 seconds annealing at 55°C and 90 seconds of extension at 72°C. Cycling was preceded by a 2-minute initial denaturation step at 95°C and concluded with a 5-minute final extension step at 72°C.

To confirm successful amplification 1 µL of each amplicon were combined with 5 µL of 1.2x loading dye (Thermo Scientific R0611, diluted in PCR-grade H_2_O) and this mixture was loaded into a 0.5% agarose gel containing 1x gel red. The gel was run for 30 minutes at 100 V and visualized under UV light. A GeneRuler^TM^ 1 kb Plus DNA Ladder (Thermo Scientific SM1331) was used as a size standard. Successful samples were submitted to the VIB Genetic Service Facility (Wilrijk, Belgium) for Sanger sequencing, whereby 10 µL amplicon were provided for each read direction, together with either 10 µL of 5 µM of primer 27F or 1492R.

The sequencing data were analysed via SeqTrace 0.9.0 (by Brian Stucky), forward and reverse reads were linked, and the sequences were trimmed to only retain stretches with at least 80% bases reaching a minimum Phred score of 30 (corresponding to a 0.1% error probability). The resulting consensus sequences were classified using the EZBioCloud 16S-based ID tool [5] and the online NCBI Nucleotide Basic Local Alignment Search Tool (BLAST) [8,9]

**Antimicrobial Screening**

The ability of LAB to inhibit the growth of URT pathobionts was tested in two ways: the Spot assay tested the direct effect of live LAB on pathobionts, while the Radial Diffusion assay tested the effect of molecules secreted into the medium during an overnight culture. Most isolates were tested against *M. catarrhalis* ATCC25238, *S. pneumoniae* ATCC49619 and *H. influenzae* ATCC49247. Seven *S. salivarius* isolates were additionally tested against *S. aureus* ATCC29213*, S. pyogenes* BM137, *A. otitidis* DME001 and *T. otitidis* DME002. The last two strains were isolates from OME middle ear effusion. *S. oralis* 89a and *S. salivarius* 24SMB were isolated from the probiotic nasal spray Rinogermina (DMG Italia) and used as references when testing the seven *S. salivarius* isolates.

***Spot Assay***

Broth cultures of *S. salivarius* were incubated overnight and 2 µL of each culture were spotted onto 60 mL pathobiont-specific agar supplemented with glucose to a final concentration of 5 g/L in a square Petri dish. After 48 hours of incubation at 37°C in a 5% CO_2_ atmosphere, the colonies were covered with 20 mL pathobiont-specific soft agar (0.5% w/v) inoculated with the pathobiont from an overnight culture (450 μl *M. catarrhalis*, or 300 μl of *H. influenzae*, *S. pneumoniae*, *S. pyogenes*, *S. aureus*, *C. otitidis* or *A. otitidis*). After 24h incubation at pathobiont-specific conditions, the presence and size of inhibition zones in the lawn of pathobiont growth around the *S. salivarius* spots were evaluated. For each experiment, each *S. salivarius* – pathobiont combination was tested in triplicate [10].

***Well Diffusion Assay***

Overnight broth cultures of pathobionts (600 μl of *M. catarrhalis*, *S. pyogenes*, *S. aureus*, *C. otitidis* or *A. otitidis*, 60 μl of *H. influenzae* or 200 μl of *S. pneumoniae)* were mixed with 60 mL of hand warm agar and poured into square petri-dishes. After drying, wells were punched into the agar. *S. salivarius* overnight cultures were centrifuged for 15 minutes at 1078 g and 4°C, followed by filter-sterilization of the supernatant through a 0.2 µm sterile syringe filter (VWR 514-0061). Thirty microlitres of this cell-free supernatant (CFS) were pipetted into the wells, and after drying the plates were incubated at pathobiont-specific conditions. Inhibition zones around the wells were evaluated after 24h. Fresh sterile medium and 0.1% Hexetidine in the form of the mouthwash Hextril^®^ (Johnson & Johnson) were used as negative and positive controls respectively. For some experiments, the pH of the negative control was decreased to 5 (Streptococci) using HCl, followed by filter-sterilization, to assure that inhibition effects are not just pH based [10].

**Antibiotic Susceptibility Assay**

The minimum inhibitory concentration of Ampicillin, Erythromycin, Tetracycline, Chloramphenicol, Vancomycin, Gentamycin, Streptomycin and Clindamycin on select isolates from the URT of healthy children was tested in a 96-well format. The growth ability of a starting bacterial concentration of 5 x 10^6^ CFU was tested against a twofold serial antibiotic dilution between 0.5 µg/mL and 128 µg/mL. The presence of bacterial growth was evaluated by hourly measurement of the change in optical density at 600 nm. The final Boolean results (growth/no-growth) was recorded after 24 hours. Bacteria without added antibiotic, and sterile medium served as positive and negative controls, respectively.

**Adhesion Assay**

***Cell Culture Maintenance and Seeding***

Calu-3 ATCC® HTB-55™, a human-derived lung adenocarcinoma epithelial cell line, was used to test the adhesion capability of bacterial isolates to human respiratory epithelium (adapted from [11]. The cells were grown in 20 mL Minimal Essential Medium (MEM, Gibco^TM^ 11095080) supplemented with 10% Foetal Bovine Serum (FBS, Gibco 10270-106), 100 U/mL penicillin and 100 µg/mL streptomycin (Pen-Strep) at 37°C in a humidified 5% CO_2_ atmosphere. Cells were maintained in T75 cell culture flasks with filter screw caps (Cellstar® 658175) and split in a 1:2 ratio when grown to ~80% confluence. During growth, the medium was refreshed every 2-3 days.

For splitting, the medium was discarded, the cells were briefly washed with 2 mL 0.25% trypsin (a serine protease) and then incubated for 10 – 15 minutes with 3 mL trypsin to detach the adherend cells from the wall of the flask. This reaction was stopped by adding 10 mL FCS. Then, the cells were pelleted (7 minutes at 300 g), the pellet was resuspended in 10 mL fresh medium and distributed evenly to 2 new cell-culture flasks which already contained 15 mL fresh medium.

To seed cells for an adhesion experiment, they were detached and pelleted as usual, but the pellets of three flasks were combined in 14 mL of MEM + FCS. The concentration of viable cells was then measured with an EVE™ Automatic Cell Counter (NanoEnTek). Cells were seeded in a 24-well cell culture plate (CellStar®) at a density of 3 x 10^5^ cells/cm^2^ using a volume 0.5 mL. The medium was refreshed every 2 days and replaced with pure MEM on the day before the experiment, which was performed after ~1 week of incubation.

***Adhesion Assay***

A volume of bacterial overnight culture containing 2*10^8^ CFU was pelleted for 10 minutes at 2000 g and 4°C. The pellet was washed twice with 1 mL PBS, using the same centrifugation conditions, and finally resuspended in 2 mL pre-warmed MEM. After aspiration of the cell culture medium, 5*10^7^ CFU (500 µL) each were added to triplicate wells seeded with Calu-3 cells. After 1h incubation at 37°C and 5% CO_2_, unattached bacteria were removed by washing the cells once with PBS. This was followed by trypsinization for 15 min at 37°C and 5% CO_2_ to detach the Calu-3 cells and the adherend bacteria. Triplicate ten-fold serial dilutions in PBS of left-over cell-suspension and of adherend cells were plated on medium appropriate for the tested bacterial isolate and incubated at appropriate conditions. Colonies were counted after incubation and the adhesion percentage was calculated by comparing the number of CFU added to the cells to the number of CFU retrieved after the adhesion experiment.

**Whole Genome Sequencing**

***DNA Extraction***

To extract bacterial DNA from 3 mL overnight culture for whole genome sequencing (WGS), cells were first disrupted through incubation with 0.1 mg/mL Ampicillin for at least one hour (longer for slow-growing isolates). The cells were then pelleted (3 minutes at 12 000 g), washed three times with NaCl-EDTA at pH 8 (30 mM Sodium Chloride + 2 mM Ethylenediaminetetraacetic acid) and resuspended in 100 µL NaCl-EDTA. Subsequently, the bacterial cell wall and RNA were further degraded by adding 1 mg of lysozyme and 20 µg of RNase and incubating for 1 hour at 37°C under shaking. Proteins were then denatured and degraded for 1h at 55°C by adding Sodium Dodecyl Sulphate (SDS; C_Final_ = 1%) and Proteinase K (C_Final_ = 0.8 mg/mL) and NaCl-EDTA (to a final volume of 500 µL). This was followed by precipitation with 200 µL cold protein precipitation solution (6 mL 5 M potassium acetate, 1.15 mL glacial acetic acid and 2.85 ml distilled water) with 5 minutes incubation on ice, followed by two 3 minutes centrifugation steps at 4°C and 12 000 g, with transfer of the supernatant to a fresh 1.5 mL Eppendorf tube between steps. The pellet was resuspended in 600 µL ice-cold isopropanol and the precipitating DNA was pelleted at 12 000 g and 4°C for 3 minutes, washed once with 600 µL of 70% ethanol and left to air-dry. The dried DNA was then dissolved in 100 µL H_2_O, aided by incubation at 55°C for 5 minutes. This protocol was based on protocol P3 by [12].

The concentration and purity of the extracted DNA was determined using a Take3^TM^ Micro-Volume Plate on a Synergy^TM^ HTX Multi-Mode Reader (Biotek) and the DNA was stored at -20°C until WGS was performed.

***Sequence Analysis***

The extracted DNA was sequenced on an Illumina MiSeq platform by the Lab of Medical Microbiology at the Antwerp University. The resulting paired-end. fastq files were analysed in two ways:

1. They were submitted to the Comprehensive Genome Analysis pipeline of the Pathosystems Resource Integration Center (PATRIC) website [13] which assembled and annotated the reads with SPAdes ([14] and the RAST toolkit (RASTtk, [15], respectively. Virulence factors and antibiotic resistance genes identified against the Comprehensive Antibiotic Resistance Database (CARD [16] and the Virulence Factor Database (VFDB [17] were considered.

2. They were analysed by the automated in-house pipeline (Sander Wuyts, 2019). After *de novo* assembly with SPAdes-based Shovill (<https://github.com/tseemann/shovill>) and quality control with checkM [18] all genomes with at least ≥94% completeness were annotated with Prokka [19]. Assembled contigs were subsequently searched for the presence of transferable antibiotic resistance genes against the ResFinder 3.2 database, [20], and for virulence factors against the Virulence Factor Database (VFDB; [17] using ABRicate (<https://github.com/tseemann/abricate>). Secondary metabolites were identified with the help of antiSMASH 5.0 [21] and, outside the automated pipeline, BAGEL4 [22], and genomes were submitted to PHASTER (PHAge Search Tool Enhanced Release; [23] for identification of prophages.

**References**

1. Kozich JJ, Westcott SL, Baxter NT, Highlander SK, Schloss PD. Development of a Dual-Index Sequencing Strategy and Curation Pipeline for Analyzing Amplicon Sequence Data on the MiSeq Illumina Sequencing Platform. Appl Environ Microbiol. **2013**; 79(17):5112–5120.

2. Wouters I, Heirstraeten LV, Desmet S, et al. Nasopharyngeal S. pneumoniae carriage and density in Belgian infants after 9 years of pneumococcal conjugate vaccine programme. Vaccine. **2018**; 36(1):15–22.

3. Wouters I, Desmet S, Van Heirstraeten L, et al. Follow-up of serotype distribution and antimicrobial susceptibility of Streptococcus pneumoniae in child carriage after a PCV13-to-PCV10 vaccine switch in Belgium. Vaccine. **2019**; 37(8):1080–1086.

4. Callahan BJ, McMurdie PJ, Rosen MJ, Han AW, Johnson AJA, Holmes SP. DADA2: High-resolution sample inference from Illumina amplicon data. Nat Methods. **2016**; 13(7):581–583.

5. Yoon S-H, Ha S-M, Kwon S, et al. Introducing EzBioCloud: a taxonomically united database of 16S rRNA gene sequences and whole-genome assemblies. Int J Syst Evol Microbiol. **2017**; 67(5):1613–1617.

6. Jervis-Bardy J, Leong LEX, Marri S, et al. Deriving accurate microbiota profiles from human samples with low bacterial content through post-sequencing processing of Illumina MiSeq data. Microbiome. **2015**; 3(1):19.

7. Oksanen J, Blanchet GF, Friendly M, et al. Vegan: Community Ecology Package [Internet]. 2019. Available from: https://CRAN.R-project.org/package=vegan

8. Altschul SF, Gish W, Miller W, Myers EW, Lipman DJ. Basic Local Alignment Search Tool. J Mol Biol. **1990**; 215:403–410.

9. Johnson M, Zaretskaya I, Raytselis Y, Merezhuk Y, McGinnis S, Madden TL. NCBI BLAST: a better web interface. Nucleic Acids Res. **2008**; 36(Web Server):W5–W9.

10. Broek MFL van den, De Boeck I, Claes IJJ, Nizet V, Lebeer S. Multifactorial inhibition of lactobacilli against the respiratory tract pathogen *Moraxella catarrhalis*. Benef Microbes. **2018**; 9(3):429–439.

11. Lebeer S, Claes I, Tytgat HLP, et al. Functional Analysis of Lactobacillus rhamnosus GG Pili in Relation to Adhesion and Immunomodulatory Interactions with Intestinal Epithelial Cells. Appl Environ Microbiol. **2012**; 78(1):185–193.

12. Alimolaei M, Golchin M. A comparison of methods for extracting plasmids from a difficult to lyse bacterium: Lactobacillus casei. Biologicals. **2017**; 45:47–51.

13. Wattam AR, Abraham D, Dalay O, et al. PATRIC, the bacterial bioinformatics database and analysis resource. Nucleic Acids Res. **2014**; 42(Database issue):D581–D591.

14. Bankevich A, Nurk S, Antipov D, et al. SPAdes: A New Genome Assembly Algorithm and Its Applications to Single-Cell Sequencing. J Comput Biol. **2012**; 19(5):455–477.

15. Brettin T, Davis JJ, Disz T, et al. RASTtk: A modular and extensible implementation of the RAST algorithm for building custom annotation pipelines and annotating batches of genomes. Sci Rep. **2015**; 5(1):8365.

16. McArthur AG, Waglechner N, Nizam F, et al. The Comprehensive Antibiotic Resistance Database. Antimicrob Agents Chemother. **2013**; 57(7):3348–3357.

17. Chen L, Zheng D, Liu B, Yang J, Jin Q. VFDB 2016: hierarchical and refined dataset for big data analysis—10 years on. Nucleic Acids Res. **2016**; 44(Database issue):D694–D697.

18. Parks DH, Imelfort M, Skennerton CT, Hugenholtz P, Tyson GW. CheckM: assessing the quality of microbial genomes recovered from isolates, single cells, and metagenomes. Genome Res. **2015**; 25(7):1043–1055.

19. Seemann T. Prokka: rapid prokaryotic genome annotation. Bioinformatics. **2014**; 30(14):2068–2069.

20. Zankari E, Hasman H, Cosentino S, et al. Identification of acquired antimicrobial resistance genes. J Antimicrob Chemother. **2012**; 67(11):2640–2644.

21. Blin K, Shaw S, Steinke K, et al. antiSMASH 5.0: updates to the secondary metabolite genome mining pipeline. Nucleic Acids Res. **2019**; 47(W1):W81–W87.

22. Jong A de, Hijum SAFT van, Bijlsma JJE, Kok J, Kuipers OP. BAGEL: a web-based bacteriocin genome mining tool. Nucleic Acids Res. **2006**; 34(Web Server):W273–W279.

23. Arndt D, Grant JR, Marcu A, et al. PHASTER: a better, faster version of the PHAST phage search tool. Nucleic Acids Res. **2016**; 44(W1):W16–W21.
