# Supplementary material for: Case-Control Microbiome Study of Chronic Otitis Media with Effusion in Children Points at Streptococcus salivarius as a Pathobiont-Inhibiting Species
Source: mSystems. 2021 Apr 20;6(2):e00056-21. doi: 10.1128/mSystems.00056-21 (PMC8546964; doi:10.1128/mSystems.00056-21)
Supplement: TABLE S5 [file msystems.00056-21-st005.docx]

**Table S5**: Cultivation conditions

| **Samples or Target Bacteria** | **Growth Medium** | **Atmosphere** |
| --- | --- | --- |
| Plating out of healthy control samples (cochlear implant group) | | |
| Lactobacilli | MRS | 5% CO_2_ |
| Lactococci | M17 | 5% CO_2_ |
| *Dolosigranulum pigrum* | BHI + 5% (v/v) Tween 80 | 5% CO_2_ |
| Respiratory and middle ear pathogens | | |
| *Alloiococcus otitidis* | BHI + 5% horse blood | Aerobic (shaking) |
| *Corynebacterium otitidis* | BHI + 0.5% Tween 80 | 5% CO_2_ |
| *Haemophilus influenzae* | MH + 0.5% yeast extract + 15 mg NAD + 15 mg Hemin | 5% CO_2_ |
| *Moraxella catarrhalis* | MH |  |
| *Staphylococcus aureus* | MH or MRS | Aerobic |
| *Streptococcus pneumoniae* | TH | 5% CO_2_ |
| *Streptococcus pyogenes* | TH + 0.2% (w/v) yeast extract |  |
| (Potential) probiotics | | |
| *Streptococcus salivarius* | TH | Aerobic or 5% CO_2_ |
| *Streptococcus oralis* | TH | Aerobic or 5% CO_2_ |
| Note: All bacteria were incubated at 37°C. BHI, Brain Heart Infusion; MH, Mueller Hinton; MRS, De Man, Rogosa and Sharpe; TH, Todd Hewitt; NAD, Nicotinamide adenine dinucleotide. | | |
